# Supplementary material for: Pooled prevalence and factors of overweight/obesity among women of reproductive age in low and middle-income countries with high maternal mortality: A multi-level analysis of recent demographic and health surveys
Source: PLoS One. 2025 Oct 8;20(10):e0316962. doi: 10.1371/journal.pone.0316962 (PMC12507220; doi:10.1371/journal.pone.0316962)
Supplement: Attachment — Submitted filename: PONE-D-23-42732-Annotated.pdf [file pone.0316962.s002.pdf]

PLOS ONE

Pooled prevalence and determinants of overweight/obesity among reproductive age women in countries with high maternal mortality: A multi-level analysis of recent Demographic and Health Surveys

--Manuscript Draft--

**Manuscript Number:**	PONE-D-23-42732
**Article Type:**	Research Article
**Full Title:**	Pooled prevalence and determinants of overweight/obesity among reproductive age women in countries with high maternal mortality: A multi-level analysis of recent Demographic and Health Surveys
**Short Title:**	Pooled prevalence and determinants of overweight/obesity among reproductive age women
**Corresponding Author:**	Demiss Mulatu Geberu<br>University of Gondar College of Medicine and Health Sciences<br>Gondar, ETHIOPIA
**Keywords:**	BMI, overweight, obesity, reproductive age women, countries with high maternal mortality
**Abstract:**	Background: Due to the increased magnitude of overweight/obesity in many countries, the World Health Organization (WHO) characterized it as a pandemia of major public health concern and it was progressively higher in reproductive age women at Low and Middle Income Countries (LMICs). Despite obesity/overweight among reproductive age women being well recognized as a common public health problem, its pooled prevalence and different determinants have been less investigated in countries with high maternal mortality. Thus, the objective of our study was to assess the pooled prevalence and determinants of overweight/obesity among reproductive age women in countries with high maternal mortality.<br>Methods: Secondary data analysis was conducted using data from recent Demographic and Health Survey datasets from 21 countries with high maternal mortality. A total of 64,076 (weighted sample) reproductive age women were included in the analysis. Variables were extracted from the IR file and STATA version 14.2 software was used to clean, recode and analyze the data. The multilevel binary logistic regression model was fitted, and AOR with a 95% CI and p-value ≤0.05 was used to declare significantly associated factors. To check model fitness and model comparison; ICC, MOR, PCV, and deviance (−2LLR) were used.<br>Result: In this study, pooled prevalence of overweight/obesity among reproductive age women was 32% (95% CI: 27- 37) with a large variation between countries which ranged from 10% in Burundi to 53% in Mauritania. Compared to reproductive age women with normal BMI, the following several determinants had a significant possitive association with overweight/obesity. Women aged 25–34 years and 35-49 years, women who had primary education, who had secondary education and above, not-working women, married, widowed, and divorced women, poorer, middle, richer, and richest household, female household head, having one child, 2-3 children, 4 and above children, watching television less than once a week and at least once a week, using internet less than once a week and at least once a week, improved source of drinking water, urban residents, low community poverty, and high community level media usage.<br>Conclusions and recommendations: More than three out of ten reproductive age women were overweight/obese in countries with high maternal mortality. Individual-level and community-level factors were associated with overweight/obesity. Special attention is recommended for aged women, those who had attended formal education, not working women, who are watching television and using internet, urban residents, female household head, and richest women.
**Order of Authors:**	Demiss Mulatu Geberu
	Kaleab Mesfin Abera
	Yawkal Tsega

Powered by Editorial Manager® and ProduXion Manager® from Aries Systems Corporation

	Abel Endawkie
	Wubshet Debebe Negash
	Amare Mesfin Workie
	Lamrot Yohannes
	Mihret Getnet
	Nigusu Worku
	Adina Yeshambel
	Lakew Asmare
	Hiwot Tadesse Alemu
	Misganaw Guadie Tiruneh
	Asebe Hagos
	Melak Jejaw Fitigu
	Kaleb Assegid Demissie
**Opposed Reviewers:**	
**Additional Information:**	
**Question**	**Response**
**Financial Disclosure**<br><br>Enter a financial disclosure statement that describes the sources of funding for the work included in this submission. Review the submission guidelines for detailed requirements. View published research articles from *PLOS ONE* for specific examples.<br><br>This statement is required for submission and **will appear in the published article** if the submission is accepted. Please make sure it is accurate.<br><br>**Funded studies**<br>Enter a statement with the following details:<br>• Initials of the authors who received each award<br>• Grant numbers awarded to each author<br>• The full name of each funder<br>• URL of each funder website<br>• Did the sponsors or funders play any role in the study design, data collection and analysis, decision to publish, or preparation of the manuscript?<br><br>Did you receive funding for this work?	The author(s) received no specific funding for this work.

**Competing Interests**<br><br>Use the instructions below to enter a competing interest statement for this submission. On behalf of all authors, disclose any competing interests that could be perceived to bias this work—acknowledging all financial support and any other relevant financial or non-financial competing interests.<br><br>This statement is required for submission and **will appear in the published article** if the submission is accepted. Please make sure it is accurate and that any funding sources listed in your Funding Information later in the submission form are also declared in your Financial Disclosure statement.<br><br>View published research articles from *PLOS ONE* for specific examples.<br><br>**NO authors have competing interests**<br><br>Enter: *The authors have declared that no competing interests exist.*<br><br>**Authors with competing interests**<br><br>Enter competing interest details beginning with this statement:<br><br>*I have read the journal's policy and the authors of this manuscript have the following competing interests: [insert competing interests here]*<br><br>* typeset	The authors have declared that no competing interests exist
**Ethics Statement**<br><br>Enter an ethics statement for this submission. This statement is required if the study involved:<br><br>• Human participants<br>• Human specimens or tissue<br>• Vertebrate animals or cephalopods	We requested the DHS programme to access the data through online at http://www.dhsprogram.com and permission was obtained to download and use data freely. International and national ethical guidelines were taken into consideration during the original collection of DHS data. Ethical clearance for the original DHS was approved by the ICF Macro Institutional Review Board, the Centers for Disease Control and Prevention (CDC) and Institutional Review Board (IRB) in each country, in accordance with United states Department of Health and Human Services requirements for human subject protection. In accordance with the DHS, all respondents and/or their legal guardian(s) of minors' (age below 16) provided written

• Vertebrate embryos or tissues<br>• Field research<br><br>Write "N/A" if the submission does not require an ethics statement.<br><br>General guidance is provided below. Consult the submission guidelines for detailed instructions. **Make sure that all information entered here is included in the Methods section of the manuscript.**	consent to participate. All the methods were conducted according to Helsinki declarations. It is assured that procedures for DHS public-use datasets do not in any way allow respondents, households, or sample communities to be identified. There are no personal identifiers of individuals or household addresses in the data files. The study is not an experimental study. Further explanation of how the DHS uses data and its ethical standards can be found at: http://goo.gl/ny8T6X.

Powered by Editorial Manager® and ProduXion Manager® from Aries Systems Corporation

**Format for specific study types**<br><br>**Human Subject Research (involving human participants and/or tissue)**<br>• Give the name of the institutional review board or ethics committee that approved the study<br>• Include the approval number and/or a statement indicating approval of this research<br>• Indicate the form of consent obtained (written/oral) or the reason that consent was not obtained (e.g. the data were analyzed anonymously)<br><br>**Animal Research (involving vertebrate animals, embryos or tissues)**<br>• Provide the name of the Institutional Animal Care and Use Committee (IACUC) or other relevant ethics board that reviewed the study protocol, and indicate whether they approved this research or granted a formal waiver of ethical approval<br>• Include an approval number if one was obtained<br>• If the study involved *non-human primates*, add *additional details* about animal welfare and steps taken to ameliorate suffering<br>• If anesthesia, euthanasia, or any kind of animal sacrifice is part of the study, include briefly which substances and/or methods were applied<br><br>**Field Research**<br><br>Include the following details if this study involves the collection of plant, animal, or other materials from a natural setting:<br>• Field permit number<br>• Name of the institution or relevant body that granted permission	
**Data Availability**<br><br>Authors are required to make all data underlying the findings described fully available, without restriction, and from the time of publication. PLOS allows rare exceptions to address legal and ethical concerns. See the PLOS Data Policy and FAQ for detailed information.	Yes - all data are fully available without restriction

A Data Availability Statement describing where the data can be found is required at submission. Your answers to this question constitute the Data Availability Statement and **will be published in the article**, if accepted.<br><br>**Important:** Stating 'data available on request from the author' is not sufficient. If your data are only available upon request, select 'No' for the first question and explain your exceptional situation in the text box.<br><br>Do the authors confirm that all data underlying the findings described in their manuscript are fully available without restriction?	
**Describe where the data may be found in full sentences. If you are copying our sample text, replace any instances of XXX with the appropriate details.**<br><br>• If the data are **held or will be held in a public repository**, include URLs, accession numbers or DOIs. If this information will only be available after acceptance, indicate this by ticking the box below. For example: *All XXX files are available from the XXX database (accession number(s) XXX, XXX.*).<br>• If the data are all contained **within the manuscript and/or Supporting Information files**, enter the following: *All relevant data are within the manuscript and its Supporting Information files.*<br>• If neither of these applies but you are able to provide **details of access elsewhere**, with or without limitations, please do so. For example:<br><br>*Data cannot be shared publicly because of [XXX]. Data are available from the XXX Institutional Data Access / Ethics Committee (contact via XXX) for researchers who meet the criteria for access to confidential data.*<br><br>*The data underlying the results presented in the study are available from (include the name of the third party*	Data are available online in a public, open access repository (www.measuredhs.com/data).

and contact information or URL).<br>• This text is appropriate if the data are owned by a third party and authors do not have permission to share the data.<br><br>* typeset	
Additional data availability information:	

Powered by Editorial Manager® and ProduXion Manager® from Aries Systems Corporation

To: **PLOS ONE Editorial office**

Subject: Submission of a manuscript for publication

Dear: PLOS ONE editorial office,

We are happy to submit our manuscript entitled **"Pooled prevalence and determinants of overweight/obesity among reproductive age women in countries with high maternal mortality: A multi-level analysis of recent Demographic and Health Surveys"** to your esteemed journal for publication consideration. We certify that we have participated sufficiently in the intellectual content, conception, analysis, and interpretation of the data, as well as the writing of the manuscript to take public responsibility for it and have agreed to have our name listed as a contributor. Moreover, all authors gave the right to the corresponding author to make necessary changes as per the request of the journal, do the rest of the correspondence, and I will act as the guarantor for the manuscript on our behalf. Furthermore, we ensure that this is our original work and we also declare that we do not have any competing interest. Hence, it is to request you for considering it in your journal for publication.

Thank you in advance!

Demiss Mulatu Geberu

mulatu.demissma23@gmail.com

Corresponding author

Manuscript

Click here to access/download;Manuscript;Manuscript for PLOS ONE updated.docx

Pooled prevalence and determinants of overweight/obesity among reproductive age women in countries with high maternal mortality: A multi-level analysis of recent Demographic and Health Surveys

Demiss Mulatu Geberu*[1], Kaleab Mesfin Abera[2], Yawkal Tsega[3], Abel Endawkie[4], Wubshet Debebe Negash[1], Amare Mesfin Workie[5], Lamrot Yohannes[6], Mihret Getnet[7,8], Nigusu Worku[1], Adina Yeshambel[1], Lakew Asmare[4], Hiwot Tadesse Alemu[1], Misganaw Guadie Tiruneh[1], Asebe Hagos[1], Melak Jejaw Fitigu[1], Kaleb Assegid Demissie[1]

[1]Department of Health Systems and Policy, Institute of Public Health, College of Medicine and Health Sciences, University of Gondar, Gondar, Ethiopia.

[2]Department of Health Systems and Policy, Institute of Public Health, College of Medicine and Health Sciences, Wollo University, Dessie, Ethiopia.

[3]Department of Health System and Management, School of Public Health, College of Medicine and Health Sciences, Wollo University, Dessie, Ethiopia.

[4]Department of Epidemiology and Biostatistics, School of Public Health, College of Medicine and Health Sciences, Wollo University, Dessie, Ethiopia.

[5]Departement of Nutrition, Institute of Public Health, College of Medicine and Health Sciences, University of Gondar, Gondar, Ethiopia.

[6]Department of Environmental and Occupational Health and Safety, Institute of Public Health, University of Gondar, Gondar, Ethiopia.

[7]Department of Human Physiology, School of Medicine, College of Medicine and Health Sciences, University of Gondar, Gondar, Ethiopia

[8]Department of Epidemiology and biostatistics, Institute of public Health, College of Medicine and Health Sciences, University of Gondar, Gondar, Ethiopia

Email addresses:

DMG: mulatu.demissma23@gmail.com

KMA: kaleabmesfin801@gmail.com

YT: yawkaltsega@gmail.com

AE: abelendawkie@gmail.com

WDN: wubshetdn@gmail.com

AMW: amare7595@gmail.com

LY: lamrotyohannes@gmail.com

MG: mihretgetnet1@gmail.com

NW: nigusuworku29@gmail.com

AY: adinayeshambel2012@gmail.com

LA: lakewt071981@gmail.com

HTA: tadessehalemu@gmail.com

MGT: mguadie41@gmail.com

AH: asebehagos21@gmail.com

MJF: melak4903@gmail.com

KAD: kalebassegid2019@gmail.com

***Correspondence:**

Demiss Mulatu Geberu

Email : mulatu.demissma23@gmail.com

P.O. Box: 196, Gondar, Ethiopia.

ABSTRACT

Background: Due to the increased magnitude of overweight/obesity in many countries, the World Health Organization (WHO) characterized it as a pandemia of major public health concern and it was progressively higher in reproductive age women at Low and Middle Income Countries (LMICs). Despite obesity/overweight among reproductive age women being well recognized as a common public health problem, its pooled prevalence and different determinants have been less investigated in countries with high maternal mortality. Thus, the objective of our study was to assess the pooled prevalence and determinants of overweight/obesity among reproductive age women in countries with high maternal mortality.

Methods: Secondary data analysis was conducted using data from recent Demographic and Health Survey datasets from 21 countries with high maternal mortality. A total of 64,076 (weighted sample) reproductive age women were included in the analysis. Variables were extracted from the IR file and STATA version 14.2 software was used to clean, recode and analyze the data. The multilevel binary logistic regression model was fitted, and AOR with a 95% CI and p-value ≤0.05 was used to declare significantly associated factors. To check model fitness and model comparison; ICC, MOR, PCV, and deviance (−2LLR) were used.

Result: In this study, pooled prevalence of overweight/obesity among reproductive age women was 32% (95% CI: 27- 37) with a large variation between countries which ranged from 10% in Burundi to 53% in Mauritania. Compared to reproductive age women with normal BMI, the following several determinants had a significant possitive association with overweight/obesity. Women aged 25–34 years and 35-49 years, women who had primary education, who had secondary education and above, not-working women, married, widowed, and divorced women, poorer, middle, richer, and richest household, female household head, having one child, 2-3 children, 4 and above children, watching television less than once a week and at least once a week, using internet less than once a week and at least once a week, improved source of drinking water, urban residents, low community poverty, and high community level media usage.

Conclusions and recommendations: More than three out of ten reproductive age women were overweight/obese in countries with high maternal mortality. Individual-level and community-level factors were associated with overweight/obesity. Special attention is recommended for aged

women, those who had attended formal education, not working women, who are watching television and using internet, urban residents, female household head, and richest women.

Keywords: BMI, overweight, obesity, reproductive age women, countries with high maternal mortality

Background

Overweight and abesity are growing public health problems globally. Due to the increased magnitude of the problem in many countries, the WHO characterized it as a pandemia of major public health concern (1). It is ranked as the sixth leading cause of disability-adjusted life years (DALYs) and accounts for approximately 4.0 million deaths worldwide (2-4). Besides, the global mortality associated with overweight/obesity increased by 28.3% from 1990 to 2015 (3). Several previous studies have shown, following marked dietary change, overweight/obesity is increasing more rapidly in LMICs compared to higher-income countries (4, 5). A previous study conducted in 2013 confirmed the high burden of overweight/obesity in low and middle income countries as two billion persons world wide were overweight/obese and sixty two percent of the world's obese population were dwellers of developing countries (6).

However, several studies depicted that a much higher prevalence has been observed in women (3-5, 7-11). Although LMICs are affecting by the double burden of overweight/obesity and underweight, the magnitude of overweight exceeded that of underweight on adult women (12). A study carried out in 37 LMICs revealed that overweight was higher progressively in reproductive age women (13). Previous studies found that fifty three percent of all deaths in women was directly attributed with overweight/obesity (14) and it is a well-documented risk factor for obstetric complications for both the reproductive age women and their future newborn. The complications in reproductive age women include diabetes mellitus (8, 15-18), hypertension (17-19), Chronic Kidney Disease (CKD) (3, 20), Cogestive Heart Failure (CHF) (3, 21), cesarean delivery and surgical site infection (17, 18, 22-24), miscarriage and stillbirth (25), fetal macrosomia (26, 27), breast and cervical cancers (18), postpartum endometritis (28), prolonged hospital stay and postpartum hemorrhage (17, 22). For newborns, low birth weight, congenital malformation, preterm birth, large-for- gestational- age babies, and perinatal death are some of the complications (18, 29, 30).

Different studies revealed that the magnitude of overweight/obesity in reproductive age women was 18% in Cambodia (31), 19.4% in Timor-Leste (32), 26.9% in Mali (33), 35.5% in Ghana (34), 43% in Bangladesh (35), 50.4% in Tanzania (36), and 55.2% in Brazil (37). Nevertheless a range of interventions such as restricting the advertisement of unhealthy foods, improving school meals, using taxation to reduce consumption of unhealthy foods, and using supply chain incentives to

increase production of healthy foods were implemented, to minimize and curve the burden, its prevalence is still highly increasing from time to time unacceptably in reproductive age women (3). Findings of some trend analysises ascertained this rapid progress of overweight/obesity. Accordingly, the magnitude of overweight/obesity of reproductive age women in Bangladesh increased from 9.35% in 1999 to 39.14% in 2014 (7) and increased from 25% in 2005 to 36.6% in 2015 at Zimbabwe (38). The other recent study also revealed that it was increased from 20.5% in 2006 to 41.1% in 2016 at Nepal (39).

Various studies showed that several factors contributed to overweight/obesity among reproductive age women. These include high socioeconomic status (1, 8, 10, 18, 33-35, 38-45), age (1, 9, 10, 18, 33, 35-41, 43), parity (37, 39), marital status (8, 10, 31, 33, 36-38), education (10, 31, 33, 34, 39-41, 46, 47), occupation status (46), urban residence (1, 8, 10, 31, 38, 41, 45), number of household members (7), contraceptive use (40, 48), number of children (31, 45), and watching television (32, 33). Additionally, behavioral factors such as alcohol drinking and smoking cigarettes were potential factors (49, 50). Moreover, the burden of overweight/obesity is attributed by nutritional transition from traditional meals which consisted whole foods like grains that have low animal source foods, salt, refind oils and sugars (51) to energy dense and nutrient-poor diets composed of refind carbohydrates, high fate intake, and processed foods (52). As a result, frequent consumption of sweets, meat and eggs, and eating a snack were the determinants of increased overweight/obesity among reproductive age women (1, 8, 38). Other studies also revealed that obesity/overweight is determined by lower physical activities (8, 18, 36, 47) and ethnicity (8).

Despite obesity/overweight among reproductive age women being well recognized as a ~~common~~ public health problem, its pooled prevalence and different determinants have been less investigated in countries with high maternal mortality using recent Demographic and Health Servey (DHS) data. Besides, as the global community strives to develop curation and prevention policies to address the burden of overweight/obesity, timely sufficient information about its rapid progress and health impacts ~~at reproductive age women~~ in countries with high maternal mortality is needed. In this regard, to prevent obesity/overweight-related mortality and morbidity in reproductive age women, healthcare providers and policy-makers should identify and implement evidence-based interventions. It is also essential to insight the reproductive age women and the community about the potential risk factors of overweight/obesity and refrain from these risk factors in the future.

Moreover, the finding of this study will supply reliable evidence for WHO's ambitious voluntary target of stopping the increment of obesity by 2025. Thus, the objective of our study was to assess the pooled prevalence and determinants of overweight/obesity among reproductive age women in countries with high maternal mortality.

Materials and methods

Study design, setting, and period

The Demographic and Health Surveys (DHS) used a cross-sectional survey study design to collect data and provide population and health indicators at the national level. In this study, we only included countries which have high maternal mortality and DHS data such as Benin, Burundi, Chad, Congo Democratic Republic, Cote d'Ivoire, Cameroon, Gambia, Guinea, Haitie, Kenya, Liberia, Lesotho, Madagascar, Mali, Mauritania, Malawi, Nigeria, Niger, Sierra Leone, Togo, and Zimbabwe in a study period from 2012 to 2022.

Data source and population

We pooled the data from the DHSs conducted in twenty one (Benin, Burundi, Chad, Congo Democratic Republic, Cote d'Ivoire, Cameroon, Gambia, Guinea, Haitie, Kenya, Liberia, Lesotho, Madagascar, Mali, Mauritania, Malawi, Nigeria, Niger, Sierra Leone, Togo, and Zimbabwe) countries that have high maternal mortality (Table 1). Among countries that have high maternal mortality, four countries don't have DHS reports at all and one country hasn't publically available DHS data. Besides, one country hasn't BMI record and another one country hasn't appropriate BMI record. Multi-country analysis using DHS survey data is simple and reasonable as the surveys follow the same questionnaires, sampling procedure, data collection, and coding (53).

The source population was all reproductive age women (15-49 years) who lived in countries with high maternal mortality. Our study population was all reproductive age women (15-49 years) who lived in countries with high maternal mortality, had valid BMI measurements, and were alive at the time of the survey. For this analysis, we used the individual record (IR) file and we included all reproductive age women (15-49 years) at the time of the survey. Those reproductive age women who were pregnant, who have births in the last five years, and who have BMI measurement were excluded according to the DHS guid. Sample weight (v005/1,000,000) was used to correct for over

and under-sampling and generalizability of the findings. As a result, the total weighted sample size from the pooled (appended) data was 64,076.

Table1: Maternal mortality category and year of the survey by the country

Sr.No	Country	Year of DHS Survey	Maternal mortality/100,000	Category
1.	Benin	2017-18	523	Vey High
2.	Burundi	2016-17	494	High
3.	Chad	2014-15	1063	Extremely High
4.	Congo Democratic Republic	2013-14	547	Very High
5.	Cote d'Ivoire	2021	480	High
6.	Cameroon	2018	438	High
7.	Gambia	2019-20	458	High
8.	Guinea	2018	553	Very High
9.	Haiti	2016-17	350	High
10.	Kenya	2022	530	Very High
11.	Liberia	2019-20	652	Very High
12.	Lesotho	2014	566	Very High
13.	Madagascar	2021	392	High
14.	Mali	2018	440	High
15.	Mauritania	2019-21	464	High
16.	Malawi	2015-16	381	High
17.	Nigeria	2018	1047	Extremely high
18.	Niger	2012	441	High
19.	Siera Leone	2019	443	High
20.	Togo	2013-14	399	High
21.	Zimbabwe	2015	357	High

High:300-499; Very High:500-999; Extremely High:>1000

Definition of variables

Outcome variable:

BMI was the outcome variable for this study.

Independent variables:

Independent variables include the age of women, women's educational status, women's occupation, women's marital status, number of household members, wealth index, using contraceptive method, smoking, sex of hosehold head, number of living children, source of

driniking water, frequency of watching television, reading newspaper/magazine, listening to radio, and of using internet were considered individual-level factors. Whereas, place of residence, distance to health facility, community poverty level, community-level women's education, and community-level media usage were treated as the community-level factors. The last three community-level variables were not directly found in DHS. We generated those by aggregating the selected individual and household level factors at the cluster level and categorized them as higher or lower based on a median value.

Opperational definitions

We dichotomized the BMI as normal and overweight/obesity according to WHO classification (54). Normal BMI is from 18.5 to 24.9 kg/m^2, overweight if BMI ≥25 kg/m^2 and those with BMI ≥30 kg/m^2 were obese.

Community level women's education: It is the proportion of reproductive age women in the community that have primary and above education. It was dichotomized as low if the women had <50% primary and above education and high if the women had ≥ 50% primary and above education (55, 56).

Community poverty: It was dichotomized as low if the women's proportion of low wealth index categories such as poorest and poorer was less than 50% and high if it was greater than or equal to 50% (55, 56).

Community level media usage: It is the proportion of reproductive age women in the community who watched television, listend radio, read newspaper/magazine, and used internet. The categorization was low if the repondents had less than 50% media exposure and high if they had greater than or equal to 50% (55, 56).

Statistical analyses

We append the dataset of each country to generate pooled data. The data extraction, coding, and analysis were carried out using STATA software version 14.2. We weighted the sample to restore the representativeness of the sample so that the total sample looks like the country's actual population. Due to the hierarchical nature of the DHS, the multilevel analysis was fitted to identify significantly associated factors. To assess the variability across the 21 countries, Intraclass

Correlation Coefficient (ICC), and Proportional Change in Variance (PCV), were carried out. Additionally, the Likelihood Ratio test (LR) and Median Odds Ratio (MOR) were computed to measure the variation between clusters. Variables with a p-value ≤0.25 in the bi-variable analysis were included in the multivariable analysis and four models were fitted. The null model (with no predictors), model II (adjusted for individual-level variables only), model III (adjusted for community-level variables only), and model IV (model adjustment for both individual and community-level variables simultaneously) were fitted. Since the models were nested we compared them using deviance (−2 log-likelihood). Adjusted OR (AOR) with 95% CI and p<0.05 were applied to determine significant predictors. The variance inflation factor (VIF) test was done to check multicollinearity and was not found because all variables have VIF <5. The model III VIF was 1.74.

Ethical approval and consent to participate

We requested the DHS programme to access the data through online at http://www.dhsprogram.com and permission was obtained to download and use data freely. International and national ethical guidelines were taken into consideration during the original collection of DHS data. Ethical clearance for the original DHS was approved by the ICF Macro Institutional Review Board, the Centers for Disease Control and Prevention (CDC) and Institutional Review Board (IRB) in each country, in accordance with United states Department of Health and Human Services requirements for human subject protection. In accordance with the DHS, all respondents and/or their legal guardian(s) of minors' (age below 16) provided written consent to participate. All the methods were conducted according to Helsinki declarations. It is assured that procedures for DHS public-use datasets do not in any way allow respondents, households, or sample communities to be identified. There are no personal identifiers of individuals or household addresses in the data files. The study is not an experimental study. Further explanation of how the DHS uses data and its ethical standards can be found at: http://goo.gl/ny8T6X.

RESULTS

Socio-demographic, economic, and health service-related characteristics of respondents

A total of 64,076 reproductive age women were included in the study, of these 47.09% were in the age group of 15-24. Almost half of the participants (50.29%) had attended secondary and above education and about 30.47% of the reproductive age women were richest. Neraly two-third (63.12) of sex of the household head in this study was female. More than half (53.04%) of the respondents resided in rural areas. More than three-fourth (76.11) of the respndents had improved source of drinking water (Table 2).

Table 2. Socio-demographic and health service-related characteristics of respondents in countries with high maternal mortality

Variables	Categories	Unweighted frequency (%)	Weighted frequency (%)
Country	Benin	2586 (4.10)	2584 (4.03)
	Burundi	3117 (4.91)	3008 (4.70)
	Congo Democratic Republic	2751 (4.34)	2906 (4.53)
	Cote d'Ivoire	2927 (4.61)	3027 (4.72)
	Cameroon	3048 (4.81)	3000 (4.68)
	Gambia	2361 (3.72)	2540 (4.00)
	Guinea	2044 (3.22)	2053 (3.20)
	Haiti	5213 (8.21)	5306 (8.28)
	Kenya	7599 (11.98)	8025 (12.53)
	Liberia	1495 (2.36)	1425 (2.22)
	Lesotho	1798 (2.83)	1784 (2.78)
	Madagascar	3528 (5.56)	3570 (5.57)
	Mali	1542 (2.43)	1451 (2.26)
	Mauritania	3180 (5.01)	3181 (4.96)
	Malawi	2981 (4.70)	2914 (4.55)
	Nigeria	3879 (6.12)	3783 (5.90)
	Niger	1131 (1.78)	1023 (1.60)
	Siera Leone	3285 (5.18)	3373 (5.26)
	Chad	2837 (4.47)	2981 (4.65)
	Togo	1941 (3.06)	2038 (3.18)
	Zimbabwe	4186 (6.60)	4100 (6.40)
Age of women	15-24	29884 (47.11)	30174 (47.09)
	25-34	10057 (15.86)	10359 (16.17)

Variables	Categories	Unweighted frequency (%)	Weighted frequency (%)
	35-49	23488 (37.03)	23542 (36.74)
Women educational status	No education	15492 (24.42)	14766 (23.04)
	Primary education	17064(26.90)	17085 (26.66)
	Secondary and above	30873 (48.67)	32225 (50.29)
Women occupation	Not working	24520 (40.36)	24509 (39.81)
	Working	36235 (59.64)	37058 (60.19)
Women marital status	Single	31000 (48.87)	31481 (49.13)
	Married	25492 (40.19)	25549 (39.87)
	Widowed	2598 (4.10)	2579 (4.02)
	Divorced	4339 (6.84)	4467 (6.97)
Number of household members	1-4	20732 (32.68)	21214 (33.11)
	5-10	35455 (55.90)	35640 (55.62)
	>10	7242 (11.42)	7221 (11.27)
Wealth index	Poorest	8757 (13.81)	7785 (12.15)6
	Poorer	9976 (15.73)	9756 (15.23)
	Middle	12575 (19.83)	12108 (18.90)
	Richer	14567 (22.97)	14906 (23.26)
	Rechest	17554 (27.68)	19521 (30.47)
Current contraceptive method	No Method	50638 (79.83)	50821 (79.31)
	Modern Method	11360 (17.91)	11678 (18.22)
	Traditional Method	1431 (22.56)	1577 (2.46)
Smoking	No	60043 (99.1)	60512 (99.10)
	Yes	549 (0.90)	584 (0.9)
Sex of household head	Male	40184 (63.35)	40448 (63.12)
	Female	23245 (36.65)	23628 (36.88)
Number of living children	No child	34455 (54.32)	34906 (54.48)
	One child	4913 (7.75)	5163 (8.10)
	2-3 children	10502 (16.56)	10792 (16.84)
	4 and above children	13559 (21.38)	13215 (20.62)
Frequency of watching television	Not at all	31491 (49.68)	30584 (47.77)
	Less than once aweek	10254 (16.18)	10236 (15.99)
	At least once a week	21641 (34.14)	23207 (36.25)
Frequency of reading newspaper or magazine	Not at all	49409 (77.93)	49253 (76.91)
	Less than once aweek	8305 (13.10)	8662 (13.53)
	At least once a week	5690 (8.97)	6123 (9.56)
Frequency of listening to radio	Not at all	25681 (40.49)	24937 (38.92)
	Less than once aweek	13880 (21.88)	14220 (22.19)
	At least once a week	23868 (37.63)	24918 (38.89)
Frequency of using internet	Not at all	39557 (74.68)	38666 (72.48)
	Less than once aweek	1496 (2.82)	1570 (2.94)

Variables	Categories	Unweighted frequency (%)	Weighted frequency (%)
	At least once a week	11918 (22.50)	13107 (24.57)
Source of driniking water	Unimproved	15970 (25.18)	15304 (23.88)
	Improved	47,456 (74.82)	48,769 (76.11)
Residence	Urban	28811 (45.42)	30088 (46.96)
	Rural	34618 (54.58)	33988 (53.04)
Distance to health facility	Big problem	19736 (32.57)	19224 (31.47)
	Not a big problem	40854 (67.43)	41869 (68.53)
Community-level women education	Low	32068 (50.56)	31242 (48.76)
	High	31361 (49.44)	32833 (51.24)
Community poverty	Low	31633 (49.87)	33598 (52.43)
	High	31796 (50.13)	30478 (47.56)
Community-level media usage	Low	26760 (50.52)	25531 (47.86)
	High	26211 (49.48)	27812 (52.14)

242

Prevalence of overweight/obesity among reproductive age women in countries with high maternal mortality

The pooled prevalence of overweight/obesity among reproductive age women in countries with high maternal mortality was 32% (95% CI: 27 – 37), with the highest in Mauritania (53%) and the lowest in Burundi (10%) (Figure1).

Multilevel logistic regression analysis of overweight/obesity among reproductive age women in countries with high maternal mortality

Model comparison and random effect analysis

The result of random effect analysis ~~in this study indicated~~ that the Inter Cluster Correlation Cofficent (ICC) of the null model was 0.19 which shows that ninetheen percent of total variability in overweight/obesity magnitude was related to between cluster variability, while eighty one percent was due to individual differences. MOR in the null model was 2.30 which depicted that a reproductive age women from a cluster with high overweight/obesity prevalence has 2.30 times higher chance of being affected than a reproductive age women from lower overweight/obesity prevalence. The last model (Model III) was the best fitted model because it has the highest log likelihood (-27625) and the lowest deviance (55250) among all fitted models. Model III has PCV of 29% which indicated that 29% of the total variability in overweight/obesity prevalence was explained by the full model (Table 3).

Fixed effect analysis

To identify the determinants of overweight/obesity among reproductive age women, a multivariable analysis was performed. In this regard, based on the best fitted model (Model III), both the individual and community level variables were significant determinants of overweight/obesity among reproductive age women in countries with high maternal mortality. Accordingly, women aged 25–34 years, women aged 35-49 years, women having primary education, secondary education and above, not working women, married, widowed, and divorced women, poorer, middle, richer, and richest women, female sex of household head, women having one, 2-3, four and above children, women watching television less than once a week and at least once a week, women using internet less than once a week and at least once a week, women having improved source of drinking water were from individuale level determinants. Whereas, urban

residence, low community poverty, and high community-level media usage were significantly associated variables with overweight/obesity among community-level part (Table 3).

Table 3. Multivariable multilevel logistic regression analysis results of both individual-level and community-level factors associated with obesity/overweight in East Africa

Variables	Categories	Null model	Model I AOR [95% CI]	Model II AOR [95% CI]	Model III AOR [95% CI]
Age of women	15-24		1.00	---------------	1.00
	25-34		**2.44 (2.25 - 2.65)*** **	---------------	**2.42 (2.23 - 2.63)*** **
	35-49		**3.49 (3.17 – 3.84)*** **	---------------	**3.47 (3.15 - 3.83)*** **
Women educational status	No education		1.00	---------------	1.00
	Primary education		**1.10 (1.03 - 1.18)** **	---------------	**1.10 (1.02 - 1.18)** **
	Secondary and above		**1.10 (1.02 – 1.19)** **	---------------	**1.10 (1.01 - 1.18)** **
Women occupation	Not working		**1.07 (1.02 – 1.13)* **	---------------	**1.06 (1.01 – 1.12)* **
	Working		1.00	---------------	1.00
Women marital status	Single		1.00	---------------	1.00
	Married		**1.96 (1.81 – 2.13)*** **	---------------	**1.98 (1.82 – 2.15)*** **
	Widowed		**1.39 (1.22 – 1.59)*** **		**1.39 (1.22 – 1.59)*** **
	Divorced		**1.71 (1.54 –1.90)*** **		**1.70 (1.53 –1.88)*** **
Number of household members	1-4		1.00	---------------	1.00
	5-10		1.00 (0.95 – 1.05)	---------------	1.00 (0.95 - 1.05)
	>10		1.03 (0.94 - 1.12)	---------------	1.03 (0.94 - 1.12)
Wealth index	Poorest		1.00	---------------	1.00
	Poorer		**1.33 (1.21 - 1.47)*** **	---------------	**1.30 (1.18 - 1.44)*** **
	Middle		**1.93 (1.75 – 2.13)*** **	---------------	**1.75 (1.59 - 1.94)*** **
	Richer		**2.44 (2.21 -2.70)*** **	---------------	**1.98 (1.77 - 2.21)*** **
	Rechest		**3.16 (2.84 – 3.52)*** **	---------------	**2.38 (2.11 - 2.69)*** **
Current contraceptive method	No Method		0.95 (0.89 – 1.00)	---------------	0.95 (0.89 – 1.00)
	Modern Method		1.00	---------------	1.00
	Traditional Method		1.13 (0.98 – 1.31)	---------------	1.12 (0.97 – 1.30)
Smoking	No		1.00	---------------	1.00
	Yes		0.94 (0.77 – 1.15)	---------------	0.93 (0.76 – 1.14)
Sex of household head	Male		1.00	---------------	1.00
	Female		**1.18 (1.12 - 1.24)*** **	---------------	**1.15 (1.09 - 1.22)*** **
Number of living children	No child		1.00	---------------	1.00
	One child		**1.28 (1.16 - 1.41)*** **	---------------	**1.27 (1.15 - 1.40)*** **
	2-3 children		**1.45 (1.31 - 1.59)*** **	---------------	**1.44 (1.31 - 1.59)*** **
	4 and above children		**1.34 (1.20 - 1.49)*** **	---------------	**1.34 (1.21 - 1.50)*** **
Frequency of watching television	Not at all		1.00	---------------	1.00
	Less than once a week		**1.39 (1.30 - 1.50)*** **	---------------	**1.30 (1.21 - 1.39)*** **

	At least once a week		**1.55 (1.46 - 1.66)***	---------------	**1.41 (1.32 - 1.51)***
Frequency of reading newspaper or magazine	Not at all		1.00	---------------	1.00
	Less than once aweek		1.03 (0.97 – 1.11)	---------------	1.04 (0.97 – 1.11)
	At least once a week		1.00 (0.92 – 1.10)	---------------	1.00 (0.92 – 1.10)
Frequency of listening to radio	Not at all		1.00	---------------	1.00
	Less than once aweek		0.98 (0.92 – 1.05)	---------------	0.97 (0.91 – 1.03)
	At least once a week		0.98 (0.92 – 1.03)	---------------	0.97 (0.92 – 1.03)
Frequency of using internet	Not at all		1.00	---------------	1.00
	Less than once a week		**1.50 (1.32 - 1.71)***	---------------	**1.45 (1.28 - 1.65)***
	At least once a week		**1.74 (1.64 - 1.85)***	---------------	**1.67 (1.57 - 1.78)***
Source of driniking water	Unimproved		1.00	---------------	1.00
	Improved		**1.12 (1.05 - 1.19)***	---------------	**1.08 (1.01 - 1.15)***
**Community-level variables**					
Residence	Urban		---------------	**1.48 (0.38 – 1.58)***	**1.38 (1.28 – 1.49)***
	Rural		---------------	1.00	1.00
Distance to health facility	Big problem		---------------	1.00	1.00
	Not a big problem		---------------	**1.12 (1.07 - 1.18)***	1.04 (0.99 - 1.10)
Community-level women's education	Low		---------------	1.00	1.00
	High		---------------	**1.09 (1.03 - 1.45)**	1.02 (0.95 - 1.09)
Community poverty	Low		---------------	**1.52 (1.42 – 1.63)***	**1.10 (1.01 – 1.20)**
	High		---------------	1.00	1.00
Community-level media usage	Low		---------------	1.00	1.00
	High		---------------	**1.46 (1.38 - 1.55)***	**1.20 (1.12 - 1.29)***
**Random effect**					
	Variance	0.77	0.56	0.48	0.55
	ICC	0.19	0.15	0.13	0.14
	MOR	2.30	2.04	1.93	2.02
	PCV	Reff	27.30	38.00	29.00
**Model comparison**					
	Log likelihood ratio	-38895	-27706	-32282	-27625
	Deviance	77790	55412	64564	55250
	Mean VIF		1.64	1.40	1.74
* = P-value < 0.05, ** = P-value < 0.01, *** = P-value < 0.001					
ICC = Inter cluster corrolation cofficent , MOR =Median odds ratio, PCV = proportional change in variance. AOR=adjusted odds ratio; CI= confidence interval, VIF =variance inflation factor					

Discussion

Overweight/besity in reproductive age women is a major public health problem in low and middle income countries. Prevention and early detection of the case of overweight/obesity has a significant advantage for women and household quality of life. Accordingly, one of the best remarkable method of bringing the attention of each individual, healthcare provider, and policy maker is determining overweight/obesity prevalence and its determinants which would pointout the solution at the root of each cause. Therefore, this study assessed the pooled prevalence of overweight /obesity and its determinants among reproductive age women in countries with high maternal mortality using a recent DHS surveys. Our study found that 32% [95% CI: (27 – 37)] of reproductive age women were overweight/obese, which indicates that more than three out of ten women were overweight/ obese. The result of this study depicted that Mauritania has the highest (53%) prevalence whereas Burundi has the lowest (10%) prevalence of overweight/obesity in reproductive age women. This variation in countries could be explained by differences in socio demographic and economic status, lifestyle variations like food preferences, cultural beliefs about dietary consumption, and disparities in government and policy makers commitment of regulating overweight/obesity (33, 57, 58).

In this study, both individual-level and community-level variables were found to be significantly associated with overweight/obesity among reproductive age women. The determinants include women aged 25–34 , 35-49 years, women attended primary education, secondary education and above, not working reproductive women, married, widowed, and divorced women, poorer, middle, richer, and richest women, female sex of household head, women who had one, 2-3, four and above children, women watched television less than once a week and at least once a week, women used internet less than once a week and at least once a week, women who had improved source of drinking water, being urban residence, low community poverty, and high community-level media usage were associated with a higher prevalence of overweight/ obesity.

Consistent with previous studies conducted elsewhere(18, 33, 36-38, 41, 43), in our study, women aged 25-34 years and 35-49 years were more likely to become gain weight. The possible explanation for this positive relationship might be physiological changes following increment of age including hormonal changes, and an increase and redistribution of body fat (41). Besides, the decrement of functional performance associated with physiologic change in aging women can

minimize physical activity which in return leads to higher weight gain (59-61) could be another justification.

This study confirmed the association between women education and overweight/obesity in countries with high maternal mortality. A higher odds of overweight/obesity was observed among reproductive age women with primary, secondary, and higher education compared to no education. This finding is also in agreement with other previous studies elsewhere (7, 9, 34, 41). This could be because educated reproductive age women relatively have a high income, and this might lead to changing lifestyles such as nutritional trends, consumption of high-calorie, and fat diets (4, 41). However, our finding is not similar to other previous studies (62, 63). This discrepancy could be because of methodological differences and the data they used. In our study, we used 21 countries' DHS data and revealed the pooled prevalence of those nations.

The odds of overweight/obesity among not working reproductive age women was high compared to those working women. This finding is consistent with other studies carriedout in Ethiopia and Bangladesh ((35, 43). This could be attributed with low occupation related physical activity in women who are not working which in return increases weight gain (64, 65). Besides, the other possible justification might be the association of not working women and high frequency of watching television or using interenet. In this regard, previous studies suggested that there is high interation between television watching and sedentary life, consequently, leads to high weight gain (33, 66). Therefore, not working reproductive age women might have less exposure while they stay home due to the absence of work compared to their counterparts and could increase their chance to gain weight.

In countries with high maternal mortality, frequency of watching television and using internet from individual level factors and community-level media usage from community level factor have a significant relationship with overweight/obesity among reproductive age women. Women who have higher frequency of watching television, using internet,and community-level media usage were more likely to become obese or gain weight. This finding is in agreement with studies conducted in Mali (33) and Timore-Leste (32). Frequent watching of television and using interenet as well as high community media usage could be related with abnormal metabolism associated with this sedentary life (66), then consequently, brings higher weight gain (67) might be the potential justification. Additionally, frequent use of media such as watching television, using

internet and community media might replace the time which would be allotted for physical activity. Consequently, this could resulted in low energy expenditure and leads to higher weight gain (68), might be another possible justification.

This study revealed that wealth quantile has direct relationship with ovwerweight/obesity. As the hosehold income increase, the odds of weight gain increase. Besides, from the community level factors, lower community poverty was significantly associated with higher odds of weight gain in reproductive age women. Our finding is consistent with previous several studies(8, 33-41, 43-45). The possible justification for this could be that in the context of developing countries, the richest are more likely to consume energy-dense foods and their lifestyle is sedentary (40, 69). In addition, richest women might have increased caloric intake than poorest, and this could primarily be responsible for weight gain.

The odds of overweight/obesity among urban reproductive age women was higher compared with women in the rural area. This finding is supported by previous studies (8, 10, 31, 38, 41). Research revealed that urbanization accounts for significant shifts in dietary patterns. Besides, the availability of fast-food restaurants that provide energy-dense foods (69) and the availability of supermarkets might be associated with overweight/obesity. In addition, it alters physical activity levels as urban residents frequently use transportation rather than walking. On the other hand, rural residents might have great exposure to physical activities as there are no abundant transportations such as a taxi (31). Therefore, due to the above reasons urban residents of reproductive age women could be more subjected to overweight/obesity than rural residents.

In countries with high maternal mortality, overweigt/obesity of married or widowed or, divorced women was higher than single women. This finding is supported by previous ~~several~~ studies ~~conducted elsewhere~~ (8, 10, 31, 33, 36, 38). This could be attributed by married or widowed or, divorced women rather than single women are more likely to deliver more children with their repective pregnancy. As a result, these women are at high risk of weight gain during their pregnancy and postpartum period due to their more sedentary life and taking of energy dense foods (69, 70). Our study also revealed that the odds of overweight/obesity among reproductive age women who had children were higher than women who had no child. This finding is supported by studies carried out in Cambodia (31) and Guinea (45). The possible justification could be women who have children might waste their time and burdened by childcare, consequently, they might

not have appropriate attention for their weight management (31). Additionally, having more children is directly related with repeated pregnancy and this pregnancy will also be linked with depression and anxiety. Therefore, the depression and anxiety might contribute for hypothalamic-pituitary-abnormal hyperactivity which inturn induces abnormal obesity (71).

This study revealed that the odds of overweight/obesity was higher among female household heads than their counterparts. This can be explained by when females being the head of household, they will be suffered from stress, depression, and anxity (72). In this regard, stress, depression, and anxity are linked with abnormal hormonal function which will increases abnormal weight gain (18, 71). Another finding of this study showed that the odds of overweight/obesity were higher amonge reproductive age women who had improved source of drinking water than who had unimproved source. One of the component of improved source of drinking water which has taken the highest share in this study was borehole water. As a matter of fact, this borehole water is salty water. Therefore, women with higher salt intake might have a probability of higher caloric intake, food consumption, eating more meat and snakes which directly predisposes to obesity (73) could be one of the possible justification. Besides, the other possible explanation could be since bottled water was the other component of improved source of drinking water in this study, the chemicals of plastic of the bottles might contributed to develop weight gain. The chemicals might change users metabolism by provoking the growth of fat cells or adipocytes (74). Surprisingly, as a confirmatory finding in this study, 94% of bottled water user reproductive age women had overweight/obesity.

Our study had some strengths and limitations. Among the strengths of this study; it was analyzed using pooled data from 21 nationally representative DHS surveys in countries with high maternal mortality. In addition, we weighted our sample size to get reliable estimates and standard errors. As a limitation, the DHS used a cross-sectional survey design, and the causal relationship between reproductive age women's overweight/obesity and the independent variables cannot be established.

Conclusion and recommendation

The prevalence of overweight/obesity among reproductive age women was relatively high in countries with high maternal mortality. The result indicated that more than three out of ten reproductive age women were overweight/obese. Individual-level and community-level factors

were associated with overweight/obesity. Reproductive age women with older age, higher education, higher wealth status, having children, watching television, using internet, having improved source of drinking water, and urban residents had higher odds of overweight/obesity. The odds of overweight/obesity was also higher in female household heads, not working women, married, widowed, and divorced women. Giving special attention to those groups of reproductive age women who are aged, had attended formal education, not working women, who are watching television and using internet, urban residents, female household head, and richest women are recommended.

Abbreviations

AOR, Adjusted Odds Ratio; BMI, body mass index; CI, Confidence Interval; CDC, Centers for Disease Control and Prevention; CHF, Congestive Heart Failure; CKD, Chronic Kidney Diease; DHSs, Demographic and Health Surveys; DALYs disability-adjusted life years; ICC, Intracluster correlation coefficient; IR, individual record; IRB, Institutional Review Board; LMIC, Low and Middle Income Countries; MOR, median odds ratio; PCV, a proportional change in variance; UNICEF, United Nations International Children's Emergency Fund; VIF, Variance Inflation Factor; WHO, World Health Organization

Declarations

Consent for publication

Not applicable.

Availability of data and materials

Data are available online in a public, open access repository (www.measuredhs.com/data).

Competing interests

The authors declare that they have no competing interests.

Funding

No funder.

Author Contributions

Conceptualization: DMG. Study design: DMG, KMA, YT, AE, WDN, AMW, LY, MG, NW, AY, LA, HTA, MGT, AH, MJF, and KAD. Execution: DMG, KMA, YT, AE, WDN, AMW, LY, MG, NW, AY, LA, HTA, MGT, AH, MJF, and KAD. Acquisition of the data: DMG, KMA, YT, AE, WDN, AMW, LY, MG, NW, AY, LA, HTA, MGT, AH, MJF, and KAD. Analysis and interpretation: DMG, KMA, YT, AE, WDN, AMW, LY, MG, NW, AY, LA, HTA, MGT, AH, MJF, and KAD. Writing: DMG, KMA, YT, AE, WDN, AMW, LY, MG, NW, AY, LA, HTA, MGT, AH, MJF, and KAD. Review and editing: DMG, KMA, YT, AE, WDN, AMW, LY, MG, NW, AY, LA, HTA, MGT, AH, MJF, and KAD. All authors have read and approved the final version of the manuscript.

Acknowledgments

The authors would like to thank measure DHS for their permission to access the datase

References

1. Organization WH. Diet, nutrition, and the prevention of chronic diseases: report of a joint WHO/FAO expert consultation: World Health Organization; 2003.

2. Lim SS, Vos T, Flaxman AD, Danaei G, Shibuya K, Adair-Rohani H, et al. A comparative risk assessment of burden of disease and injury attributable to 67 risk factors and risk factor clusters in 21 regions, 1990–2010: a systematic analysis for the Global Burden of Disease Study 2010. The lancet. 2012;380(9859):2224-60.

3. Afshin A, Forouzanfar MH, Reitsma MB, Sur P, Estep K, Lee A, et al. Health Effects of Overweight and Obesity in 195 Countries over 25 Years. The New England journal of medicine. 2017;377(1):13-27.

4. Ford ND, Patel SA, Narayan KM. Obesity in Low- and Middle-Income Countries: Burden, Drivers, and Emerging Challenges. Annual review of public health. 2017;38:145-64.

5. Swinburn BA, Sacks G, Hall KD, McPherson K, Finegood DT, Moodie ML, et al. The global obesity pandemic: shaped by global drivers and local environments. The Lancet. 2011;378(9793):804-14.

6. Ng M, Fleming T, Robinson M, Thomson B, Graetz N, Margono C, et al. Global, regional, and national prevalence of overweight and obesity in children and adults during 1980–2013: a systematic analysis for the Global Burden of Disease Study 2013. The lancet. 2014;384(9945):766-81.

7. Chowdhury MAB, Adnan MM, Hassan MZ. Trends, prevalence and risk factors of overweight and obesity among women of reproductive age in Bangladesh: a pooled analysis of five national cross-sectional surveys. BMJ open. 2018;8(7):e018468.

8. Sartorius B, Veerman LJ, Manyema M, Chola L, Hofman K. Determinants of Obesity and Associated Population Attributability, South Africa: Empirical Evidence from a National Panel Survey, 2008-2012. PloS one. 2015;10(6):e0130218.

9. Neupane S, KC P, Doku DT. Overweight and obesity among women: analysis of demographic and health survey data from 32 Sub-Saharan African Countries. BMC public health. 2015;16(1):1-9.

10. Mkuu R, Barry A, Yonga G, Nafukho F, Wernz C, Gilreath T, et al. Prevalence and factors associated with overweight and obesity in Kenya. Preventive medicine reports. 2021;22:101340.

11. Djalalinia S, Yoosefi M, Shahin S, Ghasemi E, Rezaei N, Ahmadi N, et al. The levels of BMI and patterns of obesity and overweight during the COVID-19 pandemic: Experience from the Iran STEPs 2021 survey. Frontiers in Endocrinology. 2022;13:1043894.
12. Mendez MA, Monteiro CA, Popkin BM. Overweight exceeds underweight among women in most developing countries. The American journal of clinical nutrition. 2005;81(3):714-21.
13. Jones-Smith JC, Gordon-Larsen P, Siddiqi A, Popkin BM. Is the burden of overweight shifting to the poor across the globe? Time trends among women in 39 low-and middle-income countries (1991–2008). International journal of obesity. 2012;36(8):1114-20.
14. Manson JE, Willett WC, Stampfer MJ, Colditz GA, Hunter DJ, Hankinson SE, et al. Body weight and mortality among women. New England Journal of Medicine. 1995;333(11):677-85.
15. Cefalu WT, Bray GA, Home PD, Garvey WT, Klein S, Pi-Sunyer FX, et al. Advances in the science, treatment, and prevention of the disease of obesity: reflections from a diabetes care editors' expert forum. Diabetes Care. 2015;38(8):1567-82.
16. Capodaglio P, Liuzzi A. Obesity: a disabling disease or a condition favoring disability? 2013.
17. Doherty DA, Magann E, Francis J, Morrison J, Newnham J. Pre- pregnancy body mass index and pregnancy outcomes. International Journal of Gynecology & Obstetrics. 2006;95(3):242-7.
18. Kulie T, Slattengren A, Redmer J, Counts H, Eglash A, Schrager S. Obesity and women's health: an evidence-based review. The Journal of the American Board of Family Medicine. 2011;24(1):75-85.
19. Leeners B, Rath W, Kuse S, Irawan C, Imthurn B, Neumaier-Wagner P. BMI: new aspects of a classical risk factor for hypertensive disorders in pregnancy. Clinical Science. 2006;111(1):81-6.
20. Bergström A, Hsieh C, Lindblad P, Lu C, Cook N, Wolk A. Obesity and renal cell cancer–a quantitative review. British journal of cancer. 2001;85(7):984-90.
21. Yeh T-L, Hsu H-Y, Tsai M-C, Hsu L-Y, Hwang L-C, Chien K-L. Association between metabolically healthy obesity/overweight and cardiovascular disease risk: a representative cohort study in Taiwan. PloS one. 2021;16(2):e0246378.
22. Raatikainen K, Heiskanen N, Heinonen S. Transition from overweight to obesity worsens pregnancy outcome in a BMI- dependent manner. Obesity. 2006;14(1):165-71.

23. Seligman LC, Duncan BB, Branchtein L, Gaio DSM, Mengue SS, Schmidt MI. Obesity and gestational weight gain: cesarean delivery and labor complications. Revista de saude publica. 2006;40(3):457-65.

24. Jiang K, Chen B, Lou D, Zhang M, Shi Y, Dai W, et al. Systematic review and meta-analysis: association between obesity/overweight and surgical complications in IBD. International Journal of Colorectal Disease. 2022;37(7):1485-96.

25. Kristensen J, Vestergaard M, Wisborg K, Kesmodel U, Secher NJ. Pre-pregnancy weight and the risk of stillbirth and neonatal death BJOG: An International Journal of Obstetrics and Gynaecology 112 (4), 403–408. 2008.

26. Ehrenberg HM, Mercer BM, Catalano PM. The influence of obesity and diabetes on the prevalence of macrosomia. American journal of obstetrics and gynecology. 2004;191(3):964-8.

27. Nucci LB, Schmidt MI, Duncan BB, Fuchs SC, Fleck ET, Britto MMS. Nutritional status of pregnant women: prevalence and associated pregnancy outcomes. Revista de saude publica. 2001;35(6):502-7.

28. Graves BW, DeJoy SA, Heath A, Pekow P. Maternal body mass index, delivery route, and induction of labor in a midwifery caseload. Journal of midwifery & women's health. 2006;51(4):254-9.

29. Marchi J, Berg M, Dencker A, Olander E, Begley C. Risks associated with obesity in pregnancy, for the mother and baby: a systematic review of reviews. Obesity Reviews. 2015;16(8):621-38.

30. Bo S, Menato G, Signorile A, Bardelli C, Lezo A, Gallo M, et al. Obesity or diabetes: what is worse for the mother and for the baby? Diabetes & metabolism. 2003;29(2):175-8.

31. Um S, Yom A, A. Muir J, Sopheab H. Overweight and obesity among women at reproductive age in Cambodia: Data analysis of Cambodia Demographic and Health Survey 2014. PLOS global public health. 2023;3(3):e0001768.

32. Talukder A, Gupta RD, Hashan MR, Haider SS, Sajal IH, Sarker M. Association between television viewing and overweight and obesity among women of reproductive age in Timor-Leste: evidence from the demographic health survey 2016. BMJ open. 2021;11(8):e045547.

33. Seidu A-A, Ahinkorah BO, Agbaglo E, Nyaaba AA. Overweight and obesity among women of reproductive age in Mali: what are the determinants? International Health. 2021;13(5):428-35.

34. Asosega KA, Aidoo EN, Adebanji AO, Owusu-Dabo E. Examining the risk factors for overweight and obesity among women in Ghana: A multilevel perspective. Heliyon. 2023;9(5).

35. Ali NB, Dibley MJ, Islam S, Rahman MM, Raihana S, Bhuiyan SET, et al. Overweight and obesity among urban women with iron deficiency anaemia in Bangladesh. Maternal & Child Nutrition. 2021;17(2):e13102.

36. Mosha D, Paulo HA, Mwanyika-Sando M, Mboya IB, Madzorera I, Leyna GH, et al. Risk factors for overweight and obesity among women of reproductive age in Dar es Salaam, Tanzania. BMC nutrition. 2021;7(1):1-10.

37. Lyrio AO, Souza ES, dos S Conceição S, Batista JE, Brito SM, Gomes Filho IS, et al. Prevalence of overweight and obesity and associated factors among women of childbearing age in Brazil. Public health nutrition. 2021;24(16):5481-90.

38. Mukora-Mutseyekwa F, Zeeb H, Nengomasha L, Kofi Adjei N. Trends in prevalence and related risk factors of overweight and obesity among women of reproductive age in Zimbabwe, 2005–2015. International Journal of Environmental Research and Public Health. 2019;16(15):2758.

39. Kinnunen TI, Bastola K, Neupane S. Trends in the prevalence of overweight and obesity among women of reproductive age. 2021.

40. Shayo GA, Mugusi FM. Prevalence of obesity and associated risk factors among adults in Kinondoni municipal district, Dar es Salaam Tanzania. BMC public health. 2011;11(1):1-7.

41. Yeshaw Y, Kebede SA, Liyew AM, Tesema GA, Agegnehu CD, Teshale AB, et al. Determinants of overweight/obesity among reproductive age group women in Ethiopia: multilevel analysis of Ethiopian demographic and health survey. BMJ open. 2020;10(3):e034963.

42. Gigante DP, Moura ECd, Sardinha LMV. Prevalence of overweight and obesity and associated factors, Brazil, 2006. Revista de saude publica. 2009;43:83-9.

43. Enyew EB, Birhanu AY, Mewosha WZ. Spatial variation of overweight/obesity and associated factor among reproductive age group women in Ethiopia, evidence from EDHS 2016. PloS one. 2022;17(11):e0277955.

44. Kumar P, Mangla S, Kundu S. Inequalities in overweight and obesity among reproductive age group women in India: evidence from National Family Health Survey (2015–16). BMC Women's Health. 2022;22(1):205.

45. Diakité D, Sidibé S, Kolié F, Camara F, Camara DM, Kourouma M, et al. Prevalence and factors associated with the coexistence of overweight/obesity and anaemia among women of reproductive age in Guinea. Public Health Nutrition. 2023:1-10.
46. Worku M, Gizaw Z, Kassahun Belew A, Wagnew A, Hunegnaw MT. Prevalence and associated factors of overweight and obesity among high school adolescents in Bahir Dar City, Northwest, Ethiopia: A cross-sectional study. Journal of Obesity. 2021;2021.
47. Li M, Gong W, Wang S, Li Z. Trends in body mass index, overweight and obesity among adults in the USA, the NHANES from 2003 to 2018: A repeat cross-sectional survey. BMJ open. 2022;12(12):e065425.
48. Omer I, Derese T, Sintayehu Y. Overweight and Its Associated Factors among Women of Reproductive Age in Dire Dawa, Eastern Ethiopia, 2021: Community-Based Cross-Sectional Study. Journal of Obesity. 2022;2022.
49. Larsen TB, Sørensen HT, Gislum M, Johnsen SP. Maternal smoking, obesity, and risk of venous thromboembolism during pregnancy and the puerperium: a population-based nested case-control study. Thrombosis research. 2007;120(4):505-9.
50. Dagne S, Gelaw YA, Abebe Z, Wassie MM. Factors associated with overweight and obesity among adults in northeast Ethiopia: a cross-sectional study. Diabetes, metabolic syndrome and obesity: targets and therapy. 2019;12:391.
51. Monteiro CA. Nutrition and health. The issue is not food, nor nutrients, so much as processing. Public Health Nutrition. 2009;12(5):729-31.
52. Rivera JA, Barquera S, Gonzalez-Cossio T, Olaiz G, Sepulveda J. Nutrition transition in Mexico and in other Latin American countries. Nutrition reviews. 2004;62(suppl_2):S149-S57.
53. Rutstein SO, Rojas G. Guide to DHS statistics. Calverton, MD: ORC Macro. 2006;38.
54. Organization WH. World Health Organization Obesity and Overweight.
55. Chilot D, Belay DG, Ferede TA, Shitu K, Asratie MH, Ambachew S, et al. Pooled prevalence and determinants of antenatal care visits in countries with high maternal mortality: A multi-country analysis. Frontiers in Public Health. 2023;11:1035759.
56. Chilot D, Mondelaers A, Alem AZ, Asres MS, Yimer MA, Toni AT, et al. Pooled prevalence and risk factors of malaria among children aged 6–59 months in 13 sub-Saharan African countries: A multilevel analysis using recent malaria indicator surveys. PloS one. 2023;18(5):e0285265.

57. Malik VS, Willett WC, Hu FB. Global obesity: trends, risk factors and policy implications. Nature reviews endocrinology. 2013;9(1):13-27.

58. Dereje D, Yirgu R, Chichiabellu T. Magnitude of overweight/obesity and associated factors among high school adolescents' in Addis Ababa, Ethiopia. J Nutr Disord Ther. 2018;8(2):2161-0509.

59. Alemu E, Atnafu A, Yitayal M, Yimam K. Prevalence of overweight and/or obesity and associated factors among high school adolescents in Arada Sub city, Addis Ababa, Ethiopia. Journal of nutrition & food sciences. 2014;4(2):1.

60. Delacosta TC. Explorando associações entre sarcopenia, obesidade e osteoporose: estudo com pacientes da atenção primária em saúde. 2019.

61. Santos VRd, Araujo MYC, Cardoso MR, Batista VC, Christofaro DGD, Gobbo LA. Association of insufficient physical activity with sarcopenia and sarcopenic obesity in individuals aged 50 years or more. Revista de Nutrição. 2017;30:175-84.

62. Athukorala C, Rumbold AR, Willson KJ, Crowther CA. The risk of adverse pregnancy outcomes in women who are overweight or obese. BMC pregnancy and childbirth. 2010;10(1):1-8.

63. Holowko N, Mishra G, Koupil I. Social inequality in excessive gestational weight gain. International journal of obesity. 2014;38(1):91-6.

64. Bhurosy T, Jeewon R. Overweight and obesity epidemic in developing countries: a problem with diet, physical activity, or socioeconomic status? The Scientific World Journal. 2014;2014.

65. Monda KL, Adair LS, Zhai F, Popkin BM. Longitudinal relationships between occupational and domestic physical activity patterns and body weight in China. European journal of clinical nutrition. 2008;62(11):1318-25.

66. Sugiyama T, Healy GN, Dunstan DW, Salmon J, Owen N. Is television viewing time a marker of a broader pattern of sedentary behavior? Annals of behavioral Medicine. 2008;35(2):245-50.

67. Abolfotouh MA, Al-Alwan IA, Al-Rowaily MA. Prevalence of metabolic abnormalities and association with obesity among Saudi college students. International journal of hypertension. 2012;2012.

68. Cleland VJ, Schmidt MD, Dwyer T, Venn AJ. Television viewing and abdominal obesity in young adults: is the association mediated by food and beverage consumption during viewing time or reduced leisure-time physical activity? The American journal of clinical nutrition. 2008;87(5):1148-55.

69. Abrha S, Shiferaw S, Ahmed KY. Overweight and obesity and its socio-demographic correlates among urban Ethiopian women: evidence from the 2011 EDHS. BMC Public Health. 2016;16:1-7.

70. Huayanay-Espinoza CA, Quispe R, Poterico JA, Carrillo-Larco RM, Bazo-Alvarez JC, Miranda JJ. Peer Reviewed: Parity and Overweight/Obesity in Peruvian Women. Preventing chronic disease. 2017;14.

71. Pasquali R, Vicennati V, Cacciari M, Pagotto U. The hypothalamic- pituitary- adrenal axis activity in obesity and the metabolic syndrome. Annals of the New York Academy of Sciences. 2006;1083(1):111-28.

72. Yoosefi Lebni J, Mohammadi Gharehghani MA, Soofizad G, Khosravi B, Ziapour A, Irandoost SF. Challenges and opportunities confronting female-headed households in Iran: a qualitative study. BMC women's health. 2020;20:1-11.

73. Navia B, Aparicio A, Perea JM, Pérez-Farinós N, Villar-Villalba C, Labrado E, et al. Sodium intake may promote weight gain; results of the FANPE study in a representative sample of the adult Spanish population. Nutrición hospitalaria. 2014;29(6):1283-9.

74. Darbre PD. Endocrine disruptors and obesity. Current obesity reports. 2017;6:18-27.

Figure Legendes

Figure 1: Prevalence of overweight/obesity of reproductive age women in countries with high maternal mortality

Figure

Click here to access/download;Figure;Figure 1.tif

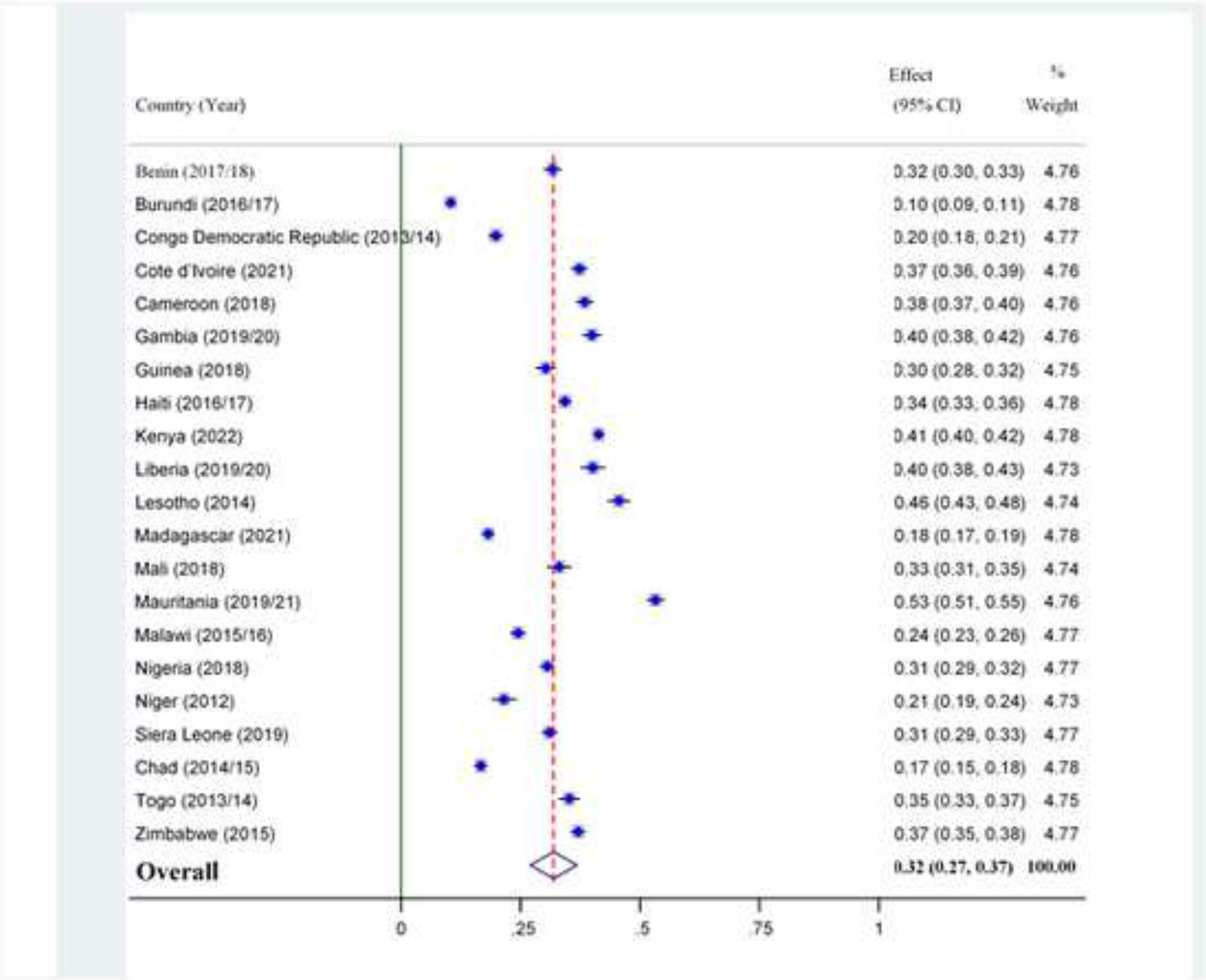

STROBE Checklist

Click here to access/download
Other
Other.docx
