# Supplementary material for: Pooled prevalence and factors of overweight/obesity among women of reproductive age in low and middle-income countries with high maternal mortality: A multi-level analysis of recent demographic and health surveys
Source: PLoS One. 2025 Oct 8;20(10):e0316962. doi: 10.1371/journal.pone.0316962 (PMC12507220; doi:10.1371/journal.pone.0316962)
Supplement: Attachment — Submitted filename: PONE-D-23-42732R1-Review .pdf [file pone.0316962.s004.pdf]

Title: Pooled prevalence and factors of overweight/obesity among reproductive age women in low and middle-income countries with high maternal mortality: A multi-level analysis of recent Demographic and Health Surveys

Dear Editor,

I am writing to express my deep appreciation for the opportunity to evaluate this manuscript. I want to emphasize the invaluable feedback provided by you and the other two reviewers, which has significantly improved the quality of the manuscript. Leveraging my extensive experience with DHS data and methodology, I found the manuscript to be not only lucid but also employing robust statistical approaches. Therefore, after a comprehensive assessment, I confidently recommend the manuscript for publication in its current form.

Abstract: The authors have effectively outlined their research question, identified research gaps, articulated the study's aim, and selected the appropriate statistical approach to address clustering effects. They have also clearly reported the pooled prevalence, significant factors, and provided insightful recommendations.

Background: The authors have acknowledged and addressed comments effectively, demonstrating a thorough understanding of the subject matter.

Methodology: The selection of investigation settings is commendable and will significantly benefit both the scientific community and policymakers. The study's focus on obesity/overweight will provide valuable insights into the gaps in the Millennium Development Goals (MDG) and offer guidance for the ongoing Sustainable Development Goals (SDG). The use of reliable data sources such as the Demographic and Health Surveys (DHS), along with appropriate statistical approaches to account for clustering and valid model comparison methodologies, adds substantial credibility to the study.

Results: The authors provided a clear presentation of socio-demographic results and carefully considered important variables in their descriptive analysis. Additionally, they conducted a meta-analysis to report the pooled prevalence of overweight/obesity, which is a preferable approach compared to reporting individual country proportions.

Discussion: The findings have been articulated with clarity, and I appreciate the thoroughness of the editor's input. The key points for comprehensive discussion have been highlighted. Furthermore, the conclusion is succinct and lucid, and the study's limitations and strengths are distinctly outlined.

Once more, I would like to express my gratitude for the opportunity to review this manuscript. I would like to acknowledge the hard work of the editor, reviewers, and authors in enhancing the clarity of the manuscript.
