# Supplementary material for: Pooled prevalence and factors of overweight/obesity among women of reproductive age in low and middle-income countries with high maternal mortality: A multi-level analysis of recent demographic and health surveys
Source: PLoS One. 2025 Oct 8;20(10):e0316962. doi: 10.1371/journal.pone.0316962 (PMC12507220; doi:10.1371/journal.pone.0316962)
Supplement: Attachment — Submitted filename: PONE-D-23-42732_R1_annotated.pdf [file pone.0316962.s005.pdf]

PLOS ONE

Pooled prevalence and factors of overweight/obesity among reproductive age women in low and middle-income countries with high maternal mortality: A multi-level analysis of recent Demographic and Health Surveys

--Manuscript Draft--

Manuscript Number:	PONE-D-23-42732R1
Article Type:	Research Article
Full Title:	Pooled prevalence and factors of overweight/obesity among reproductive age women in low and middle-income countries with high maternal mortality: A multi-level analysis of recent Demographic and Health Surveys
Short Title:	Pooled prevalence and determinants of overweight/obesity among reproductive age women
Corresponding Author:	Demiss Mulatu Geberu<br>University of Gondar College of Medicine and Health Sciences<br>Gondar, ETHIOPIA
Keywords:	BMI, overweight, obesity, reproductive age women, countries with high maternal mortality
Abstract:	Background: Due to the increased magnitude of overweight/obesity in many countries, the World Health Organization (WHO) has identified it as a significant public health crisis, particularly affecting women of reproductive age in developing nations. Despite obesity/overweight among women of reproductive age being widely acknowledged as a pressing public health issue, there has been limited investigation into its pooled prevalence and various associated factors in low and middle-income countries (LMICs) having high maternal mortality. Thus, the objective of our study was to assess the pooled prevalence and associated factors of overweight/obesity among reproductive-age women in low and middle-income countries with high maternal mortality.<br>Methods: We analyzed secondary data using recent Demographic and Health Survey datasets from 21 low and middle-income countries with high maternal mortality. A weighted sample of 64,076 reproductive-age women was included in the analysis. The variables were extracted from the IR file, and the data were cleaned, recoded, and analyzed using STATA version 14.2 software. A multilevel binary logistic regression model was applied, and adjusted odds ratios (AOR) with a 95% confidence interval and a p-value of ≤ 0.05 were utilized to identify statistically significant associated factors. Model fitness and comparison were assessed using the ICC, MOR, PCV, and deviance (−2LLR).<br>Result: In this study, the pooled prevalence of overweight/obesity among reproductive-age women was 32% (95% CI: 27% - 37%), with a significant variation between countries, ranging from 10% in Burundi to 53% in Mauritania. Reproductive-age women with overweight/obesity showed a significant positive association with various factors compared to those with a normal BMI. These factors include being women aged 25–34 years and 35-49 years, being married, widowed, or divorced, belonging to households with various income levels (poorer, middle, richer, and richest), having different numbers of children (one child, 2-3 children, 4 and above children), watching television, using the internet, and being urban residents.<br>Conclusions and recommendations: More than three out of ten reproductive-age women were overweight/obese in low and middle-income countries with high maternal mortality. Individual-level and community-level factors were associated with overweight/obesity. Special attention is recommended to older women, those with formal education, non-working women, individuals who spend time to watch television and using the internet, urban residents, female household heads, and women in the wealthiest households.
Order of Authors:	Demiss Mulatu Geberu
	Kaleab Mesfin Abera
	Yawkal Tsega
	Abel Endawkie

Powered by Editorial Manager® and ProduXion Manager® from Aries Systems Corporation

	Wubshet Debebe Negash
	Amare Mesfin Workie
	Lamrot Yohannes
	Mihret Getnet
	Nigusu Worku
	Adina Yeshambel Belay
	Lakew Asmare
	Hiwot Tadesse Alemu
	Misganaw Guadie Tiruneh
	Asebe Hagos
	Melak Jejaw
	Kaleb Assegid Demissie
**Opposed Reviewers:**	

Response to Reviewers:

Point-by-point response for editor’s and reviewers' comments
Editor’s and Reviewers’ comments and suggestionsAuthors' responses
Editor’s comments and suggestions
1.Thank you for submitting your manuscript to PLOS ONE. After careful consideration, we feel that it has merit but does not fully meet PLOS ONE’s publication criteria as it currently stands. Therefore, we invite you to submit a revised version of the manuscript that addresses the points raised during the review process.Dear Editor, thank you for your consideration and suggestion! We revised the manuscript according to your comments and suggestions.
2.I have extensibly annotated the original comments and provided feedback about changes to be done in the manuscript in each section. My main concern is about pooling data. First, DHSs data use a two-stage sampling design. When data are pooled I wonder which weights have been used to estimates coefficients (AOR)?Dear Editor, thank you for your concern and comment. Pooled data analysis is a methodology of analyzing data from different sources which has the advantage of producing more accurate estimation and greater statistical power. Besides, it helps to generalize findings from different populations. Therefore, by considering the above importance, we conducted pooled data analysis to determine the magnitude of overweight/obesity only. However, we used normal (non-pooled) overweight/obesity categorized (Yes/No) data to conduct multilevel multivariable regression analysis. Hence, we used normal (non-pooled) data to identify the associated factors of the dependent variable and their statistical parameters/coefficients/ such as AOR, COR, P-value, and 95% CI.
Dear editor, as we have tried to describe in the above sentences as to our knowledge and understanding we gave more value for pooled data rather than non-pooled data due to its strong nature of analysis and evidence. In this case, we kindly hope you can understand our intention and this description will answer your comments of pooled data analysis which were raised throughout the document.
3.Second, the outcome variable is not well operationalized (see manuscript for comments). Are underweight women excluded from analyses. This is unclear from the manuscript. It that is the case, then this introduces additional statistical concern because when estimates are done on sub-populations, additional care should be done to ensure that representativeness of samples are preserved.Dear editor, thank you for your comment, for the purpose of further clarity, we have revised and merged the “variable definition” and “operational definition” sections and labeled as “variables and measurements”. Besides, your raised concerns are addressed as per your suggestion, please see the clean manuscript on pages 8-10, lines 169 -210.
Regarding the inclusion/exclusion of underweight women: As we know, our analysis should be done based on our title/topic. Due to this reason, we didn’t include underweight (mildly, moderately and severely thin) women both in the numerator and in the denominator because we are focused to study about the magnitude and associated factors of overweight/obesity as mentioned in the topic. The dependent variable was fixed based on our previous practice in related article [1], other previous published literature [2], and WHO classification [3]. Therefore, as to our knowledge and

	the practices, there will not be a statistical concern because the dependent variable was fixed by strictly following the standard DHS guide and previous articles.<br>4.Third, the discussion section is very flawed since it is like a "redo" of results section. This section should have been more clearer and useful if the authors engaged discussing factors associated with overweight/obesity at (i) individual level; and (ii) community level. The authors should really engage in key findings and how they align or differ from what is already known on the subject;Dear editor, thank you for your comment and concern, we revised the discussion according to your suggestion by considering the addressable issues, please see the revised section of the discussion.<br>5.Fourth, authors are referring to high maternal mortality countries without any references on the adopted classification.Dear editor, thank you very much for your constructive comment, we have mentioned the source of this classification and we also included in the variables and measurements section for further understanding. We hope this definition will answer all comments regarding high maternal mortality, please see the revised clean manuscript on page 10, lines 208-210 and on page 8, line 166.<br>6.Fifth, the manuscript requires serious edits. Dear editor, thank you for your constructive comments, we have edited and revised each section of the manuscript which needs edition.<br>7.Why should countries be interested in pooled prevalence? Pragmatically and policy-wise, this is less relevant!Dear editor, thank you for your comment! All the included countries share the same source of information (DHS survey) and for this reason this pooled analysis will have pragmatic policy implications. Thus, to get holistic picture about overweight/obesity among reproductive age women in countries with high maternal mortality need to be evaluated empirically for up-to-date intervention by respective country governments and other partners working on maternal health.<br>8.Clarify! What is high maternal mortality?Thank you for your question, we have clarified it accordingly. Maternal mortality is considered to be high if it is 300-499, very high if it is 500-999, and extremely high if it is equal to and higher than 1000 maternal deaths per 100,000 live births. For the sake of maintaining economy of words and attractiveness in the abstract we have clarified it in the variables and measurements section, please see the updated clean manuscript on page 10, lines 208-210.<br>9.DHSs don't allow causality; therefore determinant is inappropriateDear editor, thank you for your suggestion, we have revised the whole manuscript which states about determinants according to your valuable suggestion, please see the updated manuscript.<br>10.This is fuzzy in an abstract. Not easy to get a sense of the message conveyed in this long sentenceDear editor, thank you for your constructive comment, for the sake of conciseness in the abstract we tried to shorten it by mentioning factors having more strong association. Please see the revised section of the abstract on page 3, lines 65 to 70. Our assumption when we omit these less strong factors is that readers can get the detail of all factors on the result and discussion section.<br>11.General comment: The English is poor and needs serious improvement!Dear editor, thank you for your comment, we have revised and edited the entire manuscript by language expert to improve the language of the manuscript.<br>Besides, we revised the background section according to your specific comments and tried to include these specific comments' response in this point-by-point response.<br>12.This kind of construction breaks the flow of the paper. Talk first of prevalence from previous studies globally, in LMICs, and at country-level. Thereafter, move to consequences, and associated factors.Dear editor, thank you for your insights and improvement suggestion, we revised according to your suggestion, please see the revised clean manuscript on page 5-6, lines 94-120.<br>13.It is not watching TV per se. It is about "sedentary behaviour"Dear editor, you are correct, media usage in general is related with sedentary behavior which in return affects overweight and we have clearly stated on the possible justification of the discussion section. However, in the background section, for the purpose of specificity we have presented as frequency of watching television.<br>14.what's the added value of pooled prevalenceDear editor, thank you for your clarification question! Pooled data analysis is a methodology of analyzing data from different sources which has the advantage of producing more accurate estimation and greater statistical power. Besides, it helps to generalize findings from different populations. Therefore, by considering the above importance, we conducted pooled data analysis to determine the magnitude.<br>15.Readers were expecting till now to know (understand) how high maternal mortality was defined. Nothing but speculationsDear editor, thank you for your comment again,

	we defined in the “variables and measurement” section.<br>16.When data are pooled, how did the authors incorporate the complex survey design (CSD) in DHSs?Dear editor, thank you for your question, as mentioned in the method part of the manuscript, cross-sectional study design was used in all Demographic and Health Surveys. Therefore, there will no be a problem of complex survey design.<br>17.This is what? It is well known that DHSs collect information on women of reproductive ages (15-49 years). What this sentence adds up?Dear editor, yes you are correct it didn’t have value and thank you for your valuable comment, we removed it.<br>18.Data sources for these statistics ?Dear editor, thank you for your question, we mentioned the data source, please see at the bottom of table 1. We have used three data sources as follows: http://www.dhsprogram.com, http://srhr.org/mmr2020, or http://www.who.int/publications/i/item/9789240068759<br>19.Whose classification is it?Dear editor, thank you for your clarification question, this is the classification of WHO, UNICEF, and world bank. We have cited the appropriate reference, please see the revised clean manuscript on page 8, line 166.<br>20.This is vague while this key for the manuscript. How was it collected in DHSs? How was it treated in this paper? How the decision to operationalize the original variable was taken ?Dear editor, thank you for your valuable comments and insights, we revised it according to your comment, please see the “variable and measurement” section on pages 8-9, lines 171-185.<br>21.This should go to "outcome variable?"Dear editor, thank you for your valuable suggestion, we revised it accordingly, please see on pages 8-9, lines 176-179.<br>22.why capitalize?Thank you for your question! We had a practice of capitalizing the first spelling of abbreviated phrases and that is why we capitalized the first spellings.<br>23.This is debatable since even non-bivariate associations could become "significant" in multivariable analyses. this choice should be justifiedDear editor, thank you for your comment! Yes, it is debatable, however, as the practice of different scholars we used 0.25 as a criterion to filter our candidate variables for multilevel regression analysis.<br>24.This section is too log for secondary data analysisDear editor, yes you are correct, it is long. However, we did this to answer the questions which were raised by the PLOS ONE journal assistants during technical check as journal requirement.<br>25.For binary variables and to make Table clearer, knowing "one category" reveals info on the other category.Dear editor, thank you for your comment and you are correct.<br>26.I wonder whether these 2 sub-headings are necessary (Random vs. Fixed effects)Dear editor, thank you for your suggestion, we corrected is as per your suggestion, please see the revised manuscript on page 15.<br>27.This is not the numbers of women of reproductive ages in DHS report? How do we trust these numbers ?Dear editor, thank you for your clarification question, this is the number of non-pregnant, who didn’t have births in the last five years, and non-underweight women which was fixed according to the DHS statistics guide. Additionally, we reached to this number after omitting all missing variables, so it might not be equal to the observed reproductive age women. For further understanding please see the flow chart named as figure 1 and attached as TIF file, please see the uploaded figure.<br>28.It should have been a good idea to split discussion into 2 sub-headings: Individual-level factors and community-level factors and better engage the discussion and provide plausible explanations to understand findingsDear editor, we understand your concerns on the discussion and appreciate your specific comments in the manuscript. However, we face difficulties to split the discussion in to two sub-headings. Because if we split in to two sub-headings there will be redundancy of possible justifications between some individual level and community level factors which might affect the attractiveness of this section. For example; frequency of watching television from individual level factors and community-level media usage from community level factor had association with the dependent variable. In these comparable variables we had similar possible justifications. If we split these comparable variables in to different sections and if we state the similar possible justification, repetition of idea might occur.<br>29.This is a less productive way to engage in a discussion, which shouldn't be a "redo" of resultsDear editor, thank you for your comment, we presented the factors that need modification in synthesized manner, please see on page 19, lines 299-305.<br>30.what's functional performance?Dear editor, thank you for your clarification point, we revised the full sentence to render full meaning, please see the clean manuscript on page 19, lines 310-311.<br>31.This should be dug enoughDear editor, thank you for your critical insight, we revised it in detail, please see the clean manuscript on page 20, lines 319-330.

Powered by Editorial Manager® and ProduXion Manager® from Aries Systems Corporation

	Reviewer 1: comments and suggestions<br>1.The manuscript is good in terms of objective but not an easy<br>article to comprehend. The writing should be improved. For example in the abstract the authors written "Women aged 25–34 years and 35-49 years, women who had primary education, who had<br>secondary education and above, not-working women, married, widowed, and divorced women,<br>poorer, middle, richer, and richest household, female household head, having one child, 2-3<br>children, 4 and above children, watching television less than once a week and at least once a week,<br>using internet less than once a week and at least once a week, improved source of drinking water,<br>urban residents, low community poverty, and high community level media usage." -- it is not a complete sentence.Dear reviewer, thank you very much for your valuable comment, we modified this sentence, please see the abstract on page 3, lines 65-70.<br>2.I attempted to find the country-specific prevalence of obesity but was unsuccessful in the manuscript. It will be helpful to know the country-specific prevalence before pooling the results.Dear reviewer, thank you for your comment, the country specific prevalence was done and stated on figure 2 which was uploaded as TIF file separately, please see the uploaded figure.<br>3.I recommend that the authors incorporate a discussion of their results in the context of previous studies.Dear reviewer, thank you in advance for your recommendation, we have discussed the magnitude from our result and the associated factors from previous studies. We have discussed the magnitude from our result purposely because our study is about pooled prevalence and we didn’t found pooled prevalence of overweight/obesity form previous studies. In this case if we compare our pooled prevalence from previous single prevalence studies, this will lead us to compare incomparable magnitudes/findings. This is the reason why we had discussed the magnitude by using our result only. However, we have discussed the associated factors from previous studies in detail which is inline with your valuable recommendation.<br>Reviewer 2: comments and suggestions<br>1.1If the initially considered countries were low- and middle-income countries, modify the title to include "high maternal mortality in low and middle-income countries."Dear reviewer, thank you very much for your insight, we revised the title according to your constructive comment. Besides, the rest of the manuscript is also modified according to the slight modification in the title.<br>2.2A flowchart illustrating the number of countries initially considered, and how 21 countries with a sample size of 64,076 were reached, will make the sampling process easier to understand.Dear reviewer, thank you very much for your important suggestion, we prepared the flowchart according to your valuable suggestion which was uploaded as TIF file separately, please see the uploaded figure 1.<br>3.I also suggest that the outcome variable be overweight/obesity and dichotomized as yes/no.Dear reviewer, thank you in advance again for your valuable suggestion. We revised the manuscript according to your valuable suggestion, please see on page 9, lines 179-181.<br><br>References for this authors’ response<br><br>1.Getnet M, Sisay WS, Alem AZ: Spatial distribution and associated factors of co-occurrence of overweight/obesity and Anemia among women in the reproductive age in sub-Saharan Africa: A multilevel analysis, DHS 2016–2021. PloS one 2024, 19(4):e0299519.<br>2.Yeshaw Y, Kebede SA, Liyew AM, Tesema GA, Agegnehu CD, Teshale AB, Alem AZ: Determinants of overweight/obesity among reproductive age group women in Ethiopia: multilevel analysis of Ethiopian demographic and health survey. BMJ open 2020, 10(3):e034963.<br>3.Organization WH: World Health Organization Obesity and Overweight. In.
**Additional Information:**	
**Question**	**Response**
**Financial Disclosure**	The author(s) received no specific funding for this work.

Enter a financial disclosure statement that describes the sources of funding for the work included in this submission. Review the submission guidelines for detailed requirements. View published research articles from *PLOS ONE* for specific examples.<br><br>This statement is required for submission and **will appear in the published article** if the submission is accepted. Please make sure it is accurate.<br><br>**Funded studies**<br>Enter a statement with the following details:<br>• Initials of the authors who received each award<br>• Grant numbers awarded to each author<br>• The full name of each funder<br>• URL of each funder website<br>• Did the sponsors or funders play any role in the study design, data collection and analysis, decision to publish, or preparation of the manuscript?<br><br>Did you receive funding for this work?	
**Competing Interests**<br><br>Use the instructions below to enter a competing interest statement for this submission. On behalf of all authors, disclose any competing interests that could be perceived to bias this work—acknowledging all financial support and any other relevant financial or non-financial competing interests.<br><br>This statement is required for submission and **will appear in the published article** if the submission is accepted. Please make sure it is accurate and that any funding sources listed in your Funding Information later in the submission form are also declared in your Financial Disclosure statement.	The authors have declared that no competing interests exist

View published research articles from *PLOS ONE* for specific examples.<br><br>**NO authors have competing interests**<br><br>Enter: *The authors have declared that no competing interests exist.*<br><br>**Authors with competing interests**<br><br>Enter competing interest details beginning with this statement:<br><br>*I have read the journal's policy and the authors of this manuscript have the following competing interests: [insert competing interests here]*<br><br>* typeset	
**Ethics Statement**<br><br>Enter an ethics statement for this submission. This statement is required if the study involved:<br><br>• Human participants<br>• Human specimens or tissue<br>• Vertebrate animals or cephalopods<br>• Vertebrate embryos or tissues<br>• Field research<br><br>Write "N/A" if the submission does not require an ethics statement.<br><br>General guidance is provided below. Consult the submission guidelines for detailed instructions. **Make sure that all information entered here is included in the Methods section of the manuscript.**	We have obtained permission from the DHS program to access the data online at http://www.dhsprogram.com and to download and use the data freely. The original collection of DHS data adhered to international and national ethical guidelines, with ethical clearance approved by the ICF Macro Institutional Review Board, the Centers for Disease Control and Prevention (CDC), and the Institutional Review Board (IRB) in each country, in accordance with the United States Department of Health and Human Services requirements for human subject protection. Written consent was obtained from all respondents and/or their legal guardians for minors (aged below 16) to participate, in line with Helsinki declarations. Importantly, the procedures for DHS public-use datasets ensure that respondents, households, or sample communities cannot be identified in any way, as the data files do not contain personal identifiers of individuals or household addresses. Furthermore, this study is not an experimental study. For more details on the ethical standards and data usage by the DHS, please refer to: http://goo.gl/ny8T6X

**Format for specific study types**<br><br>**Human Subject Research (involving human participants and/or tissue)**<br>• Give the name of the institutional review board or ethics committee that approved the study<br>• Include the approval number and/or a statement indicating approval of this research<br>• Indicate the form of consent obtained (written/oral) or the reason that consent was not obtained (e.g. the data were analyzed anonymously)<br><br>**Animal Research (involving vertebrate animals, embryos or tissues)**<br>• Provide the name of the Institutional Animal Care and Use Committee (IACUC) or other relevant ethics board that reviewed the study protocol, and indicate whether they approved this research or granted a formal waiver of ethical approval<br>• Include an approval number if one was obtained<br>• If the study involved *non-human primates*, add *additional details* about animal welfare and steps taken to ameliorate suffering<br>• If anesthesia, euthanasia, or any kind of animal sacrifice is part of the study, include briefly which substances and/or methods were applied<br><br>**Field Research**<br><br>Include the following details if this study involves the collection of plant, animal, or other materials from a natural setting:<br>• Field permit number<br>• Name of the institution or relevant body that granted permission	
**Data Availability**<br><br>Authors are required to make all data underlying the findings described fully available, without restriction, and from the time of publication. PLOS allows rare exceptions to address legal and ethical concerns. See the PLOS Data Policy and FAQ for detailed information.	Yes - all data are fully available without restriction

A Data Availability Statement describing where the data can be found is required at submission. Your answers to this question constitute the Data Availability Statement and **will be published in the article**, if accepted.<br><br>**Important:** Stating 'data available on request from the author' is not sufficient. If your data are only available upon request, select 'No' for the first question and explain your exceptional situation in the text box.<br><br>Do the authors confirm that all data underlying the findings described in their manuscript are fully available without restriction?	
**Describe where the data may be found in full sentences. If you are copying our sample text, replace any instances of XXX with the appropriate details.**<br><br>• If the data are **held or will be held in a public repository**, include URLs, accession numbers or DOIs. If this information will only be available after acceptance, indicate this by ticking the box below. For example: *All XXX files are available from the XXX database (accession number(s) XXX, XXX.)*.<br>• If the data are all contained **within the manuscript and/or Supporting Information files**, enter the following: *All relevant data are within the manuscript and its Supporting Information files.*<br>• If neither of these applies but you are able to provide **details of access elsewhere**, with or without limitations, please do so. For example:<br><br>*Data cannot be shared publicly because of [XXX]. Data are available from the XXX Institutional Data Access / Ethics Committee (contact via XXX) for researchers who meet the criteria for access to confidential data.*<br><br>*The data underlying the results presented in the study are available from (include the name of the third party*	The data used for the analysis of this study is attached as supplementary file 1.

and contact information or URL).<br>• This text is appropriate if the data are owned by a third party and authors do not have permission to share the data.<br><br>* typeset	
Additional data availability information:	

To: **PLOS ONE Editorial office**

Subject: Submission of a revised manuscript for publication

Dear: PLOS ONE editorial office,

We are happy to submit our revised manuscript entitled **"Pooled prevalence and factors of overweight/obesity among reproductive age women in low and middle-income countries with high maternal mortality: A multi-level analysis of recent Demographic and Health Surveys"** to your esteemed journal for publication consideration. We certify that we have participated sufficiently in the intellectual content, conception, analysis, and interpretation of the data, as well as the writing of the manuscript to take public responsibility for it and have agreed to have our name listed as a contributor. Moreover, all authors gave the right to the corresponding author to make necessary changes as per the request of the journal, do the rest of the correspondence, and I will act as the guarantor for the manuscript on our behalf. Furthermore, we ensure that this is our original work and we also declare that we do not have any competing interest. Hence, it is to request you for considering it in your journal for publication.

Thank you in advance!

Demiss Mulatu Geberu

mulatu.demissma23@gmail.com

Corresponding author

Manuscript Click here to access/download;Manuscript;Manuscript.docx

Pooled prevalence and factors of overweight/obesity among reproductive age women in low and middle-income countries with high maternal mortality: A multi-level analysis of recent Demographic and Health Surveys

Demiss Mulatu Geberu*[1], Kaleab Mesfin Abera[2], Yawkal Tsega[3], Abel Endawkie[4], Wubshet Debebe Negash[1], Amare Mesfin Workie[5], Lamrot Yohannes[6], Mihret Getnet[7,8], Nigusu Worku[1], Adina Yeshambel Belay[1], Lakew Asmare[4], Hiwot Tadesse Alemu[1], Misganaw Guadie Tiruneh[1], Asebe Hagos[1], Melak Jejaw[1], Kaleb Assegid Demissie[1]

[1]Department of Health Systems and Policy, Institute of Public Health, College of Medicine and Health Sciences, University of Gondar, Gondar, Ethiopia.

[2]Department of Health Systems and Policy, Institute of Public Health, College of Medicine and Health Sciences, Wollo University, Dessie, Ethiopia.

[3]Department of Health System and Management, School of Public Health, College of Medicine and Health Sciences, Wollo University, Dessie, Ethiopia.

[4]Department of Epidemiology and Biostatistics, School of Public Health, College of Medicine and Health Sciences, Wollo University, Dessie, Ethiopia.

[5]Departement of Nutrition, Institute of Public Health, College of Medicine and Health Sciences, University of Gondar, Gondar, Ethiopia.

[6]Department of Environmental and Occupational Health and Safety, Institute of Public Health, University of Gondar, Gondar, Ethiopia.

[7]Department of Human Physiology, School of Medicine, College of Medicine and Health Sciences, University of Gondar, Gondar, Ethiopia

[8]Department of Epidemiology and biostatistics, Institute of public Health, College of Medicine and Health Sciences, University of Gondar, Gondar, Ethiopia

Email addresses:

DMG: mulatu.demissma23@gmail.com

KMA: kaleabmesfin801@gmail.com

YT: yawkaltsega@gmail.com

AE: abelendawkie@gmail.com

WDN: wubshetdn@gmail.com

AMW: amare7595@gmail.com

LY: lamrotyohannes@gmail.com

MG: mihretgetnet1@gmail.com

NW: nigusuworku29@gmail.com

AYB: adinayeshambel2012@gmail.com

LA: lakewt071981@gmail.com

HTA: tadessehalemu@gmail.com

MGT: mguadie41@gmail.com

AH: asebehagos21@gmail.com

MJ: melak4903@gmail.com

KAD: kalebassegid2019@gmail.com

***Correspondence:**

Demiss Mulatu Geberu

Email : mulatu.demissma23@gmail.com

P.O. Box: 196, Gondar, Ethiopia.

ABSTRACT

Background: Due to the increased magnitude of overweight/obesity in many countries, the World Health Organization (WHO) has identified it as a significant public health crisis, particularly affecting women of reproductive age in developing nations. Despite obesity/overweight among women of reproductive age being widely acknowledged as a pressing public health issue, there has been limited investigation into its pooled prevalence and various associated factors in low and middle-income countries (LMICs) having high maternal mortality. Thus, the objective of our study was to assess the pooled prevalence and associated factors of overweight/obesity among reproductive-age women in low and middle-income countries with high maternal mortality.

Methods: We analyzed secondary data using recent Demographic and Health Survey datasets from 21 low and middle-income countries with high maternal mortality. A weighted sample of 64,076 reproductive-age women was included in the analysis. The variables were extracted from the IR file, and the data were cleaned, recoded, and analyzed using STATA version 14.2 software. A multilevel binary logistic regression model was applied, and adjusted odds ratios (AOR) with a 95% confidence interval and a p-value of ≤ 0.05 were utilized to identify statistically significant associated factors. Model fitness and comparison were assessed using the ICC, MOR, PCV, and deviance (−2LLR).

Result: In this study, the pooled prevalence of overweight/obesity among reproductive-age women was 32% (95% CI: 27% - 37%), with a significant variation between countries, ranging from 10% in Burundi to 53% in Mauritania. Reproductive-age women with overweight/obesity showed a significant positive association with various factors compared to those with a normal BMI. These factors include being women aged 25–34 years and 35-49 years, being married, widowed, or divorced, belonging to households with various income levels (poorer, middle, richer, and richest), having different numbers of children (one child, 2-3 children, 4 and above children), watching television, using the internet, and being urban residents.

Conclusions and recommendations: More than three out of ten reproductive-age women were overweight/obese in low and middle-income countries with high maternal mortality. Individual-level and community-level factors were associated with overweight/obesity. Special attention is recommended to older women, those with formal education, non-working women, individuals who

spend time to watch television and using the internet, urban residents, female household heads, and women in the wealthiest households.

Keywords: BMI, overweight, obesity, reproductive age women, countries with high maternal mortality

Background

The global prevalence of overweight and obesity is a growing public health concern, characterized by the WHO as a pandemic due to its impact in both developed and developing countries (1). It ranks as the sixth-highest cause of disability-adjusted life years (DALYs) and leads to approximately 4.0 million annual deaths worldwide (2-4). Additionally, there has been a 28.3% increase in global mortality related to overweight/obesity from 1990 to 2015 (3). Studies have indicated that marked dietary changes have resulted in a faster increase in the problem in LMICs compared to higher-income countries (4, 5). Out of the two billion overweight/obese individuals globally, 62% were identified in LMICs (6).

Multiple studies have indicated a significantly higher prevalence of the condition in women as compared to men (3-5, 7-11). Despite LMICs being affected by both overweight/obesity and underweight issues, the prevalence of overweight surpasses that of underweight among adult women (12). A trend analysis conducted on reproductive-age women of 39 LMICs indicated a rising prevalence of overweight/obesity over time in the lowest economic groups compared to the highest economic groups (13).

Several studies have shown high rates of overweight and obesity among women of reproductive age in various countries. The prevalence was reported at 55.2% in Brazil (14), 43% in Bangladesh (15), 18% in Cambodia (16), 19.4% in Timor-Leste (17), 35.5% in Ghana (18), 50.4% in Tanzania (19), and 26.9% in Mali (20). Despite interventions such as restrictions on unhealthy food advertising, improvements in school meals, taxation to reduce consumption of unhealthy foods, and supply chain incentives to promote healthy foods, the prevalence of overweight/obesity in reproductive-age women continues to rise unacceptably (3). Trend analyses have shown substantial increases over time, with rates in Bangladesh increased from 9.35% in 1999 to 39.14% in 2014 (7), in Zimbabwe from 25% in 2005 to 36.6% in 2015 (21), and Nepal from 20.5% in 2006 to 41.1% in 2016 (22).

Previous studies found that overweight/obesity was directly contributed to 53% of all female deaths, serving as a well-documented risk factor for obstetric complications in both reproductive-age women and their future offspring (23). In women of reproductive age, complications can include diabetes mellitus (8, 24-27), hypertension (26-28), Chronic Kidney Disease (CKD) (3, 29),

Congestive Heart Failure (CHF) (3, 30), cesarean delivery and surgical site infection (26, 27, 31-33), miscarriage and stillbirth (34), fetal macrosomia (35, 36), breast and cervical cancers (27), postpartum endometritis (37), prolonged hospital stay, and postpartum hemorrhage (26, 31). For newborns, complications can include low birth weight, congenital malformations, preterm birth, large-for-gestational-age babies, and perinatal death are some of the complications (27, 38, 39).

Various studies have identified multiple socioeconomic and behavioral factors contributing to overweight and obesity among reproductive-age women. These factors include high socioeconomic status (1, 8, 10, 15, 18, 20-22, 27, 40-45), age (1, 9, 10, 14, 15, 19-22, 27, 40, 41, 43), parity (14, 22), marital status (8, 10, 14, 16, 19-21), education (10, 16, 18, 20, 22, 40, 41, 46, 47), occupation status (46), urban residence (1, 8, 10, 16, 21, 41, 45), number of household members (7), contraceptive use (40, 48), number of children (16, 45), and frequency of watching television (17, 20). Additionally, behavioral factors such as alcohol consumption, smoking, and dietary habits play a significant role (49, 50).

The rise in overweight/obesity can be attributed to nutritional transition from traditional whole-food-based meals composed of foods like grains that have low animal source foods, salt, refined oils and sugars (51) to energy dense and nutrient-poor diets composed of refined carbohydrates, high fat intake, and processed foods (52). Studies have shown that frequent consumption of sweets, meat, and eggs, as well as snacking, contribute to increased overweight/obesity in women of reproductive age (1, 8, 21). Moreover, lower levels of physical activity and certain ethnicities have been identified as determinants of overweight/obesity (8, 19, 27, 47).

It is widely acknowledged that obesity and overweight among women of reproductive age pose a significant public health concern. However, there has been limited investigation into the pooled prevalence and contributing factors in low and middle-income countries having high maternal mortality using recent DHS data. Timely and comprehensive information on the rapid progression and health impacts of obesity and overweight is crucial for the development of effective prevention policies. To address the mortality and morbidity associated with overweight/obesity in women of reproductive age, evidence-based interventions should be identified and implemented by healthcare providers and policy-makers. Educating women and communities about potential risk factors and promoting healthy behaviors is also essential. Therefore, our study aimed to provide reliable evidence to support the WHO's goal of halting the rise in obesity by 2025 by assessing the

prevalence and associated factors of overweight/obesity among women of reproductive age in low and middle-income countries with high maternal mortality.

Materials and methods

Study design, setting, and period

The Demographic and Health Surveys (DHS) are comprehensive cross-sectional surveys that gather data to provide population and health indicators at both the national and sub-national levels. For this study, we specifically focused on low and middle-income countries having high maternal mortality and available DHS data, including Benin, Burundi, Chad, Democratic Republic of the Congo (DRC), Cote d'Ivoire, Cameroon, Gambia, Guinea, Haiti, Kenya, Liberia, Lesotho, Madagascar, Mali, Mauritania, Malawi, Nigeria, Niger, Sierra Leone, Togo, and Zimbabwe during the period from 2012 to 2022.

Data source and population

We compiled data from Demographic and Health Surveys (DHSs) conducted in twenty-one low and middle-income countries that have high maternal mortality (Table 1). Among these countries, four do not have any available DHS reports, and one does not have publicly accessible DHS data. Additionally, one country does not have data on Body Mass Index (BMI), and another country does not have appropriate BMI data. Conducting a multi-country analysis using DHS survey data is feasible and reliable, as the surveys utilize consistent questionnaires, sampling procedures, data collection methods, and coding (53).

Our study encompassed all women of reproductive age (15-49 years) residing in low and middle-income countries with high maternal mortality who had valid BMI measurements and were alive at the time of the survey. For this analysis, we utilized the individual record (IR) file, encompassing all women of reproductive age at the time of the survey, excluding those who were pregnant, had given birth in the last five years, or had inappropriate BMI measurements based on DHS guidance. We adjusted for over and under-sampling using the sample weight (v005/1,000,000) to ensure the generalizability of the findings. Consequently, the total weighted sample size from the combined data was 64,076 (Figure 1).

Table1: Maternal mortality category and year of the survey by the country

Sr.No	Country	Year of DHS Survey	Maternal mortality/100,000	Category
1.	Benin	2017-18	523	Very High
2.	Burundi	2016-17	494	High
3.	Chad	2014-15	1063	Extremely High
4.	Democratic Republic of the Congo (DRC)	2013-14	547	Very High
5.	Cote d'Ivoire	2021	480	High
6.	Cameroon	2018	438	High
7.	Gambia	2019-20	458	High
8.	Guinea	2018	553	Very High
9.	Haiti	2016-17	350	High
10.	Kenya	2022	530	Very High
11.	Liberia	2019-20	652	Very High
12.	Lesotho	2014	566	Very High
13.	Madagascar	2021	392	High
14.	Mali	2018	440	High
15.	Mauritania	2019-21	464	High
16.	Malawi	2015-16	381	High
17.	Nigeria	2018	1047	Extremely high
18.	Niger	2012	441	High
19.	Siera Leone	2019	443	High
20.	Togo	2013-14	399	High
21.	Zimbabwe	2015	357	High

High:300-499; Very High:500-999; Extremely High:>1000 (54)

Data sources: http://www.dhsprogram.com, http://srhr.org/mmr2020, or http://www.who.int/publications/i/item/9789240068759

Variables and Measurements

Outcome variable:

BMI was the primary variable of interest and determined by dividing weight in kilograms by the square of height in meters (55). The DHS findings uncovered that measurements were conducted on women of reproductive age (15-49 years old) in all selected households. Weight measurements were taken using portable SECA mother-infant scales with a digital display, which were developed and produced with close oversight from UNICEF. Height measurements were obtained using a Shorr measuring board. We categorized and standardized the BMI (dependent variable) into normal and overweight/obesity as per the WHO classification (56) and the guide to DHS statistics,

DHS-7 (55). Accordingly, a normal BMI is within the range of 18.5 to 24.9 kg/m^2, while a BMI ≥25 kg/m^2 indicates overweight, and those with a BMI ≥30 kg/m^2 were classified as obese. For analytical purposes, reproductive age women who were overweight or obese were categorized as "yes" and those with a normal BMI were categorized as "no". We excluded underweight (mildly, moderately, and severely thin) women from both the numerator and denominator, as our focus was specifically on exploring the prevalence and associated factors of overweight/obesity as outlined in the study's topic. The decision to exclude underweight women from this analysis is in line with findings from previously published articles (41, 57).

Independent variables:

In our analysis, the independent variables consist of individual-level factors such as the age, educational status, occupation, and marital status of women, as well as the number of household members, wealth index, use of contraceptive methods, smoking habits, sex of the household head, number of living children, and access to media sources like television, newspapers/magazines, radio, and the internet.

Additionally, community-level factors including place of residence, distance to health facilities, community poverty level, community women's education, and community media usage were also considered. The last three community-level variables were not directly available in the DHS dataset, so we derived them by aggregating the selected individual and household level factors at the cluster level and categorized them as higher or lower based on a median value.

Community-level women's education refers to the percentage of reproductive-age women in the community who have attained primary education or higher. This measure is categorized as low if less than 50% of women have primary education or higher, and high if more than 50% of women have attained this level of education (58, 59).

Community poverty was categorized as low if less than 50% of women in the community fell into low wealth index categories such as poorest and poorer, and high if 50% or more fell into these categories (58, 59).

Community-level media usage refers to the proportion of reproductive age women in the community who watched television, listened radio, read newspapers/magazines, and used internet.

It was considered low if less than 50% of respondents had media exposure, and high if 50% or more had media exposure (58, 59).

The maternal mortality ratio (MMR) is categorized as high if it falls within the range of 300-499, very high if it is between 500-999, and extremely high if it equals or exceeds 1000 maternal deaths per 100,000 live births (54).

Statistical analyses

~~The dataset from each country was combined to create appended data.~~ Data extraction, coding, and analysis were performed using STATA software version 14.2. As the outcome variable was not equally distributed across each cluster, we weighted the sample to ensure its representativeness and alignment with the actual population of each country. Given the hierarchical nature of the DHS, multilevel analysis was conducted to identify significantly associated factors. In order to assess the variability across the 21 countries, we carried out the calculation of the Intraclass Correlation Coefficient (ICC) and Proportional Change in Variance (PCV). The Likelihood Ratio test (LR) and Median Odds Ratio (MOR) were computed to assess the variation between clusters. Variables with a p-value ≤0.25 in the bi-variable analysis were included in the multivariable analysis, and four models were constructed. These models included the null model (with no predictors), model I (adjusted for individual-level variables only), model II (adjusted for community-level variables only), and model III (adjusted for both individual and community-level variables simultaneously). The nested models were compared using deviance (−2 log-likelihood). Adjusted Odds Ratios (AOR) with 95% confidence intervals and p<0.05 were used to identify significant predictors. The variance inflation factor (VIF) test was conducted to check for multicollinearity and found no issues as all variables had VIF <5, with model III VIF at 1.74.

Ethical approval and consent to participate

We have obtained permission from the DHS program to access the data online at http://www.dhsprogram.com and to download and use the data freely. The original collection of DHS data adhered to international and national ethical guidelines, with ethical clearance approved by the ICF Macro Institutional Review Board, the Centers for Disease Control and Prevention (CDC), and the Institutional Review Board (IRB) in each country, in accordance with the United States Department of Health and Human Services requirements for human subject protection. Written consent was obtained from all respondents and/or their legal guardians for minors (aged below 16) to participate, in line with Helsinki declarations. Importantly, the procedures for DHS public-use datasets ensure that respondents, households, or sample communities cannot be identified in any way, as the data files do not contain personal identifiers of individuals or household addresses. Furthermore, this study is not an experimental study. For more details on the ethical standards and data usage by the DHS, please refer to: http://goo.gl/ny8T6X.

RESULTS

Socio-demographic, economic, and health service-related characteristics of respondents

The study included a total of 64,076 women of reproductive age, with 47.09% falling into the 15-24 age group. Approximately, half of the participants (50.29%) had attended a secondary education or higher, and about 30.47% were categorized as the richest. In this study, 63.12% of the household heads were female, and 53.04% of the respondents lived in rural areas. Furthermore, 76.11% of the respondents had access to an improved source of drinking water (Table 2).

Table 2. Socio-demographic and health service-related characteristics of respondents in low and middle-income countries with high maternal mortality

Variables	Categories	Unweighted frequency (%)	Weighted frequency (%)
Country	Benin	2586 (4.10)	2584 (4.03)
	Burundi	3117 (4.91)	3008 (4.70)
	Democratic Republic of the Congo (DRC)	2751 (4.34)	2906 (4.53)
	Cote d'Ivoire	2927 (4.61)	3027 (4.72)
	Cameroon	3048 (4.81)	3000 (4.68)
	Gambia	2361 (3.72)	2540 (4.00)
	Guinea	2044 (3.22)	2053 (3.20)
	Haiti	5213 (8.21)	5306 (8.28)
	Kenya	7599 (11.98)	8025 (12.53)
	Liberia	1495 (2.36)	1425 (2.22)
	Lesotho	1798 (2.83)	1784 (2.78)
	Madagascar	3528 (5.56)	3570 (5.57)
	Mali	1542 (2.43)	1451 (2.26)
	Mauritania	3180 (5.01)	3181 (4.96)
	Malawi	2981 (4.70)	2914 (4.55)
	Nigeria	3879 (6.12)	3783 (5.90)
	Niger	1131 (1.78)	1023 (1.60)
	Siera Leone	3285 (5.18)	3373 (5.26)
	Chad	2837 (4.47)	2981 (4.65)
	Togo	1941 (3.06)	2038 (3.18)
	Zimbabwe	4186 (6.60)	4100 (6.40)
Age of women	15-24	29884 (47.11)	30174 (47.09)
	25-34	10057 (15.86)	10359 (16.17)
	35-49	23488 (37.03)	23542 (36.74)
Women educational status	No education	15492 (24.42)	14766 (23.04)

Variables	Categories	Unweighted frequency (%)	Weighted frequency (%)
	Primary education	17064(26.90)	17085 (26.66)
	Secondary and above	30873 (48.67)	32225 (50.29)
Women occupation	Not working	24520 (40.36)	24509 (39.81)
	Working	36235 (59.64)	37058 (60.19)
Women marital status	Single	31000 (48.87)	31481 (49.13)
	Married	25492 (40.19)	25549 (39.87)
	Widowed	2598 (4.10)	2579 (4.02)
	Divorced	4339 (6.84)	4467 (6.97)
Number of household members	1-4	20732 (32.68)	21214 (33.11)
	5-10	35455 (55.90)	35640 (55.62)
	>10	7242 (11.42)	7221 (11.27)
Wealth index	Poorest	8757 (13.81)	7785 (12.15)6
	Poorer	9976 (15.73)	9756 (15.23)
	Middle	12575 (19.83)	12108 (18.90)
	Richer	14567 (22.97)	14906 (23.26)
	Richest	17554 (27.68)	19521 (30.47)
Current contraceptive method	No Method	50638 (79.83)	50821 (79.31)
	Modern Method	11360 (17.91)	11678 (18.22)
	Traditional Method	1431 (22.56)	1577 (2.46)
Smoking	No	60043 (99.1)	60512 (99.10)
	Yes	549 (0.90)	584 (0.9)
Sex of household head	Male	40184 (63.35)	40448 (63.12)
	Female	23245 (36.65)	23628 (36.88)
Number of living children	No child	34455 (54.32)	34906 (54.48)
	One child	4913 (7.75)	5163 (8.10)
	2-3 children	10502 (16.56)	10792 (16.84)
	4 and above children	13559 (21.38)	13215 (20.62)
Frequency of watching television	Not at all	31491 (49.68)	30584 (47.77)
	Less than once a week	10254 (16.18)	10236 (15.99)
	At least once a week	21641 (34.14)	23207 (36.25)
Frequency of reading newspaper or magazine	Not at all	49409 (77.93)	49253 (76.91)
	Less than once a week	8305 (13.10)	8662 (13.53)
	At least once a week	5690 (8.97)	6123 (9.56)
Frequency of listening to radio	Not at all	25681 (40.49)	24937 (38.92)
	Less than once a week	13880 (21.88)	14220 (22.19)
	At least once a week	23868 (37.63)	24918 (38.89)
Frequency of using internet	Not at all	39557 (74.68)	38666 (72.48)
	Less than once a week	1496 (2.82)	1570 (2.94)
	At least once a week	11918 (22.50)	13107 (24.57)
Source of drinking water	Unimproved	15970 (25.18)	15304 (23.88)

Variables	Categories	Unweighted frequency (%)	Weighted frequency (%)
	Improved	47,456 (74.82)	48,769 (76.11)
Residence	Urban	28811 (45.42)	30088 (46.96)
	Rural	34618 (54.58)	33988 (53.04)
Distance to health facility	Big problem	19736 (32.57)	19224 (31.47)
	Not a big problem	40854 (67.43)	41869 (68.53)
Community-level women education	Low	32068 (50.56)	31242 (48.76)
	High	31361 (49.44)	32833 (51.24)
Community poverty	Low	31633 (49.87)	33598 (52.43)
	High	31796 (50.13)	30478 (47.56)
Community-level media usage	Low	26760 (50.52)	25531 (47.86)
	High	26211 (49.48)	27812 (52.14)

251

Prevalence of overweight/obesity among reproductive-age women in low and middle-income countries with high maternal mortality

The pooled prevalence of overweight/obesity among reproductive-age women in low and middle-income countries with high maternal mortality was 32% (95% CI: 27 – 37), with the highest in Mauritania (53%) and the lowest in Burundi (10%) (Figure2).

Multilevel logistic regression analysis of overweight/obesity among reproductive age women in low and middle-income countries with high maternal mortality

The random effects analysis revealed that the Inter Cluster Correlation Coefficient (ICC) of the null model was 0.19, indicating that nineteen percent of the total variability in the prevalence of overweight/obesity was due to between cluster variability, while eighty-one percent was due to individual differences. The MOR in the null model was 2.30, suggesting that a woman of reproductive age from a cluster with high overweight/obesity prevalence has a 2.30 times higher chance of being affected than a woman from a cluster with a lower prevalence. The best-fitted model was Model III, which had the highest log likelihood (-27625) and the lowest deviance (55250) among all fitted models. Model III had a Proportional Change in Variance (PCV) of 29%, indicating that 29% of the total variability in overweight/obesity prevalence was explained by the full model (Table 3).

In a study aiming to identify the factors contributing to overweight/obesity among women of reproductive age in low and middle-income countries with high maternal mortality, a multivariable analysis was carried out. According to the best-fitted model (Model III), both individual and community-level variables were found to be significant factors in overweight/obesity among women of reproductive age.

The individual-level factors included women aged 25–34 years and 35-49 years, those with primary education, secondary education and above, non-working women, married, widowed, and divorced women, as well as women belonging to different wealth tiers and those heading female-led households. Additionally, the number of children, frequency of television and internet usage, and access to improved sources of drinking water were also identified as individual-level factors.

On the other hand, urban residence, low community poverty, and high community-level media usage were found to be significantly associated variables at the community level. These findings are outlined in Table 3.

Table 3. Multivariable multilevel logistic regression analysis results of both individual-level and community-level factors associated with overweight/obesity in low and middle-income countries with high maternal mortality

Variables	Categories	Null model	Model I AOR [95% CI]	Model II AOR [95% CI]	Model III AOR [95% CI]
Age of women	15-24		1.00		1.00
	25-34		**2.44 (2.25 - 2.65)*** **		**2.42 (2.23 - 2.63)*** **
	35-49		**3.49 (3.17 – 3.84)*** **		**3.47 (3.15 - 3.83)*** **
Women educational status	No education		1.00		1.00
	Primary education		**1.10 (1.03 - 1.18)** **		**1.10 (1.02 - 1.18)** **
	Secondary and above		**1.10 (1.02 – 1.19)** **		**1.10 (1.01 - 1.18)** **
Women occupation	Not working		**1.07 (1.02 – 1.13)* **		**1.06 (1.01 – 1.12)* **
	Working		1.00		1.00
Women marital status	Single		1.00		1.00
	Married		**1.96 (1.81 – 2.13)*** **		**1.98 (1.82 – 2.15)*** **
	Widowed		**1.39 (1.22 – 1.59)*** **		**1.39 (1.22 – 1.59)*** **
	Divorced		**1.71 (1.54 –1.90)*** **		**1.70 (1.53 –1.88)*** **
Number of household members	1-4		1.00		1.00
	5-10		1.00 (0.95 – 1.05)		1.00 (0.95 - 1.05)
	>10		1.03 (0.94 - 1.12)		1.03 (0.94 - 1.12)
Wealth index	Poorest		1.00		1.00
	Poorer		**1.33 (1.21 - 1.47)*** **		**1.30 (1.18 - 1.44)*** **
	Middle		**1.93 (1.75 – 2.13)*** **		**1.75 (1.59 - 1.94)*** **
	Richer		**2.44 (2.21 -2.70)*** **		**1.98 (1.77 - 2.21)*** **
	Richest		**3.16 (2.84 – 3.52)*** **		**2.38 (2.11 - 2.69)*** **
Current contraceptive method	No Method		0.95 (0.89 – 1.00)		0.95 (0.89 – 1.00)
	Modern Method		1.00		1.00
	Traditional Method		1.13 (0.98 – 1.31)		1.12 (0.97 – 1.30)
Smoking	No		1.00		1.00
	Yes		0.94 (0.77 – 1.15)		0.93 (0.76 – 1.14)
Sex of household head	Male		1.00		1.00
	Female		**1.18 (1.12 - 1.24)*** **		**1.15 (1.09 - 1.22)*** **
Number of living children	No child		1.00		1.00
	One child		**1.28 (1.16 - 1.41)*** **		**1.27 (1.15 - 1.40)*** **
	2-3 children		**1.45 (1.31 - 1.59)*** **		**1.44 (1.31 - 1.59)*** **
	4 and above children		**1.34 (1.20 - 1.49)*** **		**1.34 (1.21 - 1.50)*** **
Frequency of watching television	Not at all		1.00		1.00
	Less than once a week		**1.39 (1.30 - 1.50)*** **		**1.30 (1.21 - 1.39)*** **

	At least once a week		**1.55 (1.46 - 1.66)*** **		**1.41 (1.32 - 1.51)*** **
Frequency of reading newspaper or magazine	Not at all		1.00		1.00
	Less than once a week		1.03 (0.97 – 1.11)		1.04 (0.97 – 1.11)
	At least once a week		1.00 (0.92 – 1.10)		1.00 (0.92 – 1.10)
Frequency of listening to radio	Not at all		1.00		1.00
	Less than once a week		0.98 (0.92 – 1.05)		0.97 (0.91 – 1.03)
	At least once a week		0.98 (0.92 – 1.03)		0.97 (0.92 – 1.03)
Frequency of using internet	Not at all		1.00		1.00
	Less than once a week		**1.50 (1.32 - 1.71)*** **		**1.45 (1.28 - 1.65)*** **
	At least once a week		**1.74 (1.64 - 1.85)*** **		**1.67 (1.57 - 1.78)*** **
Source of drinking water	Unimproved		1.00		1.00
	Improved		**1.12 (1.05 - 1.19)*** **		**1.08 (1.01 - 1.15)* **
**Community-level variables**					
Residence	Urban			**1.48 (0.38 – 1.58)*** **	**1.38 (1.28 – 1.49)*** **
	Rural			1.00	1.00
Distance to health facility	Big problem			1.00	1.00
	Not a big problem			**1.12 (1.07 - 1.18)*** **	1.04 (0.99 - 1.10)
Community-level women's education	Low			1.00	1.00
	High			**1.09 (1.03 - 1.45)** **	1.02 (0.95 - 1.09)
Community poverty	Low			**1.52 (1.42 – 1.63)*** **	**1.10 (1.01 – 1.20)** **
	High			1.00	1.00
Community-level media usage	Low			1.00	1.00
	High			**1.46 (1.38 - 1.55)*** **	**1.20 (1.12 - 1.29)*** **
**Random effect**					
	Variance	0.77	0.56	0.48	0.55
	ICC	0.19	0.15	0.13	0.14
	MOR	2.30	2.04	1.93	2.02
	PCV	Reff	27.30	38.00	29.00
**Model comparison**					
	Log likelihood ratio	-38895	-27706	-32282	-27625
	Deviance	77790	55412	64564	55250
	Mean VIF		1.64	1.40	1.74
* = P-value < 0.05, ** = P-value < 0.01, *** = P-value < 0.001					
ICC = Inter cluster corrolation cofficent , MOR =Median odds ratio, PCV = proportional change in variance. AOR=adjusted odds ratio; CI= confidence interval, VIF =variance inflation factor					

Discussion

Overweight/obesity in women of reproductive age (15-49 years old) is a major public health problem in low and middle-income countries. Early detection and prevention of overweight/obesity can greatly improve the quality of life for women and households. To address this issue, it is crucial to determine the prevalence of overweight/ obesity and identify associated factors. This study assessed the pooled prevalence of overweight /obesity and its associated factors among women of reproductive age (15-49 years old) in low and middle-income countries with high maternal mortality using recent DHS surveys. Our research revealed that 32% of women of reproductive age are overweight/obese, indicating that more than three out of ten women fall into this category. The study found that Mauritania has the highest prevalence of overweight/obesity at 53%, while Burundi has the lowest at 10%. These disparities among countries might be attributed to variations in socio-demographic and economic factors, lifestyle differences such as food preferences, cultural beliefs regarding dietary habits, and disparities in government and policymakers' efforts to address overweight/obesity (20, 60, 61).

This study found that both individual-level and community-level factors were significantly associated with overweight/obesity in women of reproductive age. These factors include older age, higher education, better economic status, having more living children, frequent television watching, frequent internet usage, not being part of the workforce, marital status, female household head, access to improved drinking water, urban residence, lower community poverty, and higher community-level media usage. These factors were associated with a higher prevalence of overweight/obesity.

As observed in previous research conducted in various settings (14, 19-21, 27, 41, 43), our study also found that women aged 25-34 years and 35-49 years were more likely to experience weight gain. This positive association might be attributed to physiological changes that occur with age, such as hormonal shifts, increased body fat, and changes in body composition (41). Additionally, reduced participation in physical activities due to age-related physiological changes among women may contribute to the increased weight gain (62-64).

This study has established a clear association between women's education and overweight/obesity in low and middle-income countries with high maternal mortality. It was found that women of

reproductive age with primary, secondary, and higher education were more likely to be overweight/obese compared to those with no education. This result is consistent with previous studies conducted in other locations (7, 9, 18, 41). The reason behind this trend could be that educated women tend to have higher incomes, leading to changes in lifestyle such as dietary habits and consumption of calorie-dense foods and high-fat diets (4, 41).

However, it is important to note that the finding of educational status in our study differ from some other previous studies (65, 66). This variation could be attributed to differences in research methodologies and the datasets used. Our study utilized non-pregnant women's data from the Demographic and Health Surveys (DHS) of 21 countries, with nutritional status measured by BMI. In contrast, the aforementioned studies used BMI data from pregnant women, which might not be the recommended method for assessing nutritional status. In clinical practice, the recommended assessment modality for pregnant women’s nutritional status is Mid-Upper Arm Circumference (MUAC), not BMI. Additionally, our study excluded pregnant women in line with the DHS-7 guidelines to ensure reliable estimates. Another potential reason for the disparity in findings could be the differences in the populations studied. Our research focused on developing countries, while the other studies were conducted in more developed nations. It's plausible that women in developed countries might leverage their advanced education to manage weight gain better.

Not working women of reproductive age have been found to have higher odds of overweight/obesity compared to their working counterparts. This aligns with findings from studies conducted in Ethiopia and Bangladesh (15, 43). This could be attributed to lower levels of physical activity related to occupation among non-working women, leading to weight gain (67, 68). Additionally, the association between not working women and higher frequencies of television watching or internet use may also contribute to this trend. Previous studies have indicated a strong link between television watching and a sedentary lifestyle, ultimately resulting in increased weight gain (20, 69). Consequently, not working women of reproductive age might have less exposure to physical activity due to the absence of work, potentially increasing their likelihood of gaining weight.

In low and middle-income countries with high maternal mortality, this research indicates a significant correlation between individual-level factors such as frequent television watching and internet usage, as well as community-level media exposure, and the prevalence of

overweight/obesity among women of reproductive age. The findings align with similar studies conducted in Mali (20) and Timor-Leste (17). It is suggested that high levels of television viewing, internet usage, and community media exposure may contribute to abnormal metabolism associated with a sedentary lifestyle (69), potentially leading to increased weight gain (70). Furthermore, the frequent use of media could potentially replace the time that would otherwise be spent on physical activity, resulting in lower energy expenditure and subsequent weight gain (71).

The study findings indicate a direct relationship between wealth quantile and overweight/obesity. As household income increases, the likelihood of weight gain also increases. Additionally, lower community poverty is significantly associated with higher odds of weight gain in women of reproductive age. These findings are consistent with several previous studies (8, 14, 15, 18-22, 40, 41, 43-45). One possible explanation for this is that in developing countries, individuals with higher wealth are more inclined to consume energy-dense foods and lead sedentary lifestyles (40, 72). Moreover, women in higher wealth quantiles might have a higher caloric intake compared to those in lower quantiles, which could be a primary factor contributing to weight gain.

The odds of overweight/obesity among urban women of reproductive age were higher compared with women in rural areas. This finding is supported by previous studies (8, 10, 16, 21, 41). Research has shown that urbanization significantly influences dietary patterns. The presence of fast-food restaurants offering energy-dense foods (72) and supermarkets might contribute to the higher rates of overweight/obesity. Additionally, urbanization tends to decrease physical activity levels as residents often rely on transportation instead of walking. In contrast, rural residents are more exposed to physical activities due to limited transportation options such as taxis (16). Therefore, for the reasons mentioned above, urban women of reproductive age might be more susceptible to overweight/obesity compared to their rural counterparts.

In low and middle-income countries with high maternal mortality, married, widowed, or divorced women were found to have a higher prevalence of overweight /obesity compared to single women. This observation is consistent with previous studies conducted in various settings (8, 10, 16, 19-21). The possible explanation for this finding is that women who are not single might have a greater likelihood of having had multiple children in previous pregnancies. Consequently, these women might be at a higher risk of weight gain during and after pregnancy due to a more sedentary lifestyle and consumption of energy-dense foods (72, 73). Our study also indicated that the odds of

overweight/obesity among women of reproductive age who had children were higher than those who had not given birth. This finding aligns with studies conducted in Cambodia (16) and Guinea (45). The rationale behind this association could be that women with children might have less time for managing their weight due to the demands of childcare (16). Furthermore, having more children is associated with multiple pregnancies, which in turn might lead to depression and anxiety. These psychological factors could potentially contribute to abnormal obesity through hypothalamic-pituitary abnormal hyperactivity (74).

The study revealed that female heads of households were more likely to be overweight or obese compared to other household heads. This could be attributed to the stress, depression, and anxiety experienced by female household heads (75). These conditions are linked to abnormal hormonal function, which can lead to unhealthy weight gain (27, 74). Additionally, the study found that reproductive-age women with improved sources of drinking water had higher odds of being overweight or obese compared to those with unimproved sources. The study highlighted borehole water as a significant component of improved drinking water sources, and this water is known to be salty. Therefore, women with higher salt intake might have a greater likelihood of increased caloric intake, food consumption, and consumption of meat and snacks, which directly contributes to obesity (76). Another potential explanation could be the presence of bottled water as part of the improved drinking water sources in the study. The study suggested that the chemicals in plastic bottles might contribute to weight gain by altering metabolism and promoting the growth of fat cells. Interestingly, the study confirmed that 94% of reproductive-age women who used bottled water were overweight or obese (77).

Our study had some strengths and limitations. Among the strengths of this study, it was analyzed using pooled data from 21 nationally representative DHS surveys in low and middle-income countries with high maternal mortality. In addition, we weighted our sample size to get reliable estimates and standard errors. As a limitation, the DHS used a cross-sectional survey design, and the causal relationship between reproductive-age women's overweight/obesity and the independent variables cannot be established.

Conclusion and recommendation

The prevalence of overweight/obesity among women of reproductive age (15-49 years old) was found to be relatively high in low and middle-income countries with high maternal mortality. The result indicated that more than three out of ten women in this age group were overweight/obese. Various individual-level and community-level factors were associated with overweight/obesity. Women of reproductive age who were older, more educated, wealthier, had children, watched television, used the internet, had access to improved drinking water sources, and lived in urban areas were more likely to be overweight or obese. Additionally, the odds of being overweight or obese were higher for female heads of households, non-working women, married, widowed, and divorced women. It is recommended to pay special attention to women of reproductive age who have received formal education, are not in the workforce, engage in television viewing and internet usage, live in urban areas, are female heads of households, and are among the wealthiest group of women.

Abbreviations

AOR, Adjusted Odds Ratio; BMI, body mass index; CI, Confidence Interval; CDC, Centers for Disease Control and Prevention; CHF, Congestive Heart Failure; CKD, Chronic Kidney Disease; DHSs, Demographic and Health Surveys; DALYs disability-adjusted life years; ICC, Intracluster correlation coefficient; IR, individual record; IRB, Institutional Review Board; LMIC, Low and Middle-Income Countries; MOR, median odds ratio; MUAC, Mid-Upper Arm Circumference; PCV, a proportional change in variance; UNICEF, United Nations International Children's Emergency Fund; VIF, Variance Inflation Factor; WHO, World Health Organization

Declarations

Consent for publication

Not applicable.

Availability of data and materials

The data used for the analysis of this study is attached as supplementary file 1.

Competing interests

The authors declare that they have no competing interests.

Funding

No funder.

Author Contributions

Conceptualization: DMG. Study design: DMG, KMA, YT, AE, WDN, AMW, LY, MG, NW, AYB, LA, HTA, MGT, AH, MJ, and KAD. Execution: DMG, KMA, YT, AE, WDN, AMW, LY, MG, NW, AYB, LA, HTA, MGT, AH, MJ, and KAD. Acquisition of the data: DMG, KMA, YT, AE, WDN, AMW, LY, MG, NW, AYB, LA, HTA, MGT, AH, MJF, and KAD. Analysis and interpretation: DMG, KMA, YT, AE, WDN, AMW, LY, MG, NW, AYB, LA, HTA, MGT, AH, MJF, and KAD. Writing: DMG, KMA, YT, AE, WDN, AMW, LY, MG, NW, AYB, LA, HTA, MGT, AH, MJ, and KAD. Review and editing: DMG, KMA, YT, AE, WDN, AMW, LY, MG, NW, AYB, LA, HTA, MGT, AH, MJ, and KAD. All authors have read and approved the final version of the manuscript.

Acknowledgments

The authors would like to thank measure DHS for their permission to access the dataset.

References

1. Organization WH. Diet, nutrition, and the prevention of chronic diseases: report of a joint WHO/FAO expert consultation: World Health Organization; 2003.
2. Lim SS, Vos T, Flaxman AD, Danaei G, Shibuya K, Adair-Rohani H, et al. A comparative risk assessment of burden of disease and injury attributable to 67 risk factors and risk factor clusters in 21 regions, 1990–2010: a systematic analysis for the Global Burden of Disease Study 2010. The lancet. 2012;380(9859):2224-60.
3. Afshin A, Forouzanfar MH, Reitsma MB, Sur P, Estep K, Lee A, et al. Health Effects of Overweight and Obesity in 195 Countries over 25 Years. The New England journal of medicine. 2017;377(1):13-27.
4. Ford ND, Patel SA, Narayan KM. Obesity in Low- and Middle-Income Countries: Burden, Drivers, and Emerging Challenges. Annual review of public health. 2017;38:145-64.
5. Swinburn BA, Sacks G, Hall KD, McPherson K, Finegood DT, Moodie ML, Gortmaker SL. The global obesity pandemic: shaped by global drivers and local environments. The Lancet. 2011;378(9793):804-14.
6. Ng M, Fleming T, Robinson M, Thomson B, Graetz N, Margono C, et al. Global, regional, and national prevalence of overweight and obesity in children and adults during 1980–2013: a systematic analysis for the Global Burden of Disease Study 2013. The lancet. 2014;384(9945):766-81.
7. Chowdhury MAB, Adnan MM, Hassan MZ. Trends, prevalence and risk factors of overweight and obesity among women of reproductive age in Bangladesh: a pooled analysis of five national cross-sectional surveys. BMJ open. 2018;8(7):e018468.
8. Sartorius B, Veerman LJ, Manyema M, Chola L, Hofman K. Determinants of Obesity and Associated Population Attributability, South Africa: Empirical Evidence from a National Panel Survey, 2008-2012. PloS one. 2015;10(6):e0130218.
9. Neupane S, KC P, Doku DT. Overweight and obesity among women: analysis of demographic and health survey data from 32 Sub-Saharan African Countries. BMC public health. 2015;16(1):1-9.
10. Mkuu R, Barry A, Yonga G, Nafukho F, Wernz C, Gilreath T, et al. Prevalence and factors associated with overweight and obesity in Kenya. Preventive medicine reports. 2021;22:101340.
11. Djalalinia S, Yoosefi M, Shahin S, Ghasemi E, Rezaei N, Ahmadi N, et al. The levels of BMI and patterns of obesity and overweight during the COVID-19 pandemic: Experience from the Iran STEPs 2021 survey. Frontiers in Endocrinology. 2022;13:1043894.
12. Mendez MA, Monteiro CA, Popkin BM. Overweight exceeds underweight among women in most developing countries. The American journal of clinical nutrition. 2005;81(3):714-21.
13. Jones-Smith JC, Gordon-Larsen P, Siddiqi A, Popkin BM. Is the burden of overweight shifting to the poor across the globe? Time trends among women in 39 low-and middle-income countries (1991–2008). International journal of obesity. 2012;36(8):1114-20.
14. Lyrio AO, Souza ES, dos S Conceição S, Batista JE, Brito SM, Gomes Filho IS, et al. Prevalence of overweight and obesity and associated factors among women of childbearing age in Brazil. Public health nutrition. 2021;24(16):5481-90.
15. Ali NB, Dibley MJ, Islam S, Rahman MM, Raihana S, Bhuiyan SET, et al. Overweight and obesity among urban women with iron deficiency anaemia in Bangladesh. Maternal & Child Nutrition. 2021;17(2):e13102.

16. Um S, Yom A, A. Muir J, Sopheab H. Overweight and obesity among women at reproductive age in Cambodia: Data analysis of Cambodia Demographic and Health Survey 2014. PLOS global public health. 2023;3(3):e0001768.
17. Talukder A, Gupta RD, Hashan MR, Haider SS, Sajal IH, Sarker M. Association between television viewing and overweight and obesity among women of reproductive age in Timor-Leste: evidence from the demographic health survey 2016. BMJ open. 2021;11(8):e045547.
18. Asosega KA, Aidoo EN, Adebanji AO, Owusu-Dabo E. Examining the risk factors for overweight and obesity among women in Ghana: A multilevel perspective. Heliyon. 2023;9(5).
19. Mosha D, Paulo HA, Mwanyika-Sando M, Mboya IB, Madzorera I, Leyna GH, et al. Risk factors for overweight and obesity among women of reproductive age in Dar es Salaam, Tanzania. BMC nutrition. 2021;7(1):1-10.
20. Seidu A-A, Ahinkorah BO, Agbaglo E, Nyaaba AA. Overweight and obesity among women of reproductive age in Mali: what are the determinants? International Health. 2021;13(5):428-35.
21. Mukora-Mutseyekwa F, Zeeb H, Nengomasha L, Kofi Adjei N. Trends in prevalence and related risk factors of overweight and obesity among women of reproductive age in Zimbabwe, 2005–2015. International Journal of Environmental Research and Public Health. 2019;16(15):2758.
22. Kinnunen TI, Bastola K, Neupane S. Trends in the prevalence of overweight and obesity among women of reproductive age. 2021.
23. Manson JE, Willett WC, Stampfer MJ, Colditz GA, Hunter DJ, Hankinson SE, et al. Body weight and mortality among women. New England Journal of Medicine. 1995;333(11):677-85.
24. Cefalu WT, Bray GA, Home PD, Garvey WT, Klein S, Pi-Sunyer FX, et al. Advances in the science, treatment, and prevention of the disease of obesity: reflections from a diabetes care editors' expert forum. Diabetes Care. 2015;38(8):1567-82.
25. Capodaglio P, Liuzzi A. Obesity: a disabling disease or a condition favoring disability? 2013.
26. Doherty DA, Magann E, Francis J, Morrison J, Newnham J. Pre-pregnancy body mass index and pregnancy outcomes. International Journal of Gynecology & Obstetrics. 2006;95(3):242-7.
27. Kulie T, Slattengren A, Redmer J, Counts H, Eglash A, Schrager S. Obesity and women's health: an evidence-based review. The Journal of the American Board of Family Medicine. 2011;24(1):75-85.
28. Leeners B, Rath W, Kuse S, Irawan C, Imthurn B, Neumaier-Wagner P. BMI: new aspects of a classical risk factor for hypertensive disorders in pregnancy. Clinical Science. 2006;111(1):81-6.
29. Bergström A, Hsieh C, Lindblad P, Lu C, Cook N, Wolk A. Obesity and renal cell cancer–a quantitative review. British journal of cancer. 2001;85(7):984-90.
30. Yeh T-L, Hsu H-Y, Tsai M-C, Hsu L-Y, Hwang L-C, Chien K-L. Association between metabolically healthy obesity/overweight and cardiovascular disease risk: a representative cohort study in Taiwan. PloS one. 2021;16(2):e0246378.
31. Raatikainen K, Heiskanen N, Heinonen S. Transition from overweight to obesity worsens pregnancy outcome in a BMI-dependent manner. Obesity. 2006;14(1):165-71.
32. Seligman LC, Duncan BB, Branchtein L, Gaio DSM, Mengue SS, Schmidt MI. Obesity and gestational weight gain: cesarean delivery and labor complications. Revista de saude publica. 2006;40(3):457-65.

33. Jiang K, Chen B, Lou D, Zhang M, Shi Y, Dai W, et al. Systematic review and meta-analysis: association between obesity/overweight and surgical complications in IBD. International Journal of Colorectal Disease. 2022;37(7):1485-96.
34. Kristensen J, Vestergaard M, Wisborg K, Kesmodel U, Secher NJ. Pre-pregnancy weight and the risk of stillbirth and neonatal death BJOG: An International Journal of Obstetrics and Gynaecology 112 (4), 403–408. 2008.
35. Ehrenberg HM, Mercer BM, Catalano PM. The influence of obesity and diabetes on the prevalence of macrosomia. American journal of obstetrics and gynecology. 2004;191(3):964-8.
36. Nucci LB, Schmidt MI, Duncan BB, Fuchs SC, Fleck ET, Britto MMS. Nutritional status of pregnant women: prevalence and associated pregnancy outcomes. Revista de saude publica. 2001;35(6):502-7.
37. Graves BW, DeJoy SA, Heath A, Pekow P. Maternal body mass index, delivery route, and induction of labor in a midwifery caseload. Journal of midwifery & women's health. 2006;51(4):254-9.
38. Marchi J, Berg M, Dencker A, Olander E, Begley C. Risks associated with obesity in pregnancy, for the mother and baby: a systematic review of reviews. Obesity Reviews. 2015;16(8):621-38.
39. Bo S, Menato G, Signorile A, Bardelli C, Lezo A, Gallo M, et al. Obesity or diabetes: what is worse for the mother and for the baby? Diabetes & metabolism. 2003;29(2):175-8.
40. Shayo GA, Mugusi FM. Prevalence of obesity and associated risk factors among adults in Kinondoni municipal district, Dar es Salaam Tanzania. BMC public health. 2011;11(1):1-7.
41. Yeshaw Y, Kebede SA, Liyew AM, Tesema GA, Agegnehu CD, Teshale AB, Alem AZ. Determinants of overweight/obesity among reproductive age group women in Ethiopia: multilevel analysis of Ethiopian demographic and health survey. BMJ open. 2020;10(3):e034963.
42. Gigante DP, Moura ECd, Sardinha LMV. Prevalence of overweight and obesity and associated factors, Brazil, 2006. Revista de saude publica. 2009;43:83-9.
43. Enyew EB, Birhanu AY, Mewosha WZ. Spatial variation of overweight/obesity and associated factor among reproductive age group women in Ethiopia, evidence from EDHS 2016. PloS one. 2022;17(11):e0277955.
44. Kumar P, Mangla S, Kundu S. Inequalities in overweight and obesity among reproductive age group women in India: evidence from National Family Health Survey (2015–16). BMC Women's Health. 2022;22(1):205.
45. Diakité D, Sidibé S, Kolié F, Camara F, Camara DM, Kourouma M, Délamou A. Prevalence and factors associated with the coexistence of overweight/obesity and anaemia among women of reproductive age in Guinea. Public Health Nutrition. 2023:1-10.
46. Worku M, Gizaw Z, Kassahun Belew A, Wagnew A, Hunegnaw MT. Prevalence and associated factors of overweight and obesity among high school adolescents in Bahir Dar City, Northwest, Ethiopia: A cross-sectional study. Journal of Obesity. 2021;2021.
47. Li M, Gong W, Wang S, Li Z. Trends in body mass index, overweight and obesity among adults in the USA, the NHANES from 2003 to 2018: A repeat cross-sectional survey. BMJ open. 2022;12(12):e065425.
48. Omer I, Derese T, Sintayehu Y. Overweight and Its Associated Factors among Women of Reproductive Age in Dire Dawa, Eastern Ethiopia, 2021: Community-Based Cross-Sectional Study. Journal of Obesity. 2022;2022.

49. Larsen TB, Sørensen HT, Gislum M, Johnsen SP. Maternal smoking, obesity, and risk of venous thromboembolism during pregnancy and the puerperium: a population-based nested case-control study. Thrombosis research. 2007;120(4):505-9.
50. Dagne S, Gelaw YA, Abebe Z, Wassie MM. Factors associated with overweight and obesity among adults in northeast Ethiopia: a cross-sectional study. Diabetes, metabolic syndrome and obesity: targets and therapy. 2019;12:391.
51. Monteiro CA. Nutrition and health. The issue is not food, nor nutrients, so much as processing. Public Health Nutrition. 2009;12(5):729-31.
52. Rivera JA, Barquera S, Gonzalez-Cossio T, Olaiz G, Sepulveda J. Nutrition transition in Mexico and in other Latin American countries. Nutrition reviews. 2004;62(suppl_2):S149-S57.
53. Rutstein SO, Rojas G. Guide to DHS statistics. Calverton, MD: ORC Macro. 2006;38.
54. Organization WH. Trends in maternal mortality 2000 to 2020: estimates by WHO, UNICEF, UNFPA, World Bank Group and UNDESA/Population Division: World Health Organization; 2023.
55. Croft T, Marshall A, Allen CK, Arnold F, Assaf S, Balian S, Bekele Y. Guide to DHS statistics: DHS-7 (version 2). Rockville, MD: ICF. 2020.
56. Organization WH. World Health Organization Obesity and Overweight.
57. Getnet M, Sisay WS, Alem AZ. Spatial distribution and associated factors of co-occurrence of overweight/obesity and Anemia among women in the reproductive age in sub-Saharan Africa: A multilevel analysis, DHS 2016–2021. PloS one. 2024;19(4):e0299519.
58. Chilot D, Belay DG, Ferede TA, Shitu K, Asratie MH, Ambachew S, et al. Pooled prevalence and determinants of antenatal care visits in countries with high maternal mortality: A multi-country analysis. Frontiers in Public Health. 2023;11:1035759.
59. Chilot D, Mondelaers A, Alem AZ, Asres MS, Yimer MA, Toni AT, Ayele TA. Pooled prevalence and risk factors of malaria among children aged 6–59 months in 13 sub-Saharan African countries: A multilevel analysis using recent malaria indicator surveys. PloS one. 2023;18(5):e0285265.
60. Malik VS, Willett WC, Hu FB. Global obesity: trends, risk factors and policy implications. Nature reviews endocrinology. 2013;9(1):13-27.
61. Dereje D, Yirgu R, Chichiabellu T. Magnitude of overweight/obesity and associated factors among high school adolescents' in Addis Ababa, Ethiopia. J Nutr Disord Ther. 2018;8(2):2161-0509.
62. Alemu E, Atnafu A, Yitayal M, Yimam K. Prevalence of overweight and/or obesity and associated factors among high school adolescents in Arada Sub city, Addis Ababa, Ethiopia. Journal of nutrition & food sciences. 2014;4(2):1.
63. Delacosta TC. Explorando associações entre sarcopenia, obesidade e osteoporose: estudo com pacientes da atenção primária em saúde. 2019.
64. Santos VRd, Araujo MYC, Cardoso MR, Batista VC, Christofaro DGD, Gobbo LA. Association of insufficient physical activity with sarcopenia and sarcopenic obesity in individuals aged 50 years or more. Revista de Nutrição. 2017;30:175-84.
65. Athukorala C, Rumbold AR, Willson KJ, Crowther CA. The risk of adverse pregnancy outcomes in women who are overweight or obese. BMC pregnancy and childbirth. 2010;10(1):1-8.
66. Holowko N, Mishra G, Koupil I. Social inequality in excessive gestational weight gain. International journal of obesity. 2014;38(1):91-6.

67. Bhurosy T, Jeewon R. Overweight and obesity epidemic in developing countries: a problem with diet, physical activity, or socioeconomic status? The Scientific World Journal. 2014;2014.
68. Monda KL, Adair LS, Zhai F, Popkin BM. Longitudinal relationships between occupational and domestic physical activity patterns and body weight in China. European journal of clinical nutrition. 2008;62(11):1318-25.
69. Sugiyama T, Healy GN, Dunstan DW, Salmon J, Owen N. Is television viewing time a marker of a broader pattern of sedentary behavior? Annals of behavioral Medicine. 2008;35(2):245-50.
70. Abolfotouh MA, Al-Alwan IA, Al-Rowaily MA. Prevalence of metabolic abnormalities and association with obesity among Saudi college students. International journal of hypertension. 2012;2012.
71. Cleland VJ, Schmidt MD, Dwyer T, Venn AJ. Television viewing and abdominal obesity in young adults: is the association mediated by food and beverage consumption during viewing time or reduced leisure-time physical activity? The American journal of clinical nutrition. 2008;87(5):1148-55.
72. Abrha S, Shiferaw S, Ahmed KY. Overweight and obesity and its socio-demographic correlates among urban Ethiopian women: evidence from the 2011 EDHS. BMC Public Health. 2016;16:1-7.
73. Huayanay-Espinoza CA, Quispe R, Poterico JA, Carrillo-Larco RM, Bazo-Alvarez JC, Miranda JJ. Peer Reviewed: Parity and Overweight/Obesity in Peruvian Women. Preventing chronic disease. 2017;14.
74. Pasquali R, Vicennati V, Cacciari M, Pagotto U. The hypothalamic-pituitary-adrenal axis activity in obesity and the metabolic syndrome. Annals of the New York Academy of Sciences. 2006;1083(1):111-28.
75. Yoosefi Lebni J, Mohammadi Gharehghani MA, Soofizad G, Khosravi B, Ziapour A, Irandoost SF. Challenges and opportunities confronting female-headed households in Iran: a qualitative study. BMC women's health. 2020;20:1-11.
76. Navia B, Aparicio A, Perea JM, Pérez-Farinós N, Villar-Villalba C, Labrado E, Ortega RM. Sodium intake may promote weight gain; results of the FANPE study in a representative sample of the adult Spanish population. Nutrición hospitalaria. 2014;29(6):1283-9.
77. Darbre PD. Endocrine disruptors and obesity. Current obesity reports. 2017;6:18-27.

Figures Legends

Figure 1: Flow chart showing the sampling procedure of DHS data of reproductive age women in countries with high maternal mortality

Figure 2: Prevalence of overweight/obesity of reproductive age women in countries with high maternal mortality

Supplementary file 1: Data used for the analysis of this study.

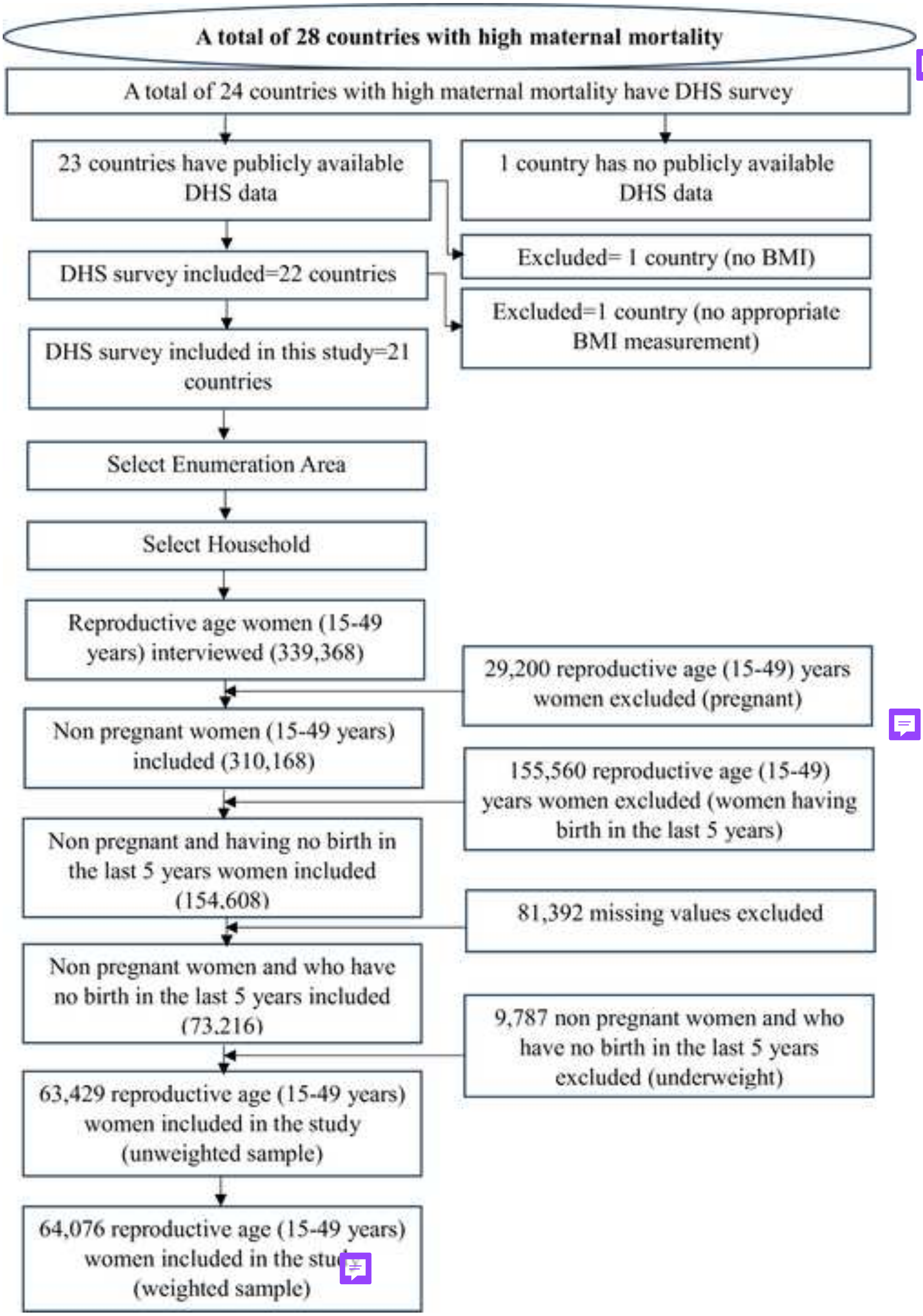
A total of 28 countries with high maternal mortality
A total of 24 countries with high maternal mortality have DHS survey
23 countries have publicly available DHS data
1 country has no publicly available DHS data
Excluded= 1 country (no BMI)
DHS survey included=22 countries
Excluded=1 country (no appropriate BMI measurement)
DHS survey included in this study=21 countries
Select Enumeration Area
Select Household
Reproductive age women (15-49 years) interviewed (339,368)
29,200 reproductive age (15-49) years women excluded (pregnant)
Non pregnant women (15-49 years) included (310,168)
155,560 reproductive age (15-49) years women excluded (women having birth in the last 5 years)
Non pregnant and having no birth in the last 5 years women included (154,608)
81,392 missing values excluded
Non pregnant women and who have no birth in the last 5 years included (73,216)
9,787 non pregnant women and who have no birth in the last 5 years excluded (underweight)
63,429 reproductive age (15-49 years) women included in the study (unweighted sample)
64,076 reproductive age (15-49 years) women included in the stu[illegible] (weighted sample)

Figure

Click here to access/download;Figure;Figure 2.tif

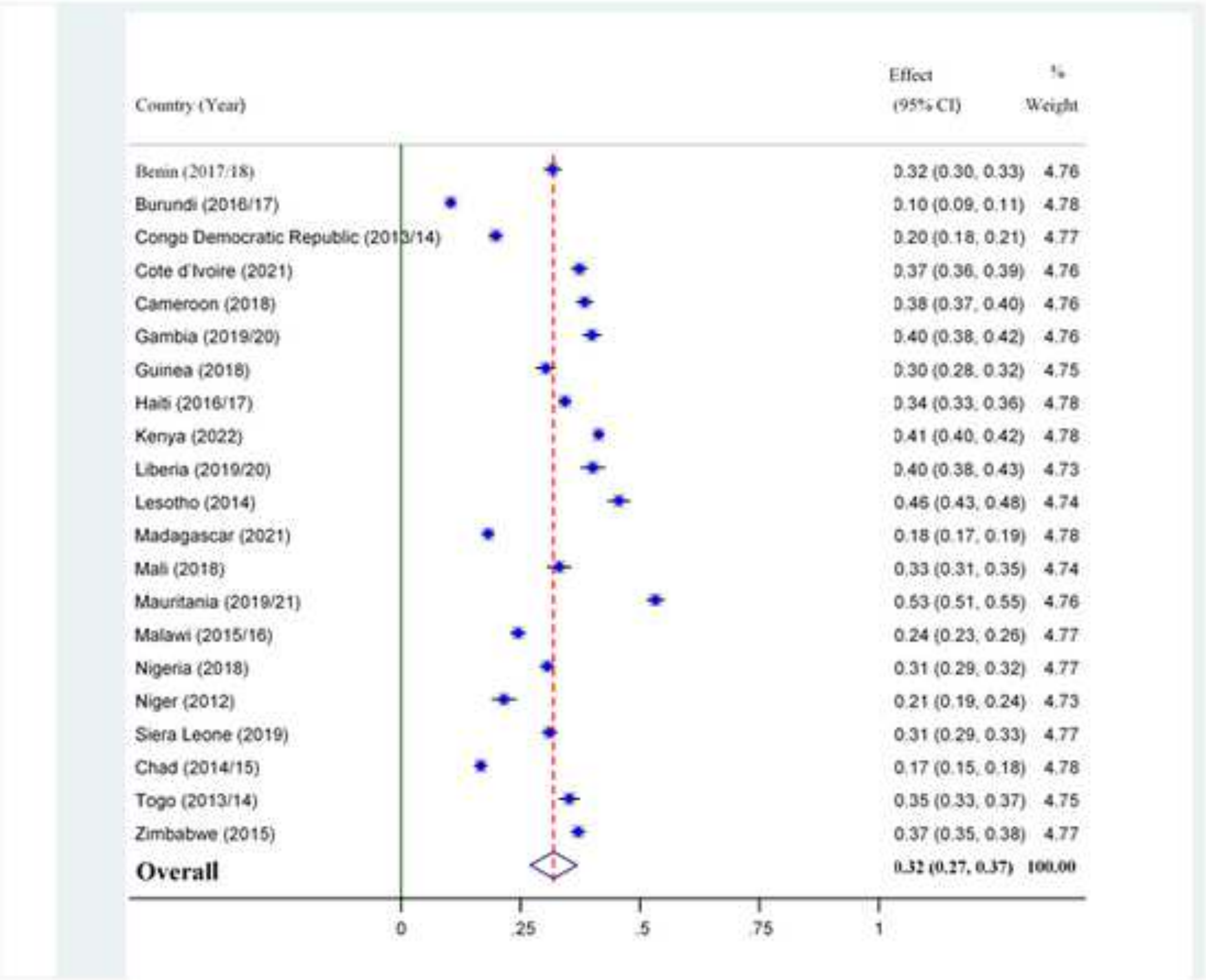

Click here to access/download
Supporting Information
Supplementary file 1.csv

Click here to access/download
Other
OTHERS.docx

Click here to access/download
Other
Other.docx

Pooled prevalence and ~~determinants~~factors of overweight/obesity among reproductive age women in low and middle-income countries with high maternal mortality: A multi-level analysis of recent Demographic and Health Surveys

Demiss Mulatu Geberu*[1], Kaleab Mesfin Abera[2], Yawkal Tsega[3], Abel Endawkie[4], Wubshet Debebe Negash[1], Amare Mesfin Workie[5], Lamrot Yohannes[6], Mihret Getnet[7,8], Nigusu Worku[1], Adina Yeshambel Belay[1], Lakew Asmare[4], Hiwot Tadesse Alemu[1], Misganaw Guadie Tiruneh[1], Asebe Hagos[1], Melak ~~Jejaw Fitigu[1]~~Jejaw[1], Kaleb Assegid Demissie[1]

[1]Department of Health Systems and Policy, Institute of Public Health, College of Medicine and Health Sciences, University of Gondar, Gondar, Ethiopia.

[2]Department of Health Systems and Policy, Institute of Public Health, College of Medicine and Health Sciences, Wollo University, Dessie, Ethiopia.

[3]Department of Health System and Management, School of Public Health, College of Medicine and Health Sciences, Wollo University, Dessie, Ethiopia.

[4]Department of Epidemiology and Biostatistics, School of Public Health, College of Medicine and Health Sciences, Wollo University, Dessie, Ethiopia.

[5]Departement of Nutrition, Institute of Public Health, College of Medicine and Health Sciences, University of Gondar, Gondar, Ethiopia.

[6]Department of Environmental and Occupational Health and Safety, Institute of Public Health, University of Gondar, Gondar, Ethiopia.

[7]Department of Human Physiology, School of Medicine, College of Medicine and Health Sciences, University of Gondar, Gondar, Ethiopia

[8]Department of Epidemiology and biostatistics, Institute of public Health, College of Medicine and Health Sciences, University of Gondar, Gondar, Ethiopia

Email addresses:

DMG: mulatu.demissma23@gmail.com

KMA: kaleabmesfin801@gmail.com

YT: yawkaltsega@gmail.com

AE: abelendawkie@gmail.com

WDN: wubshetdn@gmail.com

AMW: amare7595@gmail.com

LY: lamrotyohannes@gmail.com

MG: mihretgetnet1@gmail.com

NW: nigusuworku29@gmail.com

AYB: adinayeshambel2012@gmail.com

LA: lakewt071981@gmail.com

HTA: tadessehalemu@gmail.com

MGT: mguadie41@gmail.com

AH: asebehagos21@gmail.com

~~MJF~~MJ: melak4903@gmail.com

KAD: kalebassegid2019@gmail.com

***Correspondence:**

Demiss Mulatu Geberu

Email : mulatu.demissma23@gmail.com

P.O. Box: 196, Gondar, Ethiopia.

Formatted: Font: Times New Roman

Formatted: Font: Times New Roman, No underline, Font color: Auto

Formatted: Font: Times New Roman

Formatted: Font: Times New Roman

Formatted: Font: Times New Roman

ABSTRACT

Background: Due to the increased magnitude of overweight/obesity in many countries, the World Health Organization (WHO) ~~characterized~~has identified it as a ~~pandemia of major~~significant public health ~~concern and it was progressively higher in~~ crisis, particularly affecting women of reproductive age ~~women at Low and Middle Income Countries (LMICs).~~in developing nations. Despite obesity/overweight among women of reproductive age ~~women~~ being ~~well recognized~~widely acknowledged as a ~~common~~pressing public health ~~problem,~~issue, there has been limited investigation into its pooled prevalence and ~~different determinants have been less investigated~~various associated factors in low and middle-income countries ~~with~~(LMICs) having high maternal mortality. Thus, the objective of our study was to assess the pooled prevalence and ~~determinants~~associated factors of overweight/obesity among reproductive~~-~~-age women in low and middle-income countries with high maternal mortality.

Methods: ~~Secondary~~ We analyzed secondary data ~~analysis was conducted~~ using ~~data from~~ recent Demographic and Health Survey datasets from 21 low and middle-income countries with high maternal mortality. A ~~total of 64,076 (~~weighted sample~~)~~ of 64,076 reproductive~~-~~-age women ~~were~~was included in the analysis. ~~Variables~~The variables were extracted from the IR file ~~and~~, and the data were cleaned, recoded, and analyzed using STATA version 14.2 software ~~was used to clean, recode and analyze the data. The~~. A multilevel binary logistic regression model was ~~fitted~~applied, and adjusted odds ratios (AOR) with a 95% ~~CI~~confidence interval and a p-value ~~≤~~of ≤ 0.05 ~~was used to declare significantly~~ were utilized to identify statistically significant associated factors. ~~To check model~~Model fitness and ~~model~~ comparison~~;~~ were assessed using the ICC, MOR, PCV, and deviance (−2LLR~~) were used.~~).

Result: In this study, the pooled prevalence of overweight/obesity among reproductive~~-~~-age women was 32% (95% CI: 27% - 37~~)~~%), with a ~~large~~significant variation between countries~~ which ranged~~, ranging from 10% in Burundi to 53% in Mauritania. ~~Compared to reproductive~~ Reproductive-age women with ~~normal BMI, the following several determinants had a significant possitive association with~~ overweight/obesity~~. Women~~ showed a significant positive association with various factors compared to those with a normal BMI. These factors include being women aged 25–34 years and 35-49 years, ~~women who had primary education, who had secondary education and above, not working women,~~being married, widowed, ~~and~~or divorced~~ women, ~~, belonging to households with various income levels (poorer, middle, richer, and richest~~ household, female household head,~~), having different numbers of children (one child, 2-3 children, 4 and above children~~,~~), watching television~~ less than once a week and at least once a week~~, using the internet~~ less than once a week and at least once a week, improved source of drinking water, ~~, and being urban residents~~, low community poverty, and high community level media usage~~.

Conclusions and recommendations: More than three out of ten reproductive~~-~~-age women were overweight/obese in low and middle-income countries with high maternal mortality. Individual-level and community-level factors were associated with overweight/obesity. Special attention is recommended ~~for aged~~to older women, those ~~who had attended~~with formal education, ~~not~~ non-working women, ~~who are watching~~individuals who spend time to watch television and using the internet, urban residents, female household ~~head~~heads, and ~~richest~~ women~~.~~ in the wealthiest households.

Keywords: BMI, overweight, obesity, reproductive age women, countries with high maternal mortality

Background

~~Overweight~~The global prevalence of overweight and ~~obesity are~~obesity is a growing public health ~~problems globally. Due to the increased magnitude of the problem in many countries, the WHO~~ concern, characterized ~~it~~by the WHO as a ~~pandemia of major public health concern~~pandemic due to its impact in both developed and developing countries (1). It ~~is ranked~~ranks as the sixth~~ leading~~ highest cause of disability-adjusted life years (DALYs) and ~~accounts for~~leads to approximately 4.0 million annual deaths worldwide (2-4). ~~Besides, the~~ Additionally, there has been a 28.3% increase in global mortality ~~associated with~~ related to overweight/obesity ~~increased by 28.3%~~ from 1990 to 2015 (3). ~~Several previous studies~~Studies have ~~shown, following~~indicated that marked dietary ~~change, overweight/obesity is increasing more rapidly~~changes have resulted in a faster increase in the problem in LMICs compared to higher-income countries (4, 5). ~~A previous study conducted in 2013 confirmed~~ Out of the ~~high burden of overweight/obesity in low and middle income countries as~~ two billion ~~persons world wide were~~ overweight/obese ~~and sixty two percent of the world's obese population were dwellers of developing countries~~individuals globally, 62% were identified in LMICs (6).

~~However, several~~Multiple studies ~~depicted that~~have indicated a ~~much~~significantly higher prevalence ~~has been observed~~of the condition in –women as compared to men (3-5, 7-11). ~~Although~~Despite LMICs ~~are affecting~~being affected by ~~the double burden of~~ both overweight/obesity and underweight issues, the ~~magnitude~~prevalence of overweight ~~exceeded~~surpasses that of underweight ~~on~~among adult women (12). ~~A study carried out in 37~~A trend analysis conducted on reproductive-age women of 39 LMICs ~~revealed that~~indicated a rising prevalence of overweight–/obesity over time in the lowest economic groups compared to the highest economic groups (13).

Several studies have shown high rates of overweight and obesity among women of reproductive age in various countries. The prevalence was ~~higher progressively in reproductive age women (13).~~ reported at 55.2% in Brazil (14), 43% in Bangladesh (15), 18% in Cambodia (16), 19.4% in Timor-Leste (17), 35.5% in Ghana (18), 50.4% in Tanzania (19), and 26.9% in Mali (20). Despite interventions such as restrictions on unhealthy food advertising, improvements in school meals, taxation to reduce consumption of unhealthy foods, and supply chain incentives to promote healthy foods, the prevalence of overweight/obesity in reproductive-age women continues to rise

unacceptably (3). Trend analyses have shown substantial increases over time, with rates in Bangladesh increased from 9.35% in 1999 to 39.14% in 2014 (7), in Zimbabwe from 25% in 2005 to 36.6% in 2015 (21), and Nepal from 20.5% in 2006 to 41.1% in 2016 (22).

Previous studies found that ~~fifty three percent~~overweight/obesity was directly contributed to 53% of all female deaths~~ in women was directly attributed with overweight/obesity (14) and it is~~, serving as a well-documented risk factor for obstetric complications ~~for~~in both ~~the~~ reproductive~~-~~-age women and their future ~~newborn. The~~ offspring (23). In women of reproductive age, complications ~~in reproductive age women~~can include diabetes mellitus (8, ~~15-18~~24-27), hypertension (~~17-19~~26-28), Chronic Kidney Disease (CKD) (3, ~~20~~29), ~~Cogestive~~Congestive Heart Failure (CHF) (3, ~~21~~30), cesarean delivery and surgical site infection (~~17, 18, 22-24~~26, 27, 31-33), miscarriage and stillbirth ~~(25)~~(34), fetal macrosomia ~~(26, 27)~~(35, 36), breast and cervical cancers ~~(18)~~(27), postpartum endometritis ~~(28)~~(37), prolonged hospital stay, and postpartum hemorrhage ~~(17, 22).~~(26, 31). For newborns, complications can include low birth weight, congenital ~~malformation~~malformations, preterm birth, large-for-gestational-age babies, and perinatal death are some of the complications (~~18, 29, 30~~27, 38, 39).

Formatted: Font: 11 pt

~~Different studies revealed that the magnitude of overweight/obesity in reproductive age women was 18% in Cambodia (31), 19.4% in Timor-Leste (32), 26.9% in Mali (33), 35.5% in Ghana (34), 43% in Bangladesh (35), 50.4% in Tanzania (36), and 55.2% in Brazil (37). Nevertheless a range of interventions such as restricting the advertisement of unhealthy foods, improving school meals, using taxation to reduce consumption of unhealthy foods, and using supply chain incentives to increase production of healthy foods were implemented, to minimize and curve the burden, its prevalence is still highly increasing from time to time unacceptably in reproductive age women (3). Findings of some trend analysises ascertained this rapid progress of overweight/obesity. Accordingly, the magnitude of overweight/obesity of reproductive age women in Bangladesh increased from 9.35% in 1999 to 39.14% in 2014 (7) and increased from 25% in 2005 to 36.6% in 2015 at Zimbabwe (38). The other recent study also revealed that it was increased from 20.5% in 2006 to 41.1% in 2016 at Nepal (39).~~

Various studies ~~showed that several~~have identified multiple socioeconomic and behavioral factors ~~contributed~~contributing to overweight~~/~~ and obesity among reproductive~~-~~-age women. These factors include high socioeconomic status (1, 8, 10, 15, 18, ~~33-35, 38~~20-22, 27, 40-45), age (1, 9,

10, ~~18, 33, 35~~ 14, 15, 19-22, 27, 40, 41, 43), parity ~~(37, 39), marital status~~ (14, 22), marital status (8, 10, ~~31, 33, 36-38~~14, 16, 19-21)~~, education (10, 31, 33, 34, 39-41, 46, 47)~~, education (10, 16, 18, 20, 22, 40, 41, 46, 47), occupation status (46), urban residence (1, 8, 10, ~~31, 38~~16, 21, 41, 45), number of household members (7), contraceptive use (40, 48), number of children ~~(31, 45), and~~(16, 45), and frequency of watching television ~~(32, 33). Additionally, behavioral factors such as alcohol drinking and smoking cigarettes were potential factors~~(17, 20). Additionally, behavioral factors such as alcohol consumption, smoking, and dietary habits play a significant role (49, 50). ~~Moreover, the burden of~~

The rise in overweight/obesity ~~is~~can be attributed ~~by~~to nutritional transition from traditional whole-food-based meals ~~which consisted whole~~composed of foods like grains that have low animal source foods, salt, ~~refind~~refined oils and sugars (51) to energy dense and nutrient-poor diets composed of ~~refind~~refined carbohydrates, high ~~fate~~fat intake, and processed foods (52). ~~As a result,~~ Studies have shown that frequent consumption of sweets, meat, and eggs, ~~and eating a snack were the determinants of~~ as well as snacking, contribute to increased overweight/obesity ~~among~~in women of reproductive age ~~women~~ (1, 8, ~~38~~21)~~. Other studies also revealed that obesity/overweight is determined by~~. Moreover, lower levels of physical ~~activities~~activity and certain ethnicities have been identified as determinants of overweight/obesity (8, ~~18, 36~~19, 27, 47) ~~and ethnicity (8).~~ .

~~Despite~~It is widely acknowledged that obesity~~/~~ and overweight among women of reproductive age ~~women being well recognized as~~pose a ~~common~~significant public health ~~problem, its~~ concern. However, there has been limited investigation into the pooled prevalence and ~~different determinants have been less investigated~~ contributing factors in low and middle-income countries ~~with~~having high maternal mortality using recent ~~Demographic and Health Servey (~~DHS~~)~~ data. ~~Besides, as the global community strives to develop curation and prevention policies to address the burden of overweight/obesity, timely sufficient~~Timely and comprehensive information ~~about its~~on the rapid ~~progress~~progression and health impacts ~~at reproductive age women in countries with high maternal mortality is needed. In this regard, to prevent~~of obesity~~/~~ and overweight~~-related~~ is crucial for the development of effective prevention policies. To address the mortality and morbidity associated with overweight/obesity in women of reproductive age~~ women,~~, evidence-based interventions should be identified and implemented by healthcare providers and policy-

makers~~ should identify and implement evidence-based interventions. It is also essential to insight the reproductive age women and the community about the ~~. Educating women and communities about potential risk factors ~~of overweight/obesity and refrain from these risk factors in the future. Moreover, the finding of this study will supply~~and promoting healthy behaviors is also essential. Therefore, our study aimed to provide reliable evidence ~~for WHO's ambitious voluntary target of stopping the increment~~to support the WHO's goal of halting the rise in obesity by 2025~~. Thus, the objective of our study was to assess the pooled ~~ by assessing the prevalence and ~~determinants~~associated factors of overweight/obesity among women of reproductive age ~~women in~~in low and middle-income countries with high maternal mortality.

Materials and methods

Study design, setting, and period

The Demographic and Health Surveys (DHS) ~~used a~~are comprehensive cross-sectional ~~survey study design to collect~~surveys that gather data ~~and~~to provide population and health indicators at both the national ~~level. In~~and sub-national levels. For this study, we ~~only included~~specifically focused on low and middle-income countries ~~which have~~having high maternal mortality and available DHS data~~ such as~~, including Benin, Burundi, Chad, ~~Congo ~~Democratic Republic~~,~~ of the Congo (DRC), Cote ~~d'Ivoire~~d'Ivoire, Cameroon, Gambia, Guinea, ~~Haitie~~Haiti, Kenya, Liberia, Lesotho, Madagascar, Mali, Mauritania, Malawi, Nigeria, Niger, Sierra Leone, Togo, and Zimbabwe ~~in a study~~during the period from 2012 to 2022.

Data source and population

We ~~pooled the~~compiled data from ~~the ~~Demographic and Health Surveys (DHSs) conducted in twenty~~-~~-one ~~(Benin, Burundi, Chad, Congo Democratic Republic, Cote d'Ivoire, Cameroon, Gambia, Guinea, Haitie, Kenya, Liberia, Lesotho, Madagascar, Mali, Mauritania, Malawi, Nigeria, Niger, Sierra Leone, Togo, and Zimbabwe) ~~low and middle-income countries that have high maternal mortality (Table 1). Among these countries~~ that have high maternal mortality~~, four ~~countries don't~~do not have any available DHS reports~~ at all~~, and one ~~country hasn't publically available DHS ~~does not have publicly accessible DHS data. ~~Besides~~Additionally, one country ~~hasn't ~~does not have data on Body Mass Index (BMI~~ record~~), and another ~~one ~~country ~~hasn't~~does not have appropriate BMI ~~record. Multi~~data. Conducting a multi-country analysis using DHS

survey data is ~~simple~~feasible and ~~reasonable~~reliable, as the surveys ~~follow the same~~utilize consistent questionnaires, sampling ~~procedure~~procedures, data collection methods, and coding (53).

~~The source population was~~Our study encompassed all women of reproductive age ~~women~~ (15-49 years) ~~who lived~~residing in low and middle-income countries with high maternal mortality~~. Our study population was all reproductive age women (15-49 years) who lived in countries with high maternal mortality,~~ who had valid BMI measurements~~,~~ and were alive at the time of the survey. For this analysis, we ~~used~~utilized the individual record (IR) file ~~and we included~~, encompassing all women of reproductive age ~~women (15-49 years)~~ at the time of the survey~~. Those reproductive age women~~, excluding those who were pregnant, ~~who have births~~had given birth in the last five years, ~~and who have~~or had inappropriate BMI ~~measurement were excluded according to the~~ measurements based on DHS ~~guid. Sample weight (v005/1,000,000) was used to correct~~guidance. We adjusted for over and under-sampling ~~and~~ using the sample weight (v005/1,000,000) to ensure the generalizability of the findings. ~~As a result~~Consequently, the total weighted sample size from the ~~pooled (appended)~~combined data was 64,076~~.~~ (Figure 1).

Table1: Maternal mortality category and year of the survey by the country

Sr.No	Country	Year of DHS Survey	Maternal mortality/100,000	Category
1.	Benin	2017-18	523	~~Vey~~Very High
2.	Burundi	2016-17	494	High
3.	Chad	2014-15	1063	Extremely High
4.	~~Congo~~ Democratic Republic of the Congo (DRC)	2013-14	547	Very High
5.	Cote d'Ivoire	2021	480	High
6.	Cameroon	2018	438	High
7.	Gambia	2019-20	458	High
8.	Guinea	2018	553	Very High
9.	Haiti	2016-17	350	High
10.	Kenya	2022	530	Very High
11.	Liberia	2019-20	652	Very High
12.	Lesotho	2014	566	Very High
13.	Madagascar	2021	392	High
14.	Mali	2018	440	High
15.	Mauritania	2019-21	464	High

16.	Malawi	2015-16	381	High
17.	Nigeria	2018	1047	Extremely high
18.	Niger	2012	441	High
19.	Siera Leone	2019	443	High
20.	Togo	2013-14	399	High
21.	Zimbabwe	2015	357	High

~~*High:300-499; Very High:500-999; Extremely High:>1000*~~

~~Definition of variables~~

High:300-499; Very High:500-999; Extremely High:>1000 (54)

Data sources: http://www.dhsprogram.com, http://srhr.org/mmr2020, or http://www.who.int/publications/i/item/9789240068759

Variables and Measurements

Outcome variable:

~~BMI was the outcome variable for this study.~~

BMI was the primary variable of interest and determined by dividing weight in kilograms by the square of height in meters (55). The DHS findings uncovered that measurements were conducted on women of reproductive age (15-49 years old) in all selected households. Weight measurements were taken using portable SECA mother-infant scales with a digital display, which were developed and produced with close oversight from UNICEF. Height measurements were obtained using a Shorr measuring board. We categorized and standardized the BMI (dependent variable) into normal and overweight/obesity as per the WHO classification (56) and the guide to DHS statistics, DHS-7 (55). Accordingly, a normal BMI is within the range of 18.5 to 24.9 kg/m^2, while a BMI $\geq$25 kg/m^2 indicates overweight, and those with a BMI $\geq$30 kg/m^2 were classified as obese. For analytical purposes, reproductive age women who were overweight or obese were categorized as "yes" and those with a normal BMI were categorized as "no". We excluded underweight (mildly, moderately, and severely thin) women from both the numerator and denominator, as our focus was specifically on exploring the prevalence and associated factors of overweight/obesity as outlined in the study's topic. The decision to exclude underweight women from this analysis is in line with findings from previously published articles (41, 57).

Independent variables:

~~Independent~~In our analysis, the independent variables ~~include the ~~consist of individual-level factors such as the age~~ of women, women's,~~ educational status, ~~women's ~~occupation, ~~women's~~and marital status~~,~~ of women, as well as the number of household members, wealth index, ~~using~~use of contraceptive ~~method~~methods, smoking habits, sex of ~~hosehold~~the household head, number of living children, ~~source of driniking water, frequency of watching~~and access to media sources like television, ~~reading newspaper/magazine, listening to ~~newspapers/magazines, radio, and ~~of using~~the internet~~ were considered individual~~.

Additionally, community-level factors~~. Whereas,~~ including place of residence, distance to health ~~facility, ~~facilities, community poverty level, community~~ level~~ women's education, and community~~ level~~ media usage were ~~treated as the community level factors.~~also considered. The last three community-level variables were not directly ~~found~~available in the DHS~~. We generated those~~ dataset, so we derived them by aggregating the selected individual and household level factors at the cluster level and categorized them as higher or lower based on a median value.

~~Opperational definitions~~

~~We dichotomized the BMI as normal and overweight/obesity according to WHO classification (54). Normal BMI is from 18.5 to 24.9 kg/m^2, overweight if BMI ≥25 kg/m^2 and those with BMI ≥30 kg/m^2 were obese.~~

Community~~-~~-level ~~women's~~women's education~~: It is~~ refers to the ~~proportion~~percentage of reproductive~~-~~-age women in the community ~~that~~who have attained primary ~~and above ~~education~~.~~ ~~It was dichotomized~~ or higher. This measure is categorized as low if ~~the~~less than 50% of women ~~had <50%~~have primary ~~and above ~~education or higher, and high if ~~the~~more than 50% of women ~~had ≥ 50% primary and above~~have attained this level of education (~~55, 56~~58, 59).

Community ~~-~~poverty~~: It~~ was ~~dichotomized~~categorized as low if less than 50% of women in the ~~women's proportion of~~community fell into low wealth index categories such as poorest and poorer ~~was less than 50% ~~, and high if ~~it was greater than or equal to ~~50% ~~(55, 56)~~or more fell into these categories (58, 59).

Community~~-~~-level media usage~~: It is~~ refers to the proportion of reproductive age women in the community who watched television, ~~listend~~listened radio, read

~~newspaper/magazine~~newspapers/magazines, and used internet. ~~The categorization~~It was considered low if ~~the repondents had~~ less than 50% ~~-~~of respondents had media exposure, and high if ~~they had greater than or equal to~~ 50% ~~(55, 56)~~or more had media exposure (58, 59).

The maternal mortality ratio (MMR) is categorized as high if it falls within the range of 300-499, very high if it is between 500-999, and extremely high if it equals or exceeds 1000 maternal deaths per 100,000 live births (54).

Formatted: Space After: 10 pt

Statistical analyses

~~We append the~~ The dataset ~~of~~from each country was combined to ~~generate pooled~~create appended data. ~~The data~~Data extraction, coding, and analysis were ~~carried out~~performed using STATA software version 14.2. ~~We~~ As the outcome variable was not equally distributed across each cluster, we weighted the sample to ~~restore the~~ensure its representativeness ~~of the sample so that the total sample looks like the country's~~ and alignment with the actual population~~. Due to~~ of each country. Given the hierarchical nature of the DHS, ~~the~~ multilevel analysis was ~~fitted~~conducted to identify significantly associated factors. ~~To~~In order to assess the variability across the 21 countries, we carried out the calculation of the Intraclass Correlation Coefficient (ICC~~),~~) and Proportional Change in Variance (PCV~~), were carried out. Additionally, the~~). The Likelihood Ratio test (LR) and Median Odds Ratio (MOR) were computed to ~~measure~~assess the variation between clusters. Variables with a p-value ≤0.25 in the bi-variable analysis were included in the multivariable analysis, and four models were ~~fitted. The~~ constructed. These models included the null model (with no predictors), model ~~II~~I (adjusted for individual-level variables only), model ~~III~~II (adjusted for community-level variables only), and model ~~IV (model adjustment~~III (adjusted for both individual and community-level variables simultaneously~~) were fitted. Since the models were~~). The nested ~~we~~models were compared ~~them~~ using deviance (−2 log-likelihood). Adjusted ~~OR~~Odds Ratios (AOR) with 95% ~~CI~~confidence intervals and p<0.05 were ~~applied~~used to ~~determine~~identify significant predictors. The variance inflation factor (VIF) test was ~~done~~conducted to check for multicollinearity and ~~was not~~ found ~~because~~no issues as all variables ~~have~~had VIF <5~~. The~~, with model III VIF ~~was~~at 1.74.

Ethical approval and consent to participate

We ~~requested~~have obtained permission from the DHS ~~programme~~program to access the data ~~through~~ online at http://www.dhsprogram.com ~~-~~and ~~permission was obtained~~ to download and use the data freely. ~~International~~The original collection of DHS data adhered to international and national ethical guidelines~~ were taken into consideration during the original collection of DHS data. Ethical~~, with ethical clearance~~ for the original DHS was~~ approved by the ICF Macro Institutional Review Board, the Centers for Disease Control and Prevention (CDC~~)~~), and the Institutional Review Board (IRB) in each country, in accordance with the United ~~states~~States Department of Health and Human Services requirements for human subject protection. ~~In accordance with the DHS,~~Written consent was obtained from all respondents and/or their legal ~~guardian(s) of minors' (age~~guardians for minors (aged below 16) ~~provided written consent~~ to participate~~. All the methods were conducted according to~~, in line with Helsinki declarations. ~~It is assured that~~ Importantly, the procedures for DHS public-use datasets ~~do not in any way allow~~ ensure that respondents, households, or sample communities ~~to~~cannot be identified~~. There are no~~ in any way, as the data files do not contain personal identifiers of individuals or household addresses~~ in the data files. The~~. Furthermore, this study is not an experimental study. ~~Further explanation of how the DHS uses data and its~~For more details on the ethical standards ~~can be found at:~~ and data usage by the DHS, please refer to: http://goo.gl/ny8T6X.

RESULTS

Socio-demographic, economic, and health service-related characteristics of respondents

~~A~~The study included a total of 64,076 women of reproductive age~~ women were included in the study, of these,~~ with 47.09% ~~were in~~falling into the 15-24 age group~~ of 15-24. Almost ~~. Approximately, half of the participants (50.29%) had attended a secondary ~~and above ~~education or higher, and about 30.47% ~~of~~were categorized as the ~~reproductive age women were ~~richest. ~~Neraly two third (63.12) of sex of the household head in~~In this study~~ was ~~, 63.12% of the household heads were female~~. More than half (~~, and 53.04~~%)~~% of the respondents ~~resided~~lived in rural areas. ~~More than three fourth (~~Furthermore, 76.11~~)~~% of the ~~respndents~~respondents had access to an improved source of drinking water (Table 2).

Table 2. Socio-demographic and health service-related characteristics of respondents in low and middle-income countries with high maternal mortality

Variables	Categories	Unweighted frequency (%)	Weighted frequency (%)
Country	Benin	2586 (4.10)	2584 (4.03)
	Burundi	3117 (4.91)	3008 (4.70)
	~~Congo ~~Democratic Republic of the Congo (DRC)	2751 (4.34)	2906 (4.53)
	Cote d'Ivoire	2927 (4.61)	3027 (4.72)
	Cameroon	3048 (4.81)	3000 (4.68)
	Gambia	2361 (3.72)	2540 (4.00)
	Guinea	2044 (3.22)	2053 (3.20)
	Haiti	5213 (8.21)	5306 (8.28)
	Kenya	7599 (11.98)	8025 (12.53)
	Liberia	1495 (2.36)	1425 (2.22)
	Lesotho	1798 (2.83)	1784 (2.78)
	Madagascar	3528 (5.56)	3570 (5.57)
	Mali	1542 (2.43)	1451 (2.26)
	Mauritania	3180 (5.01)	3181 (4.96)
	Malawi	2981 (4.70)	2914 (4.55)
	Nigeria	3879 (6.12)	3783 (5.90)
	Niger	1131 (1.78)	1023 (1.60)
	Siera Leone	3285 (5.18)	3373 (5.26)
	Chad	2837 (4.47)	2981 (4.65)
	Togo	1941 (3.06)	2038 (3.18)

Formatted Table

Variables	Categories	Unweighted frequency (%)	Weighted frequency (%)
	Zimbabwe	4186 (6.60)	4100 (6.40)
Age of women	15-24	29884 (47.11)	30174 (47.09)
	25-34	10057 (15.86)	10359 (16.17)
	35-49	23488 (37.03)	23542 (36.74)
Women educational ~~-~~status	No education	15492 (24.42)	14766 (23.04)
	Primary education	17064(26.90)	17085 (26.66)
	Secondary and above	30873 (48.67)	32225 (50.29)
Women ~~-~~occupation	Not working	24520 (40.36)	24509 (39.81)
	Working	36235 (59.64)	37058 (60.19)
Women marital status	Single	31000 (48.87)	31481 (49.13)
	Married	25492 (40.19)	25549 (39.87)
	Widowed	2598 (4.10)	2579 (4.02)
	Divorced	4339 (6.84)	4467 (6.97)
Number of household members	1-4	20732 (32.68)	21214 (33.11)
	5-10	35455 (55.90)	35640 (55.62)
	>10	7242 (11.42)	7221 (11.27)
Wealth index	Poorest	8757 (13.81)	7785 (12.15)6
	Poorer	9976 (15.73)	9756 (15.23)
	Middle	12575 (19.83)	12108 (18.90)
	Richer	14567 (22.97)	14906 (23.26)
	~~Rechest~~Richest	17554 (27.68)	19521 (30.47)
Current contraceptive method	No Method	50638 (79.83)	50821 (79.31)
	Modern Method	11360 (17.91)	11678 (18.22)
	Traditional Method	1431 (22.56)	1577 (2.46)
Smoking	No	60043 (99.1)	60512 (99.10)
	Yes	549 (0.90)	584 (0.9)
Sex of household head	Male	40184 (63.35)	40448 (63.12)
	Female	23245 (36.65)	23628 (36.88)
Number of living children	No child	34455 (54.32)	34906 (54.48)
	One child	4913 (7.75)	5163 (8.10)
	2-3 children	10502 (16.56)	10792 (16.84)
	4 and above children	13559 (21.38)	13215 (20.62)
Frequency of watching television	Not at all	31491 (49.68)	30584 (47.77)
	Less than once ~~aweek~~a week	10254 (16.18)	10236 (15.99)
	At least once a week	21641 (34.14)	23207 (36.25)
Frequency of reading newspaper or magazine	Not at all	49409 (77.93)	49253 (76.91)
	Less than once ~~aweek~~a week	8305 (13.10)	8662 (13.53)
	At least once a week	5690 (8.97)	6123 (9.56)
Frequency of listening to radio	Not at all	25681 (40.49)	24937 (38.92)
	Less than once ~~aweek~~a week	13880 (21.88)	14220 (22.19)

Variables	Categories	Unweighted frequency (%)	Weighted frequency (%)
	At least once a week	23868 (37.63)	24918 (38.89)
Frequency of using internet	Not at all	39557 (74.68)	38666 (72.48)
	Less than once ~~aweek~~a week	1496 (2.82)	1570 (2.94)
	At least once a week	11918 (22.50)	13107 (24.57)
Source of ~~driniking~~drinking water	Unimproved	15970 (25.18)	15304 (23.88)
	Improved	47,456 (74.82)	48,769 (76.11)
Residence	Urban	28811 (45.42)	30088 (46.96)
	Rural	34618 (54.58)	33988 (53.04)
Distance to health facility	Big problem	19736 (32.57)	19224 (31.47)
	Not a big problem	40854 (67.43)	41869 (68.53)
Community-level women education	Low	32068 (50.56)	31242 (48.76)
	High	31361 (49.44)	32833 (51.24)
Community poverty	Low	31633 (49.87)	33598 (52.43)
	High	31796 (50.13)	30478 (47.56)
Community-level media usage	Low	26760 (50.52)	25531 (47.86)
	High	26211 (49.48)	27812 (52.14)

Prevalence of overweight/obesity among reproductive~~-~~-age women in low and middle-income countries with high maternal mortality

The pooled prevalence of overweight/obesity among reproductive~~-~~-age women in low and middle-income countries with high maternal mortality was 32% (95% CI: 27 – 37), with the highest in Mauritania (53%) and the lowest in Burundi (10%) (~~Figure1~~Figure2).

Multilevel logistic regression analysis of overweight/obesity among reproductive age women in low and middle-income countries with high maternal mortality

~~**Model comparison and**~~ The random ~~**effect**~~effects analysis

~~The result of random effect analysis in this study indicated~~ revealed that the Inter Cluster Correlation ~~Cofficent~~Coefficient (ICC) of the null model was 0.19~~ which shows~~, indicating that ~~ninetheen~~nineteen percent of the total variability in the prevalence of overweight/obesity ~~magnitude~~ was ~~related~~due to between cluster variability, while eighty~~-~~-one percent was due to individual differences. The MOR in the null model was 2.30~~ which depicted~~, suggesting that a woman of reproductive age ~~women~~ from a cluster with high overweight/obesity prevalence has a 2.30 times ~~-~~higher chance of ~~-~~being affected than a ~~reproductive age women~~ woman from a cluster with a lower ~~overweight/obesity~~ prevalence. The ~~last model (Model III) was the~~ best~~-~~-fitted model ~~because it has~~was Model III, which had the highest log likelihood ~~-~~(-27625), and the lowest deviance (55250) among all fitted models. Model III ~~has~~ had a Proportional Change in Variance (PCV) of 29~~% which indicated~~%, indicating that 29% of the total variability in overweight/obesity prevalence was explained by the full model (Table 3).

~~**Fixed effect analysis**~~

~~To~~In a study aiming to identify the ~~determinants of~~factors contributing to overweight/obesity among women of reproductive age ~~women~~in low and middle-income countries with high maternal mortality, a multivariable analysis was ~~performed. In this regard, based on~~carried out. According to the best~~-~~-fitted model (Model III), both ~~the~~ individual and community~~-~~-level variables were found to be significant ~~determinants of~~ factors in overweight/obesity among women of reproductive age~~ women in countries with high maternal mortality. Accordingly,~~.

The individual-level factors included, women aged 25–34~~ ~~ years~~, women aged~~ and 35-49 years, ~~women having~~those with primary education, secondary education and above, ~~not~~ non-working

Formatted: Font: Times New Roman, Not Bold

Formatted: Font: Times New Roman, Not Bold

Formatted: Font: Times New Roman

Formatted: Font: Times New Roman, 12 pt

women, married, widowed, and divorced women, ~~poorer, middle, richer, and richest women,~~as well as women belonging to different wealth tiers and those heading female~~ sex of household head, women having one, 2-3, four and above ~~-led households. Additionally, the number of children, ~~women watching~~frequency of television ~~less than once a week and at least once a week, women using ~~and internet ~~less than once a week and at least once a week, women having~~usage, and access to improved ~~source~~sources of drinking water were ~~from individuale ~~also identified as individual-level ~~determinants. Whereas~~factors.

On the other hand, urban residence, low community poverty, and high community-level media usage were found to be significantly associated variables ~~with overweight/obesity among~~at the community- level~~ part (~~. These findings are outlined in Table 3~~).~~.

Table 3. Multivariable multilevel logistic regression analysis results of both individual-level and community-level factors associated with ~~obesity/~~overweight/obesity in ~~East Africa~~low and middle-income countries with high maternal mortality

Variables	Categories	Null model	Model I AOR ~~-~~[95% CI]	Model II AOR [95% CI]	Model III AOR ~~-~~[95% CI]
Age of women	15-24		1.00	———	1.00
	25-34		**2.44 (2.25 - 2.65)*** **	———	**2.42 ~~-~~(2.23 - 2.63)*** **
	35-49		**3.49 (3.17 – 3.84)*** **	———	**3.47 (3.15 - 3.83)*** **
Women educational status	No education		1.00	———	1.00
	Primary education		**1.10 (1.03 - 1.18)** **	———	**1.10 (1.02 - 1.18)** **
	Secondary and above		**1.10 (1.02 – 1.19)** **	———	**1.10 (1.01 - 1.18)** **
Women occupation	Not working		**1.07 (1.02 – 1.13)* **	———	**1.06 (1.01 – 1.12)* **
	Working		1.00	———	1.00
Women marital status	Single		1.00	———	1.00
	Married		**1.96 (1.81 – 2.13)*** **	———	**1.98 (1.82 – 2.15)*** **
	Widowed		**1.39 (1.22 – 1.59)*** **		**1.39 (1.22 – 1.59)*** **
	Divorced		**1.71 (1.54 –1.90)*** **		**1.70 (1.53 –1.88)*** **
Number of household members	1-4		1.00	———	1.00
	5-10		1.00 (0.95 – 1.05)	———	1.00 (0.95 - 1.05)
	>10		1.03 (0.94 - 1.12)	———	1.03 (0.94 - 1.12)
Wealth index	Poorest		1.00	———	1.00
	Poorer		**1.33 (1.21 - 1.47)*** **	———	**1.30 (1.18 - 1.44)*** **
	Middle		**1.93 (1.75 – 2.13)*** **	———	**1.75 (1.59 - 1.94)*** **
	Richer		**2.44 (2.21 -2.70)*** **	———	**1.98 (1.77 - 2.21)*** **
	~~Rechest~~Richest		**3.16 (2.84 – 3.52)*** **	———	**2.38 (2.11 - 2.69)*** **
Current contraceptive method	No Method		0.95 (0.89 – 1.00)	———	0.95 (0.89 – 1.00)
	Modern Method		1.00	———	1.00
	Traditional Method		1.13 (0.98 – 1.31)	———	1.12 (0.97 – 1.30)
Smoking	No		1.00	———	1.00
	Yes		0.94 (0.77 – 1.15)	———	0.93 (0.76 – 1.14)
Sex of household head	Male		1.00	———	1.00
	Female		**1.18 (1.12 - 1.24)*** **	———	**1.15 (1.09 - 1.22)*** **
Number of living children	No child		1.00	———	1.00
	One child		**1.28 (1.16 - 1.41)*** **	———	**1.27 (1.15 - 1.40)*** **
	2-3 children		**1.45 (1.31 - 1.59)*** **	———	**1.44 (1.31 - 1.59)*** **
	4 and above children		**1.34 (1.20 - 1.49)*** **	———	**1.34 (1.21 - 1.50)*** **
Frequency of watching television	Not at all		1.00	———	1.00
	Less than once a week		**1.39 (1.30 - 1.50)*** **	———	**1.30 (1.21 - 1.39)*** **

	At least once a week		**1.55 (1.46 - 1.66)*** **	———	**1.41 (1.32 - 1.51)*** **
Frequency of reading newspaper or magazine	Not at all		1.00	———	1.00
	Less than once ~~aweek~~a week		1.03 (0.97 – 1.11)	———	1.04 (0.97 – 1.11)
	At least once a week		1.00 (0.92 – 1.10)	———	1.00 (0.92 – 1.10)
Frequency of listening to radio	Not at all		1.00	———	1.00
	Less than once ~~aweek~~a week		0.98 (0.92 – 1.05)	———	0.97 (0.91 – 1.03)
	At least once a week		0.98 (0.92 – 1.03)	———	0.97 (0.92 – 1.03)
Frequency of using internet	Not at all		1.00	———	1.00
	Less than once a week		**1.50 (1.32 - 1.71)*** **	———	**1.45 (1.28 - 1.65)*** **
	At least once a week		**1.74 (1.64 - 1.85)*** **	———	**1.67 (1.57 - 1.78)*** **
Source of ~~drinikingdrinking~~drinking water	Unimproved		1.00	———	1.00
	Improved		**1.12 (1.05 - 1.19)*** **	———	**1.08 (1.01 - 1.15)* **
**Community-level variables**					
Residence	Urban		———	**1.48 (0.38 – 1.58)*** **	**1.38 (1.28 – 1.49)*** **
	Rural		———	1.00	1.00
Distance to health facility	Big problem		———	1.00	1.00
	Not a big problem		———	**1.12 (1.07 - 1.18)*** **	1.04 (0.99 - 1.10)
Community-level women's education	Low		———	1.00	1.00
	High		———	**1.09 (1.03 - 1.45)** **	1.02 (0.95 - 1.09)
Community poverty	Low		———	**1.52 (1.42 – 1.63)*** **	**1.10 (1.01 – 1.20)** **
	High		———	1.00	1.00
Community-level media usage	Low		———	1.00	1.00
	High		———	**1.46 (1.38 - 1.55)*** **	**1.20 (1.12 - 1.29)*** **
**Random effect**					
	Variance	0.77	0.56	0.48	0.55
	ICC	0.19	0.15	0.13	0.14
	MOR	2.30	2.04	1.93	2.02
	PCV	Reff	27.30	38.00	29.00
**Model comparison**					
	Log likelihood ratio	-38895	-27706	-32282	-27625
	Deviance	77790	55412	64564	55250
	Mean VIF		1.64	1.40	1.74
* = P-value < 0.05, ** = P-value < 0.01, *** = P-value < 0.001					
ICC = Inter cluster corrolation cofficent , MOR =Median odds ratio, PCV = proportional change in variance. AOR=adjusted odds ratio; CI= confidence interval, VIF =variance inflation factor					

Discussion

Overweight/~~besity~~obesity in women of reproductive age ~~women~~ (15-49 years old) is a major public health problem in low and middle~~-~~-income countries. ~~Prevention and early~~Early detection ~~of the case~~and prevention of overweight/ obesity ~~has a significant advantage~~can greatly improve the quality of life for women and ~~household quality of life. Accordingly, one of the best remarkable method of bringing the attention of each individual, healthcare provider, and policy maker~~households. To address this issue, it is ~~determining~~ crucial to determine the prevalence of overweight/~~obesity prevalence and its determinants which would pointout the solution at the root of each cause. Therefore, this~~ obesity and identify associated factors. This study assessed the pooled prevalence of overweight /obesity and its ~~determinants~~associated factors among women of reproductive age ~~women in~~(15-49 years old) in low and middle-income countries with high maternal mortality using ~~a~~ recent DHS surveys. Our ~~study found~~research revealed that 32% ~~[95% CI: (27 — 37)]~~ of women of reproductive age ~~women were~~are overweight/obese, ~~which indicates~~indicating that more than three out of ten women ~~were overweight/ obese.~~fall into this category. The ~~result of this~~ study ~~depicted~~found that Mauritania has the highest ~~(53%)~~ prevalence ~~whereas~~of overweight/obesity at 53%, while Burundi has the lowest ~~(10%) prevalence of overweight/obesity in reproductive age women. This variation in~~ at 10%. These disparities among countries ~~could~~might be ~~explained by differences~~attributed to variations in socio~~-~~-demographic and economic ~~status~~factors, lifestyle ~~variations like~~differences such as food preferences, -cultural beliefs ~~about~~regarding dietary ~~consumption~~habits, and disparities in government and ~~policy makers commitment of regulating~~policymakers' efforts to address overweight/obesity (~~33, 57, 58~~20, 60, 61).

~~In this~~This study~~,~~ found that both individual-level and community-level ~~variables~~factors were ~~found to be~~ significantly associated with overweight/obesity ~~among~~in women of reproductive age ~~women. The determinants~~. These factors include ~~women aged 25 34 , 35 49 years, women attended primary~~ older age, higher education, ~~secondary education and above, not working reproductive women, married, widowed, and divorced women, poorer, middle, richer, and richest women, female sex of household head, women who had one, 2 3, four and above~~better economic status, having more living children, ~~women watched~~frequent television ~~less than once a week and at least once a week, women used~~watching, frequent internet ~~less than once a week and at least~~

~~once a week, women who had~~usage, not being part of the workforce, marital status, female household head, access to improved ~~source of~~ drinking water, ~~being~~ urban residence, ~~low~~lower community poverty, and ~~high~~higher community-level media usage~~.~~. These factors were associated with a higher prevalence of overweight/~~-~~obesity.

~~Consistent with~~ As observed in previous ~~studies~~research conducted ~~elsewhere(18, 33, 36-38, 41, 43),~~ in various settings (14, 19-21, 27, 41, 43), our study~~,~~ also found that women aged 25-34 years and 35-49 years were more likely to ~~become gain~~experience weight~~. The possible explanation for this~~ gain. This positive ~~relationship~~association might be attributed to physiological changes ~~following increment of~~ that occur with age ~~including~~, such as hormonal ~~changes, and an increase and redistribution of~~shifts, increased body fat, and changes in body composition (41). ~~Besides, the decrement of functional performance associated with physiologic change in aging women can minimize physical activity which in return leads to higher weight gain (59-61) could be another justification.~~Additionally, reduced participation in physical activities due to age-related physiological changes among women may contribute to the increased weight gain (62-64).

This study ~~confirmed the~~has established a clear association between ~~women~~women's education and overweight/obesity in low and middle-income countries with high maternal mortality. ~~A higher odds of overweight/obesity~~ It was ~~observed among~~ found that women of reproductive age ~~women~~ with primary, secondary, and higher education were more likely to be overweight/obese compared to those with no education. This ~~finding~~result is ~~also in agreement~~consistent with ~~other~~ previous studies ~~elsewhere~~conducted in other locations (7, 9, ~~34~~18, 41). ~~This~~The reason behind this trend could be ~~because~~that educated ~~reproductive age~~ women ~~relatively~~tend to have ~~a high income, and this might lead to changing lifestyles~~higher incomes, leading to changes in lifestyle such as ~~nutritional trends,~~ dietary habits and consumption of ~~high~~ calorie~~,~~-dense foods and high-fat diets (4, 41)~~. However, our finding is not similar to other previous studies (62, 63). This discrepancy could be because of methodological differences and the data they used. In our study, we used 21 countries' DHS data and revealed the pooled prevalence of those nations.~~

Formatted: Font: Times New Roman, 12 pt

~~The odds of overweight/obesity among not working reproductive age women was high compared to those working women. This finding is consistent with other studies carriedout in Ethiopia and Bangladesh ((35, 43). This could be attributed with low occupation related physical activity in women who are not working which in return increases weight gain (64, 65). Besides, the other~~

~~possible justification might be the association of not working women and high frequency of watching television or using interenet. In this regard, previous studies suggested that there is high interation between television watching and sedentary life, consequently, leads to high weight gain (33, 66). Therefore, not working reproductive age women might have less exposure while they stay home due to the absence of work compared to their counterparts and could increase their chance to gain weight.~~

~~In countries with high maternal mortality, frequency of watching television and using internet from individual level factors and community-level media usage from community level factor have a significant relationship with overweight/obesity among reproductive age women. Women who have higher frequency of watching television, using internet,and community-level media usage were more likely to become obese or gain weight. This finding is in agreement with studies conducted in Mali (33) and Timore-Leste (32). Frequent watching of television and using interenet as well as high community media usage could be related with abnormal metabolism associated with this sedentary life (66), then consequently, brings higher weight gain (67) might be the potential justification. Additionally, frequent use of media such as watching television, using internet and community media might replace the time which would be allotted for physical activity. Consequently, this could resulted in low energy expenditure and leads to higher weight gain (68), might be another possible justification.~~

~~This study revealed that wealth quantile has~~.

However, it is important to note that the finding of educational status in our study differ from some other previous studies (65, 66). This variation could be attributed to differences in research methodologies and the datasets used. Our study utilized non-pregnant women's data from the Demographic and Health Surveys (DHS) of 21 countries, with nutritional status measured by BMI. In contrast, the aforementioned studies used BMI data from pregnant women, which might not be the recommended method for assessing nutritional status. In clinical practice, the recommended assessment modality for pregnant women’s nutritional status is Mid-Upper Arm Circumference (MUAC), not BMI. Additionally, our study excluded pregnant women in line with the DHS-7 guidelines to ensure reliable estimates. Another potential reason for the disparity in findings could be the differences in the populations studied. Our research focused on developing countries, while

the other studies were conducted in more developed nations. It's plausible that women in developed countries might leverage their advanced education to manage weight gain better.

Not working women of reproductive age have been found to have higher odds of overweight/obesity compared to their working counterparts. This aligns with findings from studies conducted in Ethiopia and Bangladesh (15, 43). This could be attributed to lower levels of physical activity related to occupation among non-working women, leading to weight gain (67, 68). Additionally, the association between not working women and higher frequencies of television watching or internet use may also contribute to this trend. Previous studies have indicated a strong link between television watching and a sedentary lifestyle, ultimately resulting in increased weight gain (20, 69). Consequently, not working women of reproductive age might have less exposure to physical activity due to the absence of work, potentially increasing their likelihood of gaining weight.

In low and middle-income countries with high maternal mortality, this research indicates a significant correlation between individual-level factors such as frequent television watching and internet usage, as well as community-level media exposure, and the prevalence of overweight/obesity among women of reproductive age. The findings align with similar studies conducted in Mali (20) and Timor-Leste (17). It is suggested that high levels of television viewing, internet usage, and community media exposure may contribute to abnormal metabolism associated with a sedentary lifestyle (69), potentially leading to increased weight gain (70). Furthermore, the frequent use of media could potentially replace the time that would otherwise be spent on physical activity, resulting in lower energy expenditure and subsequent weight gain (71).

The study findings indicate a direct relationship ~~with ovwerweight~~between wealth quantile and overweight/obesity. As ~~the hosehold~~household income ~~increase~~increases, the ~~odds~~likelihood of weight gain ~~increase. Besides, from the community level factors~~also increases. Additionally, lower community poverty ~~was~~is significantly associated with higher odds of weight gain in women of reproductive age ~~women. Our finding is~~. These findings are consistent with several previous ~~several~~ studies (8, ~~33~~ 14, 15, 18-22, 40, 41, 43-45)~~. The possible justification for this could be that in the context of developing countries, the richest are more likely to consume energy-dense foods and their lifestyle is sedentary (40, 69). In addition, richest women might have increased caloric intake than poorest, and this could primarily be responsible for weight gain.~~

. One possible explanation for this is that in developing countries, individuals with higher wealth are more inclined to consume energy-dense foods and lead sedentary lifestyles (40, 72). Moreover, women in higher wealth quantiles might have a higher caloric intake compared to those in lower quantiles, which could be a primary factor contributing to weight gain.

The odds of overweight/obesity among urban women of reproductive age women waswere higher compared with women in the rural areaareas. This finding is supported by previous studies (8, 10, 31, 3816, 21, 41). Research revealed that urbanization accounts for significant shifts in dietary patterns. Besides, the availability of fast-food restaurants that provide energy-dense foods (69) and the availability of supermarkets might be associated with overweight/obesity. In addition, it alters physical activity levels as urban residents frequently use transportation rather than walking. On the other hand, rural residents might have great exposure to physical activities as there are no abundant transportations such as a taxi (31). Therefore, due to the above reasons urban residents of reproductive age women could be more subjected to overweight/obesity than rural residents.

In. Research has shown that urbanization significantly influences dietary patterns. The presence of fast-food restaurants offering energy-dense foods (72) and supermarkets might contribute to the higher rates of overweight/obesity. Additionally, urbanization tends to decrease physical activity levels as residents often rely on transportation instead of walking. In contrast, rural residents are more exposed to physical activities due to limited transportation options such as taxis (16). Therefore, for the reasons mentioned above, urban women of reproductive age might be more susceptible to overweight/obesity compared to their rural counterparts.

In low and middle-income countries with high maternal mortality, overweigt/obesity of married or, widowed, or, divorced women waswere found to have a higher thanprevalence of overweight /obesity compared to single women. This observation is consistent with previous studies conducted in various settings (8, 10, 16, 19-21). The possible explanation for this finding is supported bythat women who are not single might have a greater likelihood of having had multiple children in previous several studies conducted elsewhere (8, 10, 31, 33, 36, 38). This could be attributed by married or widowed or, divorced women rather than single women are more likely to deliver more children with their repective pregnancy. As a resultpregnancies. Consequently, these women aremight be at higha higher risk of weight gain during theirand after pregnancy and postpartum period due to theira more sedentary lifelifestyle and takingconsumption of energy--dense foods

~~(69, 70). Our study also revealed that the odds of overweight/obesity among reproductive age women who had children were higher than women who had no child. This finding is supported by studies carried out~~(72, 73). Our study also indicated that the odds of overweight/obesity among women of reproductive age who had children were higher than those who had not given birth. This finding aligns with studies conducted in Cambodia ~~(31)~~(16) and Guinea (45). The ~~possible justification~~rationale behind this association could be that women ~~who have~~with children might ~~waste their~~have less time ~~and burdened by childcare, consequently, they might not have appropriate attention~~ for managing their weight ~~management~~due to the demands of childcare ~~(31). Additionally, having more children is directly related with repeated pregnancy and this pregnancy will also be linked with depression and anxiety. Therefore, the depression and anxiety might contribute for hypothalamic-pituitary abnormal hyperactivity which inturn induces abnormal obesity~~(16). Furthermore, having more children is associated with multiple pregnancies, which in turn might lead to depression and anxiety. These psychological factors could potentially contribute to abnormal obesity through hypothalamic-pituitary abnormal hyperactivity (~~71~~74).

Field Code Changed

~~This study revealed that the odds of overweight/obesity was higher among female household heads than their counterparts. This can be explained by when females being the head of household, they will be suffered from stress, depression, and anxiety (72). In this regard, stress, depression, and anxiety are linked with abnormal hormonal function which will increases abnormal weight gain (18, 71). Another finding of this study showed that the odds of overweight/obesity were higher amonge reproductive age women who had improved source of drinking water than who had unimproved source. One of the component of improved source of drinking water which has taken the highest share in this study was borehole water. As a matter of fact, this borehole water is salty water. Therefore, women with higher salt intake might have a probability of higher caloric intake, food consumption, eating more meat and snakes which directly predisposes to obesity (73) could be one of the possible justification. Besides, the other possible explanation could be since bottled water was the other component of improved source of drinking water in this study, the chemicals of plastic of the bottles might contributed to develop weight gain. The chemicals might change users metabolism by provoking the growth of fat cells or adipocytes (74). Surprisingly, as a confirmatory finding in this study, 94% of bottled water user reproductive age women had overweight/obesity.~~

The study revealed that female heads of households were more likely to be overweight or obese compared to other household heads. This could be attributed to the stress, depression, and anxiety experienced by female household heads (75). These conditions are linked to abnormal hormonal function, which can lead to unhealthy weight gain (27, 74). Additionally, the study found that reproductive-age women with improved sources of drinking water had higher odds of being overweight or obese compared to those with unimproved sources. The study highlighted borehole water as a significant component of improved drinking water sources, and this water is known to be salty. Therefore, women with higher salt intake might have a greater likelihood of increased caloric intake, food consumption, and consumption of meat and snacks, which directly contributes to obesity (76). Another potential explanation could be the presence of bottled water as part of the improved drinking water sources in the study. The study suggested that the chemicals in plastic bottles might contribute to weight gain by altering metabolism and promoting the growth of fat cells. Interestingly, the study confirmed that 94% of reproductive-age women who used bottled water were overweight or obese (77).

Our study had some strengths and limitations. Among the strengths of this study~~;~~, it was analyzed using pooled data from 21 nationally representative DHS surveys in low and middle-income countries with high maternal mortality. In addition, we weighted our sample size to get reliable estimates and standard errors. As a limitation, the DHS used a cross-sectional survey design, and the causal relationship between reproductive~~—~~-age women's overweight/obesity and the independent variables cannot be established.

Conclusion and recommendation

Formatted: Line spacing: 1.5 lines

The prevalence of overweight/obesity among women of reproductive age ~~women~~ (15-49 years old) was found to be relatively high in low and middle-income countries with high maternal mortality. The result indicated that more than three out of ten ~~reproductive age~~ women in this age group were overweight/obese. ~~Individual~~Various individual-level and community-level factors were associated with overweight/obesity. ~~Reproductive~~Women of reproductive age ~~women with~~who were older~~ age, higher education, higher wealth status, having ~~, more educated, wealthier, had children, ~~watching~~watched television, ~~using~~used the internet, ~~having~~had access to improved ~~source of~~ drinking water sources, and lived in urban ~~residents had higher odds of~~ areas were more likely to be overweight~~/obesity. The~~ or obese. Additionally, the odds of being overweight~~/obesity~~

~~was also~~ or obese were higher ~~in~~for female ~~household~~ heads~~, not~~ of households, non-working women, married, widowed, and divorced women. ~~Giving~~It is recommended to pay special attention to ~~those groups~~women of reproductive age ~~women~~ who ~~are aged, had attended~~have received formal education, ~~not working women, who are watching~~are not in the workforce, engage in television viewing and ~~using~~ internet~~,~~ usage, live in urban ~~residents,~~areas, are female ~~household head~~heads of households, and ~~richest~~are among the wealthiest group of women ~~are recommended~~.

Abbreviations

AOR, Adjusted Odds Ratio; BMI, body mass index; CI, Confidence Interval; CDC, Centers for Disease Control and Prevention; CHF, Congestive Heart Failure; CKD, Chronic Kidney ~~Diease~~Disease; DHSs, Demographic and Health Surveys; DALYs disability-adjusted life years; ICC, Intracluster correlation coefficient; IR, individual record; IRB, Institutional Review Board; LMIC, Low and Middle~~-~~-Income Countries; MOR, median odds ratio; MUAC, Mid-Upper Arm Circumference; PCV, a proportional change in variance; UNICEF, United Nations International Children's Emergency Fund; VIF, Variance Inflation Factor; WHO, World Health Organization

Declarations

Consent for publication

Not applicable.

Availability of data and materials

The data used for the analysis of this study is attached as supplementary file 1.

~~Data are available online in a public, open access repository (www.measuredhs.com/data)~~.

Competing interests

The authors declare that they have no competing interests.

Funding

No funder.

Author Contributions

Conceptualization: DMG. Study design: DMG, KMA, YT, AE, WDN, AMW, LY, MG, NW, AYB, LA, HTA, MGT, AH, MJF, and KAD. Execution: DMG, KMA, YT, AE, WDN, AMW, LY, MG, NW, AYB, LA, HTA, MGT, AH, MJF, and KAD. Acquisition of the data: DMG, KMA, YT, AE, WDN, AMW, LY, MG, NW, AYB, LA, HTA, MGT, AH, MJF, and KAD. Analysis and interpretation: DMG, KMA, YT, AE, WDN, AMW, LY, MG, NW, AYB, LA, HTA, MGT, AH, MJF, and KAD. Writing: DMG, KMA, YT, AE, WDN, AMW, LY, MG, NW, AYB, LA, HTA, MGT, AH, MJF, and KAD. Review and editing: DMG, KMA, YT, AE, WDN, AMW, LY, MG, NW, AYB, LA, HTA, MGT, AH, MJF, and KAD. All authors have read and approved the final version of the manuscript.

Formatted: Font: Times New Roman, 12 pt

Acknowledgments

The authors would like to thank measure DHS for their permission to access the datasedataset.

References

1. Organization WH. Diet, nutrition, and the prevention of chronic diseases: report of a joint WHO/FAO expert consultation: World Health Organization; 2003.
2. Lim SS, Vos T, Flaxman AD, Danaei G, Shibuya K, Adair-Rohani H, et al. A comparative risk assessment of burden of disease and injury attributable to 67 risk factors and risk factor clusters in 21 regions, 1990–2010: a systematic analysis for the Global Burden of Disease Study 2010. The lancet. 2012;380(9859):2224-60.
3. Afshin A, Forouzanfar MH, Reitsma MB, Sur P, Estep K, Lee A, et al. Health Effects of Overweight and Obesity in 195 Countries over 25 Years. The New England journal of medicine. 2017;377(1):13-27.
4. Ford ND, Patel SA, Narayan KM. Obesity in Low- and Middle-Income Countries: Burden, Drivers, and Emerging Challenges. Annual review of public health. 2017;38:145-64.
5. Swinburn BA, Sacks G, Hall KD, McPherson K, Finegood DT, Moodie ML, ~~et al~~Gortmaker SL. The global obesity pandemic: shaped by global drivers and local environments. The Lancet. 2011;378(9793):804-14.
6. Ng M, Fleming T, Robinson M, Thomson B, Graetz N, Margono C, et al. Global, regional, and national prevalence of overweight and obesity in children and adults during 1980–2013: a systematic analysis for the Global Burden of Disease Study 2013. The lancet. 2014;384(9945):766-81.
7. Chowdhury MAB, Adnan MM, Hassan MZ. Trends, prevalence and risk factors of overweight and obesity among women of reproductive age in Bangladesh: a pooled analysis of five national cross-sectional surveys. BMJ open. 2018;8(7):e018468.
8. Sartorius B, Veerman LJ, Manyema M, Chola L, Hofman K. Determinants of Obesity and Associated Population Attributability, South Africa: Empirical Evidence from a National Panel Survey, 2008-2012. PloS one. 2015;10(6):e0130218.
9. Neupane S, KC P, Doku DT. Overweight and obesity among women: analysis of demographic and health survey data from 32 Sub-Saharan African Countries. BMC public health. 2015;16(1):1-9.
10. Mkuu R, Barry A, Yonga G, Nafukho F, Wernz C, Gilreath T, et al. Prevalence and factors associated with overweight and obesity in Kenya. Preventive medicine reports. 2021;22:101340.
11. Djalalinia S, Yoosefi M, Shahin S, Ghasemi E, Rezaei N, Ahmadi N, et al. The levels of BMI and patterns of obesity and overweight during the COVID-19 pandemic: Experience from the Iran STEPs 2021 survey. Frontiers in Endocrinology. 2022;13:1043894.
12. Mendez MA, Monteiro CA, Popkin BM. Overweight exceeds underweight among women in most developing countries. The American journal of clinical nutrition. 2005;81(3):714-21.
13. Jones-Smith JC, Gordon-Larsen P, Siddiqi A, Popkin BM. Is the burden of overweight shifting to the poor across the globe? Time trends among women in 39 low-and middle-income countries (1991–2008). International journal of obesity. 2012;36(8):1114-20.
14. Lyrio AO, Souza ES, dos S Conceição S, Batista JE, Brito SM, Gomes Filho IS, et al. Prevalence of overweight and obesity and associated factors among women of childbearing age in Brazil. Public health nutrition. 2021;24(16):5481-90.
15. Ali NB, Dibley MJ, Islam S, Rahman MM, Raihana S, Bhuiyan SET, et al. Overweight and obesity among urban women with iron deficiency anaemia in Bangladesh. Maternal & Child Nutrition. 2021;17(2):e13102.

Formatted: Line spacing: single

16. Um S, Yom A, A. Muir J, Sopheab H. Overweight and obesity among women at reproductive age in Cambodia: Data analysis of Cambodia Demographic and Health Survey 2014. PLOS global public health. 2023;3(3):e0001768.

17. Talukder A, Gupta RD, Hashan MR, Haider SS, Sajal IH, Sarker M. Association between television viewing and overweight and obesity among women of reproductive age in Timor-Leste: evidence from the demographic health survey 2016. BMJ open. 2021;11(8):e045547.

18. Asosega KA, Aidoo EN, Adebanji AO, Owusu-Dabo E. Examining the risk factors for overweight and obesity among women in Ghana: A multilevel perspective. Heliyon. 2023;9(5).

19. Mosha D, Paulo HA, Mwanyika-Sando M, Mboya IB, Madzorera I, Leyna GH, et al. Risk factors for overweight and obesity among women of reproductive age in Dar es Salaam, Tanzania. BMC nutrition. 2021;7(1):1-10.

20. Seidu A-A, Ahinkorah BO, Agbaglo E, Nyaaba AA. Overweight and obesity among women of reproductive age in Mali: what are the determinants? International Health. 2021;13(5):428-35.

21. Mukora-Mutseyekwa F, Zeeb H, Nengomasha L, Kofi Adjei N. Trends in prevalence and related risk factors of overweight and obesity among women of reproductive age in Zimbabwe, 2005–2015. International Journal of Environmental Research and Public Health. 2019;16(15):2758.

22. Kinnunen TI, Bastola K, Neupane S. Trends in the prevalence of overweight and obesity among women of reproductive age. 2021.

23. Manson JE, Willett WC, Stampfer MJ, Colditz GA, Hunter DJ, Hankinson SE, et al. Body weight and mortality among women. New England Journal of Medicine. 1995;333(11):677-85.

~~15~~24. Cefalu WT, Bray GA, Home PD, Garvey WT, Klein S, Pi-Sunyer FX, et al. Advances in the science, treatment, and prevention of the disease of obesity: reflections from a diabetes care editors' expert forum. Diabetes Care. 2015;38(8):1567-82.

~~16~~25. Capodaglio P, Liuzzi A. Obesity: a disabling disease or a condition favoring disability? 2013.

~~17~~26. Doherty DA, Magann E, Francis J, Morrison J, Newnham J. Pre-pregnancy body mass index and pregnancy outcomes. International Journal of Gynecology & Obstetrics. 2006;95(3):242-7.

~~18~~27. Kulie T, Slattengren A, Redmer J, Counts H, Eglash A, Schrager S. Obesity and women's health: an evidence-based review. The Journal of the American Board of Family Medicine. 2011;24(1):75-85.

~~19~~28. Leeners B, Rath W, Kuse S, Irawan C, Imthurn B, Neumaier-Wagner P. BMI: new aspects of a classical risk factor for hypertensive disorders in pregnancy. Clinical Science. 2006;111(1):81-6.

~~20~~29. Bergström A, Hsieh C, Lindblad P, Lu C, Cook N, Wolk A. Obesity and renal cell cancer–a quantitative review. British journal of cancer. 2001;85(7):984-90.

~~21~~30. Yeh T-L, Hsu H-Y, Tsai M-C, Hsu L-Y, Hwang L-C, Chien K-L. Association between metabolically healthy obesity/overweight and cardiovascular disease risk: a representative cohort study in Taiwan. PloS one. 2021;16(2):e0246378.

~~22~~31. Raatikainen K, Heiskanen N, Heinonen S. Transition from overweight to obesity worsens pregnancy outcome in a BMI-dependent manner. Obesity. 2006;14(1):165-71.

~~23~~32. Seligman LC, Duncan BB, Branchtein L, Gaio DSM, Mengue SS, Schmidt MI. Obesity and gestational weight gain: cesarean delivery and labor complications. Revista de saude publica. 2006;40(3):457-65.

2433. Jiang K, Chen B, Lou D, Zhang M, Shi Y, Dai W, et al. Systematic review and meta-analysis: association between obesity/overweight and surgical complications in IBD. International Journal of Colorectal Disease. 2022;37(7):1485-96.

2534. Kristensen J, Vestergaard M, Wisborg K, Kesmodel U, Secher NJ. Pre-pregnancy weight and the risk of stillbirth and neonatal death BJOG: An International Journal of Obstetrics and Gynaecology 112 (4), 403–408. 2008.

2635. Ehrenberg HM, Mercer BM, Catalano PM. The influence of obesity and diabetes on the prevalence of macrosomia. American journal of obstetrics and gynecology. 2004;191(3):964-8.

2736. Nucci LB, Schmidt MI, Duncan BB, Fuchs SC, Fleck ET, Britto MMS. Nutritional status of pregnant women: prevalence and associated pregnancy outcomes. Revista de saude publica. 2001;35(6):502-7.

2837. Graves BW, DeJoy SA, Heath A, Pekow P. Maternal body mass index, delivery route, and induction of labor in a midwifery caseload. Journal of midwifery & women's health. 2006;51(4):254-9.

2938. Marchi J, Berg M, Dencker A, Olander E, Begley C. Risks associated with obesity in pregnancy, for the mother and baby: a systematic review of reviews. Obesity Reviews. 2015;16(8):621-38.

3039. Bo S, Menato G, Signorile A, Bardelli C, Lezo A, Gallo M, et al. Obesity or diabetes: what is worse for the mother and for the baby? Diabetes & metabolism. 2003;29(2):175-8.

31. Um S, Yom A, A. Muir J, Sopheab H. Overweight and obesity among women at reproductive age in Cambodia: Data analysis of Cambodia Demographic and Health Survey 2014. PLOS global public health. 2023;3(3):e0001768.

32. Talukder A, Gupta RD, Hashan MR, Haider SS, Sajal IH, Sarker M. Association between television viewing and overweight and obesity among women of reproductive age in Timor-Leste: evidence from the demographic health survey 2016. BMJ open. 2021;11(8):e045547.

33. Seidu A-A, Ahinkorah BO, Agbaglo E, Nyaaba AA. Overweight and obesity among women of reproductive age in Mali: what are the determinants? International Health. 2021;13(5):428-35.

34. Asosega KA, Aidoo EN, Adebanji AO, Owusu-Dabo E. Examining the risk factors for overweight and obesity among women in Ghana: A multilevel perspective. Heliyon. 2023;9(5).

35. Ali NB, Dibley MJ, Islam S, Rahman MM, Raihana S, Bhuiyan SET, et al. Overweight and obesity among urban women with iron deficiency anaemia in Bangladesh. Maternal & Child Nutrition. 2021;17(2):e13102.

36. Mosha D, Paulo HA, Mwanyika-Sando M, Mboya IB, Madzorera I, Leyna GH, et al. Risk factors for overweight and obesity among women of reproductive age in Dar es Salaam, Tanzania. BMC nutrition. 2021;7(1):1-10.

37. Lyrio AO, Souza ES, dos S Conceição S, Batista JE, Brito SM, Gomes-Filho IS, et al. Prevalence of overweight and obesity and associated factors among women of childbearing age in Brazil. Public health nutrition. 2021;24(16):5481-90.

38. Mukora-Mutseyekwa F, Zeeb H, Nengomasha L, Kofi Adjei N. Trends in prevalence and related risk factors of overweight and obesity among women of reproductive age in Zimbabwe, 2005–2015. International Journal of Environmental Research and Public Health. 2019;16(15):2758.

39. Kinnunen TI, Bastola K, Neupane S. Trends in the prevalence of overweight and obesity among women of reproductive age. 2021.

40. Shayo GA, Mugusi FM. Prevalence of obesity and associated risk factors among adults in Kinondoni municipal district, Dar es Salaam Tanzania. BMC public health. 2011;11(1):1-7.

41. Yeshaw Y, Kebede SA, Liyew AM, Tesema GA, Agegnehu CD, Teshale AB, et alAlem AZ. Determinants of overweight/obesity among reproductive age group women in Ethiopia: multilevel analysis of Ethiopian demographic and health survey. BMJ open. 2020;10(3):e034963.

42. Gigante DP, Moura ECd, Sardinha LMV. Prevalence of overweight and obesity and associated factors, Brazil, 2006. Revista de saude publica. 2009;43:83-9.

43. Enyew EB, Birhanu AY, Mewosha WZ. Spatial variation of overweight/obesity and associated factor among reproductive age group women in Ethiopia, evidence from EDHS 2016. PloS one. 2022;17(11):e0277955.

44. Kumar P, Mangla S, Kundu S. Inequalities in overweight and obesity among reproductive age group women in India: evidence from National Family Health Survey (2015–16). BMC Women's Health. 2022;22(1):205.

45. Diakité D, Sidibé S, Kolié F, Camara F, Camara DM, Kourouma M, et alDélamou A. Prevalence and factors associated with the coexistence of overweight/obesity and anaemia among women of reproductive age in Guinea. Public Health Nutrition. 2023:1-10.

46. Worku M, Gizaw Z, Kassahun Belew A, Wagnew A, Hunegnaw MT. Prevalence and associated factors of overweight and obesity among high school adolescents in Bahir Dar City, Northwest, Ethiopia: A cross-sectional study. Journal of Obesity. 2021;2021.

47. Li M, Gong W, Wang S, Li Z. Trends in body mass index, overweight and obesity among adults in the USA, the NHANES from 2003 to 2018: A repeat cross-sectional survey. BMJ open. 2022;12(12):e065425.

48. Omer I, Derese T, Sintayehu Y. Overweight and Its Associated Factors among Women of Reproductive Age in Dire Dawa, Eastern Ethiopia, 2021: Community-Based Cross-Sectional Study. Journal of Obesity. 2022;2022.

49. Larsen TB, Sørensen HT, Gislum M, Johnsen SP. Maternal smoking, obesity, and risk of venous thromboembolism during pregnancy and the puerperium: a population-based nested case-control study. Thrombosis research. 2007;120(4):505-9.

50. Dagne S, Gelaw YA, Abebe Z, Wassie MM. Factors associated with overweight and obesity among adults in northeast Ethiopia: a cross-sectional study. Diabetes, metabolic syndrome and obesity: targets and therapy. 2019;12:391.

51. Monteiro CA. Nutrition and health. The issue is not food, nor nutrients, so much as processing. Public Health Nutrition. 2009;12(5):729-31.

52. Rivera JA, Barquera S, Gonzalez-Cossio T, Olaiz G, Sepulveda J. Nutrition transition in Mexico and in other Latin American countries. Nutrition reviews. 2004;62(suppl_2):S149-S57.

53. Rutstein SO, Rojas G. Guide to DHS statistics. Calverton, MD: ORC Macro. 2006;38.

54. Organization WH. Trends in maternal mortality 2000 to 2020: estimates by WHO, UNICEF, UNFPA, World Bank Group and UNDESA/Population Division: World Health Organization Obesity and Overweight; 2023.

5555. Croft T, Marshall A, Allen CK, Arnold F, Assaf S, Balian S, Bekele Y. Guide to DHS statistics: DHS-7 (version 2). Rockville, MD: ICF. 2020.

56. Organization WH. World Health Organization Obesity and Overweight.

57. Getnet M, Sisay WS, Alem AZ. Spatial distribution and associated factors of co-occurrence of overweight/obesity and Anemia among women in the reproductive age in sub-Saharan Africa: A multilevel analysis, DHS 2016–2021. PloS one. 2024;19(4):e0299519.

58. Chilot D, Belay DG, Ferede TA, Shitu K, Asratie MH, Ambachew S, et al. Pooled prevalence and determinants of antenatal care visits in countries with high maternal mortality: A multi-country analysis. Frontiers in Public Health. 2023;11:1035759.

~~56~~59. Chilot D, Mondelaers A, Alem AZ, Asres MS, Yimer MA, Toni AT, ~~et al~~Ayele TA. Pooled prevalence and risk factors of malaria among children aged 6–59 months in 13 sub-Saharan African countries: A multilevel analysis using recent malaria indicator surveys. PloS one. 2023;18(5):e0285265.

~~57~~60. Malik VS, Willett WC, Hu FB. Global obesity: trends, risk factors and policy implications. Nature reviews endocrinology. 2013;9(1):13-27.

~~58~~61. Dereje D, Yirgu R, Chichiabellu T. Magnitude of overweight/obesity and associated factors among high school adolescents' in Addis Ababa, Ethiopia. J Nutr Disord Ther. 2018;8(2):2161-0509.

~~59~~62. Alemu E, Atnafu A, Yitayal M, Yimam K. Prevalence of overweight and/or obesity and associated factors among high school adolescents in Arada Sub city, Addis Ababa, Ethiopia. Journal of nutrition & food sciences. 2014;4(2):1.

~~60~~63. Delacosta TC. Explorando associações entre sarcopenia, obesidade e osteoporose: estudo com pacientes da atenção primária em saúde. 2019.

~~61~~64. Santos VRd, Araujo MYC, Cardoso MR, Batista VC, Christofaro DGD, Gobbo LA. Association of insufficient physical activity with sarcopenia and sarcopenic obesity in individuals aged 50 years or more. Revista de Nutrição. 2017;30:175-84.

~~62~~65. Athukorala C, Rumbold AR, Willson KJ, Crowther CA. The risk of adverse pregnancy outcomes in women who are overweight or obese. BMC pregnancy and childbirth. 2010;10(1):1-8.

~~63~~66. Holowko N, Mishra G, Koupil I. Social inequality in excessive gestational weight gain. International journal of obesity. 2014;38(1):91-6.

~~64~~67. Bhurosy T, Jeewon R. Overweight and obesity epidemic in developing countries: a problem with diet, physical activity, or socioeconomic status? The Scientific World Journal. 2014;2014.

~~65~~68. Monda KL, Adair LS, Zhai F, Popkin BM. Longitudinal relationships between occupational and domestic physical activity patterns and body weight in China. European journal of clinical nutrition. 2008;62(11):1318-25.

~~66~~69. Sugiyama T, Healy GN, Dunstan DW, Salmon J, Owen N. Is television viewing time a marker of a broader pattern of sedentary behavior? Annals of behavioral Medicine. 2008;35(2):245-50.

~~67~~70. Abolfotouh MA, Al-Alwan IA, Al-Rowaily MA. Prevalence of metabolic abnormalities and association with obesity among Saudi college students. International journal of hypertension. 2012;2012.

~~68~~71. Cleland VJ, Schmidt MD, Dwyer T, Venn AJ. Television viewing and abdominal obesity in young adults: is the association mediated by food and beverage consumption during viewing time or reduced leisure-time physical activity? The American journal of clinical nutrition. 2008;87(5):1148-55.

~~69~~72. Abrha S, Shiferaw S, Ahmed KY. Overweight and obesity and its socio-demographic correlates among urban Ethiopian women: evidence from the 2011 EDHS. BMC Public Health. 2016;16:1-7.

Formatted: Line spacing: single

~~70~~73. Huayanay-Espinoza CA, Quispe R, Poterico JA, Carrillo-Larco RM, Bazo-Alvarez JC, Miranda JJ. Peer Reviewed: Parity and Overweight/Obesity in Peruvian Women. Preventing chronic disease. 2017;14.

~~71~~74. Pasquali R, Vicennati V, Cacciari M, Pagotto U. The hypothalamic-pituitary-adrenal axis activity in obesity and the metabolic syndrome. Annals of the New York Academy of Sciences. 2006;1083(1):111-28.

~~72~~75. Yoosefi Lebni J, Mohammadi Gharehghani MA, Soofizad G, Khosravi B, Ziapour A, Irandoost SF. Challenges and opportunities confronting female-headed households in Iran: a qualitative study. BMC women's health. 2020;20:1-11.

~~73~~76. Navia B, Aparicio A, Perea JM, Pérez-Farinós N, Villar-Villalba C, Labrado E, ~~et al~~Ortega RM. Sodium intake may promote weight gain; results of the FANPE study in a representative sample of the adult Spanish population. Nutrición hospitalaria. 2014;29(6):1283-9.

~~74~~77. Darbre PD. Endocrine disruptors and obesity. Current obesity reports. 2017;6:18-27.

~~Figure Legendes~~

Figures Legends

Figure 1: Flow chart showing the sampling procedure of DHS data of reproductive age women in countries with high maternal mortality

Figure ~~1~~2: Prevalence of overweight/obesity of reproductive age women in countries with high maternal mortality

Supplementary file 1: Data used for the analysis of this study.

Point-by-point response for editor's and reviewers' comments

	Editor's and Reviewers' comments and suggestions	**Authors' responses**
	Editor's comments and suggestions	
1.	Thank you for submitting your manuscript to PLOS ONE. After careful consideration, we feel that it has merit but does not fully meet PLOS ONE's publication criteria as it currently stands. Therefore, we invite you to submit a revised version of the manuscript that addresses the points raised during the review process.	Dear Editor, thank you for your consideration and suggestion! We revised the manuscript according to your comments and suggestions.
2.	I have extensibly annotated the original comments and provided feedback about changes to be done in the manuscript in each section. My main concern is about pooling data. First, DHSs data use a two-stage sampling design. When data are pooled I wonder which weights have been used to estimates coefficients (AOR)?	Dear Editor, thank you for your concern and comment. Pooled data analysis is a methodology of analyzing data from different sources which has the advantage of producing more accurate estimation and greater statistical power. Besides, it helps to generalize findings from different populations. Therefore, by considering the above importance, we conducted pooled data analysis to determine the magnitude of overweight/obesity only. However, we used normal (non-pooled) overweight/obesity categorized (Yes/No) data to conduct multilevel multivariable regression analysis. Hence, we used normal (non-pooled) data to identify the associated factors of the dependent variable and their statistical parameters/coefficients/ such as AOR, COR, P-value, and 95% CI.<br>Dear editor, as we have tried to describe in the above sentences as to our knowledge and understanding we gave more value for pooled data rather than non-pooled data due to its strong nature of analysis and evidence. In this

		case, we kindly hope you can understand our intention and this description will answer your comments of pooled data analysis which were raised throughout the document.
3.	Second, the outcome variable is not well operationalized (see manuscript for comments). Are underweight women excluded from analyses. This is unclear from the manuscript. It that is the case, then this introduces additional statistical concern because when estimates are done on sub-populations, additional care should be done to ensure that representativeness of samples are preserved.	Dear editor, thank you for your comment, for the purpose of further clarity, we have revised and merged the “variable definition” and “operational definition” sections and labeled as “variables and measurements”. Besides, your raised concerns are addressed as per your suggestion, please see the clean manuscript on pages 8-10, lines 169 - 210.<br>**Regarding the inclusion/exclusion of underweight women**: As we know, our analysis should be done based on our title/topic. Due to this reason, we didn’t include underweight (mildly, moderately and severely thin) women both in the numerator and in the denominator because we are focused to study about the magnitude and associated factors of overweight/obesity as mentioned in the topic. The dependent variable was fixed based on our previous practice in related article [1], other previous published literature [2], and WHO classification [3]. Therefore, as to our knowledge and the practices, there will not be a statistical concern because the dependent variable was fixed by strictly following the standard DHS guide and previous articles.
4.	Third, the discussion section is very flawed since it is like a "redo" of results section. This section should have been more clearer and useful if the authors engaged discussing factors associated with overweight/obesity at (i) individual level; and (ii) community level. The authors should really engage in key findings and how they align	Dear editor, thank you for your comment and concern, we revised the discussion according to your suggestion by considering the addressable issues, please see the revised section of the discussion.

	or differ from what is already known on the subject;	
5.	Fourth, authors are referring to high maternal mortality countries without any references on the adopted classification.	Dear editor, thank you very much for your constructive comment, we have mentioned the source of this classification and we also included in the variables and measurements section for further understanding. We hope this definition will answer all comments regarding high maternal mortality, please see the revised clean manuscript on page 10, lines 208-210 and on page 8, line 166.
6.	Fifth, the manuscript requires serious edits.	Dear editor, thank you for your constructive comments, we have edited and revised each section of the manuscript which needs edition.
7.	Why should countries be interested in pooled prevalence? Pragmatically and policy-wise, this is less relevant!	Dear editor, thank you for your comment! All the included countries share the same source of information (DHS survey) and for this reason this pooled analysis will have pragmatic policy implications. Thus, to get holistic picture about overweight/obesity among reproductive age women in countries with high maternal mortality need to be evaluated empirically for up-to-date intervention by respective country governments and other partners working on maternal health.
8.	Clarify! What is high maternal mortality?	Thank you for your question, we have clarified it accordingly. Maternal mortality is considered to be high if it is 300-499, very high if it is 500-999, and extremely high if it is equal to and higher than 1000 maternal deaths per 100,000 live births. For the sake of maintaining economy of words and attractiveness in the abstract we have clarified it in the variables and measurements section, please see the updated clean manuscript on page 10, lines 208-210.

9.	DHSs don't allow causality; therefore determinant is inappropriate	Dear editor, thank you for your suggestion, we have revised the whole manuscript which states about determinants according to your valuable suggestion, please see the updated manuscript.
10.	This is fuzzy in an abstract. Not easy to get a sense of the message conveyed in this long sentence	Dear editor, thank you for your constructive comment, for the sake of conciseness in the abstract we tried to shorten it by mentioning factors having more strong association. Please see the revised section of the abstract on page 3, lines 65 to 70. Our assumption when we omit these less strong factors is that readers can get the detail of all factors on the result and discussion section.
11.	General comment: The English is poor and needs serious improvement!	Dear editor, thank you for your comment, we have revised and edited the entire manuscript by language expert to improve the language of the manuscript.<br>Besides, we revised the background section according to your specific comments and tried to include these specific comments' response in this point-by-point response.
12.	This kind of construction breaks the flow of the paper. Talk first of prevalence from previous studies globally, in LMICs, and at country-level. Thereafter, move to consequences, and associated factors.	Dear editor, thank you for your insights and improvement suggestion, we revised according to your suggestion, please see the revised clean manuscript on page 5-6, lines 94-120.
13.	It is not watching TV per se. It is about "sedentary behaviour"	Dear editor, you are correct, media usage in general is related with sedentary behavior which in return affects overweight and we have clearly stated on the possible justification of the discussion section. However, in the background section, for the purpose of specificity we have presented as frequency of watching television.
14.	what's the added value of pooled prevalence	Dear editor, thank you for your clarification question! Pooled data analysis is a methodology of analyzing data from different sources which has the advantage of producing more accurate estimation and greater statistical

		power. Besides, it helps to generalize findings from different populations. Therefore, by considering the above importance, we conducted pooled data analysis to determine the magnitude.
15.	Readers were expecting till now to know (understand) how high maternal mortality was defined. Nothing but speculations	Dear editor, thank you for your comment again, we defined in the “variables and measurement” section.
16.	When data are pooled, how did the authors incorporate the complex survey design (CSD) in DHSs?	Dear editor, thank you for your question, as mentioned in the method part of the manuscript, cross-sectional study design was used in all Demographic and Health Surveys. Therefore, there will no be a problem of complex survey design.
17.	This is what? It is well known that DHSs collect information on women of reproductive ages (15-49 years). What this sentence adds up?	Dear editor, yes you are correct it didn’t have value and thank you for your valuable comment, we removed it.
18.	Data sources for these statistics ?	Dear editor, thank you for your question, we mentioned the data source, please see at the bottom of table 1. We have used three data sources as follows: http://www.dhsprogram.com, http://srhr.org/mmr2020, or http://www.who.int/publications/i/item/9789240068759
19.	Whose classification is it?	Dear editor, thank you for your clarification question, this is the classification of WHO, UNICEF, and world bank. We have cited the appropriate reference, please see the revised clean manuscript on page 8, line 166.
20.	This is vague while this key for the manuscript. How was it collected in DHSs? How was it treated in this paper? How the decision to operationalize the original variable was taken ?	Dear editor, thank you for your valuable comments and insights, we revised it according to your comment, please see the “variable and measurement” section on pages 8-9, lines 171-185.
21.	This should go to "outcome variable?"	Dear editor, thank you for your valuable suggestion, we revised it accordingly, please see on pages 8-9, lines 176-179.
22.	why capitalize?	Thank you for your question! We had a practice of capitalizing the first spelling of abbreviated phrases and

		that is why we capitalized the first spellings.
23.	This is debatable since even non-bivariate associations could become "significant" in multivariable analyses. this choice should be justified	Dear editor, thank you for your comment! Yes, it is debatable, however, as the practice of different scholars we used 0.25 as a criterion to filter our candidate variables for multilevel regression analysis.
24.	This section is too log for secondary data analysis	Dear editor, yes you are correct, it is long. However, we did this to answer the questions which were raised by the PLOS ONE journal assistants during technical check as journal requirement.
25.	For binary variables and to make Table clearer, knowing "one category" reveals info on the other category.	Dear editor, thank you for your comment and you are correct.
26.	I wonder whether these 2 sub-headings are necessary (Random vs. Fixed effects)	Dear editor, thank you for your suggestion, we corrected is as per your suggestion, please see the revised manuscript on page 15.
27.	This is not the numbers of women of reproductive ages in DHS report? How do we trust these numbers ?	Dear editor, thank you for your clarification question, this is the number of non-pregnant, who didn't have births in the last five years, and non-underweight women which was fixed according to the DHS statistics guide. Additionally, we reached to this number after omitting all missing variables, so it might not be equal to the observed reproductive age women. For further understanding please see the flow chart named as figure 1 and attached as TIF file, please see the uploaded figure.
28.	It should have been a good idea to split discussion into 2 sub-headings: Individual-level factors and community-level factors and better engage the discussion and provide plausible explanations to understand findings	Dear editor, we understand your concerns on the discussion and appreciate your specific comments in the manuscript. However, we face difficulties to split the discussion in to two sub-headings. Because if we split in to two sub-headings there will be redundancy of possible justifications between some individual level and community level factors which might affect the attractiveness of this section. For example; frequency of

		watching television from individual level factors and community-level media usage from community level factor had association with the dependent variable. In these comparable variables we had similar possible justifications. If we split these comparable variables in to different sections and if we state the similar possible justification, repetition of idea might occur.
29.	This is a less productive way to engage in a discussion, which shouldn't be a "redo" of results	Dear editor, thank you for your comment, we presented the factors that need modification in synthesized manner, please see on page 19, lines 299-305.
30.	what's functional performance?	Dear editor, thank you for your clarification point, we revised the full sentence to render full meaning, please see the clean manuscript on page 19, lines 310-311.
31.	This should be dug enough	Dear editor, thank you for your critical insight, we revised it in detail, please see the clean manuscript on page 20, lines 319-330.
	Reviewer 1: comments and suggestions	
1.	The manuscript is good in terms of objective but not an easy article to comprehend. The writing should be improved. For example in the abstract the authors written "Women aged 25–34 years and 35-49 years, women who had primary education, who had secondary education and above, not-working women, married, widowed, and divorced women, poorer, middle, richer, and richest household, female household head, having one child, 2-3 children, 4 and above children, watching television less than once a week and at least once	Dear reviewer, thank you very much for your valuable comment, we modified this sentence, please see the abstract on page 3, lines 65-70.

	a week, using internet less than once a week and at least once a week, improved source of drinking water, urban residents, low community poverty, and high community level media usage." -- it is not a complete sentence.	
2.	I attempted to find the country-specific prevalence of obesity but was unsuccessful in the manuscript. It will be helpful to know the country-specific prevalence before pooling the results.	Dear reviewer, thank you for your comment, the country specific prevalence was done and stated on figure 2 which was uploaded as TIF file separately, please see the uploaded figure.
3.	I recommend that the authors incorporate a discussion of their results in the context of previous studies.	Dear reviewer, thank you in advance for your recommendation, we have discussed the magnitude from our result and the associated factors from previous studies. We have discussed the magnitude from our result purposely because our study is about pooled prevalence and we didn't found pooled prevalence of overweight/obesity form previous studies. In this case if we compare our pooled prevalence from previous single prevalence studies, this will lead us to compare incomparable magnitudes/findings. This is the reason why we had discussed the magnitude by using our result only. However, we have discussed the associated factors from previous studies in detail which is inline with your valuable recommendation.
	Reviewer 2: comments and suggestions	
1.	If the initially considered countries were low- and middle-income countries, modify the title to include "high maternal mortality in low and middle-income countries."	Dear reviewer, thank you very much for your insight, we revised the title according to your constructive comment. Besides, the rest of the manuscript is also modified according to the slight modification in the title.
2.	A flowchart illustrating the number of countries	Dear reviewer, thank you very much for your important

	initially considered, and how 21 countries with a sample size of 64,076 were reached, will make the sampling process easier to understand.	suggestion, we prepared the flowchart according to your valuable suggestion which was uploaded as TIF file separately, please see the uploaded figure 1.
3.	I also suggest that the outcome variable be overweight/obesity and dichotomized as yes/no.	Dear reviewer, thank you in advance again for your valuable suggestion. We revised the manuscript according to your valuable suggestion, please see on page 9, lines 179-181.

References for this authors' response

1. Getnet M, Sisay WS, Alem AZ: **Spatial distribution and associated factors of co-occurrence of overweight/obesity and Anemia among women in the reproductive age in sub-Saharan Africa: A multilevel analysis, DHS 2016–2021**. *PloS one* 2024, **19**(4):e0299519.
2. Yeshaw Y, Kebede SA, Liyew AM, Tesema GA, Agegnehu CD, Teshale AB, Alem AZ: **Determinants of overweight/obesity among reproductive age group women in Ethiopia: multilevel analysis of Ethiopian demographic and health survey**. *BMJ open* 2020, **10**(3):e034963.
3. Organization WH: **World Health Organization Obesity and Overweight**. In.
