# Supplementary material for: Pooled prevalence and factors of overweight/obesity among women of reproductive age in low and middle-income countries with high maternal mortality: A multi-level analysis of recent demographic and health surveys
Source: PLoS One. 2025 Oct 8;20(10):e0316962. doi: 10.1371/journal.pone.0316962 (PMC12507220; doi:10.1371/journal.pone.0316962)
Supplement: Attachment — Submitted filename: Review 2.pdf [file pone.0316962.s007.pdf]

Subject: Evaluation of Revised Manuscript

Dear Editor,

I appreciate the opportunity to review the revised manuscript entitled "Pooled Prevalence and Factors of Overweight/Obesity Among Women of Reproductive Age in Low- and Middle-Income Countries with High Maternal Mortality: A Multi-Level Analysis of Recent Demographic and Health Surveys."

Upon thorough examination of the revisions and the authors' responses to the initial reviewers' comments, I am pleased to convey that all concerns have been adequately addressed. The authors have enhanced the manuscript significantly through their revisions, and I find it now suitable for publication.

Thank you for allowing me to participate in the review of this vital research. Should you require any further insights or clarifications, please feel free to reach out.

Best regards,
