# Supplementary material for: Pooled prevalence and factors of overweight/obesity among women of reproductive age in low and middle-income countries with high maternal mortality: A multi-level analysis of recent demographic and health surveys
Source: PLoS One. 2025 Oct 8;20(10):e0316962. doi: 10.1371/journal.pone.0316962 (PMC12507220; doi:10.1371/journal.pone.0316962)
Supplement: Attachment — Submitted filename: review file.pdf [file pone.0316962.s009.pdf]

Review of the Manuscript: PONE-D-23-42732R3

The manuscript, titled "Pooled prevalence and factors of overweight/obesity among women of reproductive age in low and middle-income countries with high maternal mortality: A multi-level analysis of recent Demographic and Health Surveys," presents an important topic. The study effectively highlights the prevalence and determinants of overweight/obesity among women in LMICs with high maternal mortality. The statistical approach is well-defined, and the multilevel analysis is appropriate. However, a few concerns remain, particularly regarding clarity in result presentation and language consistency. As I am a new reviewer to this manuscript, in addition to reviewing the manuscript, I have also examined whether the issues raised by the previous reviewer have been addressed or not. I appreciate that the authors have taken the comments of previous reviewer into consideration and made changes in the manuscript. Here is the detailed review of the manuscript:

- **Line 67**: The authors have revised the phrasing of associations to indicate relationships between variables instead of categories, addressing the previous reviewer's concern. However, minor rewording for clarity would enhance readability.
- **Line 73-76:** The recommendation section mentions targeting "older women, those with formal education, non-working women, individuals who spend time watching television and using the internet, urban residents, female household heads, and women in the wealthiest households."
    - This contradicts the previous reviewer's concern that the **highest socioeconomic group may not be the most urgent target** (previous reviewer's comment). The phrasing should be revised to reflect a more balanced intervention approach.
- **Line 92-93:** The statement about overweight/obesity increasing among lower economic groups over time should be further clarified. Does this apply to all LMICs or only a subset?
- **Line 131-137:** The justification for the study's objective is clear, but it would help to specify how this study improves on existing literature in terms of methodology or scope.
- **Line 145-146**: The sentence listing countries has been revised, addressing the previous reviewer's concern about the use of "includes."
- **Line 223-225**: The classification of maternal mortality cut-offs using WHO guidelines has been included. This addresses the previous reviewer's request to explain why African countries dominate the dataset. However, a brief mention of why high maternal mortality is particularly relevant to obesity research would strengthen the rationale.
- **Line 240-241**: The authors checked multicollinearity (VIF < 5) to ensure that age and number of living children could be analyzed independently. This adequately addresses the previous reviewer's concern. However, it may be beneficial to explain how interaction effects were considered, if applicable.

Line 272-275: The pooled prevalence of overweight/obesity is well reported. However, it might be useful to include a brief mention of **which factors contribute to variations between countries** to strengthen the analysis.

- **Line 279-282:** The discussion of ICC and MOR in the null model is useful. However, the **interpretation of ICC (19%)** could be expanded to explain whether this level suggests a strong or moderate clustering effect.
- **Line 296-300:** The separation of individual and community-level factors is well done. However, the **inclusion of community poverty** as a determinant still does not fully address the previous reviewer's concern about targeting higher-income groups. The discussion should acknowledge this limitation.
- **Line 373-376**: The authors have moved the discussion of internet use as a proxy for higher socioeconomic status to the discussion section instead of the summary of results. This revision is appropriate. The authors could further acknowledge that **internet use itself is not a direct cause of obesity, but rather a proxy for wealth and lifestyle behaviours.**
- **Line 386-390:** The revision addressing **the difference between obesity patterns in developing vs. developed countries** is well-written. However, the **phrase "low community poverty"** is still potentially misleading. Consider replacing it with **"higher economic status at the community level."**
- **Line 406-408**: The authors acknowledge the possibility of reverse causation regarding marital status and obesity and provide a brief discussion on this. This improves the credibility of their analysis.
- **Statistical Models and Interpretation (Table 4, Multilevel Logistic Regression Analysis, Page 30-31)**: The authors provided a detailed breakdown of their models, but further discussion on effect sizes and practical implications of findings could enhance clarity.

Recommendations:

- The discussion still suggests that the **wealthiest groups should be the focus of intervention,** contradicting trends in lower-income groups. Further justification with stronger evidence would help.
- The term "low community poverty" is misleading and should be replaced.
- ICC interpretation should be more explicit about its strength.

With these minor refinements, the manuscript would be significantly improved. The methodological rigor and data analysis remain sound, making the study a valuable contribution to public health research in LMICs.

Overall, the authors have made most of the necessary changes, but a final round of refinements would improve readability and interpretation.
