# Supplementary material for: Pooled prevalence and factors of overweight/obesity among women of reproductive age in low and middle-income countries with high maternal mortality: A multi-level analysis of recent demographic and health surveys
Source: PLoS One. 2025 Oct 8;20(10):e0316962. doi: 10.1371/journal.pone.0316962 (PMC12507220; doi:10.1371/journal.pone.0316962)
Supplement: Attachment — Submitted filename: Response to reviewers.doc [file pone.0316962.s006.doc]

**Point-by-point response for editor’s and reviewers' comments**

|  | Editor’s and Reviewers’ comments and suggestions | **Authors' responses** |
| --- | --- | --- |
|  | Editor’s comments and suggestions |  |
| 1. | Thank you for submitting your manuscript to PLOS ONE. After careful consideration, we feel that it has merit but does not fully meet PLOS ONE’s publication criteria as it currently stands. Therefore, we invite you to submit a revised version of the manuscript that addresses the points raised during the review process. | Dear Editor, thank you for your consideration and suggestion! We revised the manuscript according to your comments and suggestions. |
| 2. | I have extensibly annotated the original comments and provided feedback about changes to be done in the manuscript in each section. My main concern is about pooling data. First, DHSs data use a two-stage sampling design. When data are pooled I wonder which weights have been used to estimates coefficients (AOR)? | Dear Editor, thank you for your concern and comment. Pooled data analysis is a methodology of analyzing data from different sources which has the advantage of producing more accurate estimation and greater statistical power. Besides, it helps to generalize findings from different populations. Therefore, by considering the above importance, we conducted pooled data analysis to determine the magnitude of overweight/obesity only. However, we used normal (non-pooled) overweight/obesity categorized (Yes/No) data to conduct multilevel multivariable regression analysis. Hence, we used normal (non-pooled) data to identify the associated factors of the dependent variable and their statistical parameters/coefficients/ such as AOR, COR, P-value, and 95% CI.  Dear editor, as we have tried to describe in the above sentences as to our knowledge and understanding we gave more value for pooled data rather than non-pooled data due to its strong nature of analysis and evidence. In this case, we kindly hope you can understand our intention and this description will answer your comments of pooled data analysis which were raised throughout the document. |
| 3. | Second, the outcome variable is not well operationalized (see manuscript for comments). Are underweight women excluded from analyses. This is unclear from the manuscript. It that is the case, then this introduces additional statistical concern because when estimates are done on sub-populations, additional care should be done to ensure that representativeness of samples are preserved. | Dear editor, thank you for your comment, for the purpose of further clarity, we have revised and merged the “variable definition” and “operational definition” sections and labeled as “variables and measurements”. Besides, your raised concerns are addressed as per your suggestion, please see the clean manuscript on pages 8-10, lines 169 -210.  **Regarding the inclusion/exclusion of underweight women**: As we know, our analysis should be done based on our title/topic. Due to this reason, we didn’t include underweight (mildly, moderately and severely thin) women both in the numerator and in the denominator because we are focused to study about the magnitude and associated factors of overweight/obesity as mentioned in the topic. The dependent variable was fixed based on our previous practice in related article [1], other previous published literature [2], and WHO classification [3]. Therefore, as to our knowledge and the practices, there will not be a statistical concern because the dependent variable was fixed by strictly following the standard DHS guide and previous articles. |
| 4. | Third, the discussion section is very flawed since it is like a "redo" of results section. This section should have been more clearer and useful if the authors engaged discussing factors associated with overweight/obesity at (i) individual level; and (ii) community level. The authors should really engage in key findings and how they align or differ from what is already known on the subject; | Dear editor, thank you for your comment and concern, we revised the discussion according to your suggestion by considering the addressable issues, please see the revised section of the discussion. |
| 5. | Fourth, authors are referring to high maternal mortality countries without any references on the adopted classification. | Dear editor, thank you very much for your constructive comment, we have mentioned the source of this classification and we also included in the variables and measurements section for further understanding. We hope this definition will answer all comments regarding high maternal mortality, please see the revised clean manuscript on page 10, lines 208-210 and on page 8, line 166. |
| 6. | Fifth, the manuscript requires serious edits. | Dear editor, thank you for your constructive comments, we have edited and revised each section of the manuscript which needs edition. |
| 7. | Why should countries be interested in pooled prevalence? Pragmatically and policy-wise, this is less relevant! | Dear editor, thank you for your comment! All the included countries share the same source of information (DHS survey) and for this reason this pooled analysis will have pragmatic policy implications. Thus, to get holistic picture about overweight/obesity among reproductive age women in countries with high maternal mortality need to be evaluated empirically for up-to-date intervention by respective country governments and other partners working on maternal health. |
| 8. | Clarify! What is high maternal mortality? | Thank you for your question, we have clarified it accordingly. Maternal mortality is considered to be high if it is 300-499, very high if it is 500-999, and extremely high if it is equal to and higher than 1000 maternal deaths per 100,000 live births. For the sake of maintaining economy of words and attractiveness in the abstract we have clarified it in the variables and measurements section, please see the updated clean manuscript on page 10, lines 208-210. |
| 9. | DHSs don't allow causality; therefore determinant is inappropriate | Dear editor, thank you for your suggestion, we have revised the whole manuscript which states about determinants according to your valuable suggestion, please see the updated manuscript. |
| 10. | This is fuzzy in an abstract. Not easy to get a sense of the message conveyed in this long sentence | Dear editor, thank you for your constructive comment, for the sake of conciseness in the abstract we tried to shorten it by mentioning factors having more strong association. Please see the revised section of the abstract on page 3, lines 65 to 70. Our assumption when we omit these less strong factors is that readers can get the detail of all factors on the result and discussion section. |
| 11. | General comment: The English is poor and needs serious improvement! | Dear editor, thank you for your comment, we have revised and edited the entire manuscript by language expert to improve the language of the manuscript.  Besides, we revised the background section according to your specific comments and tried to include these specific comments’ response in this point-by-point response. |
| 12. | This kind of construction breaks the flow of the paper. Talk first of prevalence from previous studies globally, in LMICs, and at country-level. Thereafter, move to consequences, and associated factors. | Dear editor, thank you for your insights and improvement suggestion, we revised according to your suggestion, please see the revised clean manuscript on page 5-6, lines 94-120. |
| 13. | It is not watching TV per se. It is about "sedentary behaviour" | Dear editor, you are correct, media usage in general is related with sedentary behavior which in return affects overweight and we have clearly stated on the possible justification of the discussion section. However, in the background section, for the purpose of specificity we have presented as frequency of watching television. |
| 14. | what's the added value of pooled prevalence | Dear editor, thank you for your clarification question! Pooled data analysis is a methodology of analyzing data from different sources which has the advantage of producing more accurate estimation and greater statistical power. Besides, it helps to generalize findings from different populations. Therefore, by considering the above importance, we conducted pooled data analysis to determine the magnitude. |
| 15. | Readers were expecting till now to know (understand) how high maternal mortality was defined. Nothing but speculations | Dear editor, thank you for your comment again, we defined in the “variables and measurement” section. |
| 16. | When data are pooled, how did the authors incorporate the complex survey design (CSD) in DHSs? | Dear editor, thank you for your question, as mentioned in the method part of the manuscript, cross-sectional study design was used in all Demographic and Health Surveys. Therefore, there will no be a problem of complex survey design. |
| 17. | This is what? It is well known that DHSs collect information on women of reproductive ages (15-49 years). What this sentence adds up? | Dear editor, yes you are correct it didn’t have value and thank you for your valuable comment, we removed it. |
| 18. | Data sources for these statistics ? | Dear editor, thank you for your question, we mentioned the data source, please see at the bottom of table 1. We have used three data sources as follows: [http://www.dhsprogram.com](http://www.dhsprogram.com/), <http://srhr.org/mmr2020>, or http://www.who.int/publications/i/item/9789240068759 |
| 19. | Whose classification is it? | Dear editor, thank you for your clarification question, this is the classification of WHO, UNICEF, and world bank. We have cited the appropriate reference, please see the revised clean manuscript on page 8, line 166. |
| 20. | This is vague while this key for the manuscript. How was it collected in DHSs? How was it treated in this paper? How the decision to operationalize the original variable was taken ? | Dear editor, thank you for your valuable comments and insights, we revised it according to your comment, please see the “variable and measurement” section on pages 8-9, lines 171-185. |
| 21. | This should go to "outcome variable?" | Dear editor, thank you for your valuable suggestion, we revised it accordingly, please see on pages 8-9, lines 176-179. |
| 22. | why capitalize? | Thank you for your question! We had a practice of capitalizing the first spelling of abbreviated phrases and that is why we capitalized the first spellings. |
| 23. | This is debatable since even non-bivariate associations could become "significant" in multivariable analyses. this choice should be justified | Dear editor, thank you for your comment! Yes, it is debatable, however, as the practice of different scholars we used 0.25 as a criterion to filter our candidate variables for multilevel regression analysis. |
| 24. | This section is too log for secondary data analysis | Dear editor, yes you are correct, it is long. However, we did this to answer the questions which were raised by the PLOS ONE journal assistants during technical check as journal requirement. |
| 25. | For binary variables and to make Table clearer, knowing "one category" reveals info on the other category. | Dear editor, thank you for your comment and you are correct. |
| 26. | I wonder whether these 2 sub-headings are necessary (Random vs. Fixed effects) | Dear editor, thank you for your suggestion, we corrected is as per your suggestion, please see the revised manuscript on page 15. |
| 27. | This is not the numbers of women of reproductive ages in DHS report? How do we trust these numbers ? | Dear editor, thank you for your clarification question, this is the number of non-pregnant, who didn’t have births in the last five years, and non-underweight women which was fixed according to the DHS statistics guide. Additionally, we reached to this number after omitting all missing variables, so it might not be equal to the observed reproductive age women. For further understanding please see the flow chart named as figure 1 and attached as TIF file, please see the uploaded figure. |
| 28. | It should have been a good idea to split discussion into 2 sub-headings: Individual-level factors and community-level factors and better engage the discussion and provide plausible explanations to understand findings | Dear editor, we understand your concerns on the discussion and appreciate your specific comments in the manuscript. However, we face difficulties to split the discussion in to two sub-headings. Because if we split in to two sub-headings there will be redundancy of possible justifications between some individual level and community level factors which might affect the attractiveness of this section. For example; frequency of watching television from individual level factors and community-level media usage from community level factor had association with the dependent variable. In these comparable variables we had similar possible justifications. If we split these comparable variables in to different sections and if we state the similar possible justification, repetition of idea might occur. |
| 29. | This is a less productive way to engage in a discussion, which shouldn't be a "redo" of results | Dear editor, thank you for your comment, we presented the factors that need modification in synthesized manner, please see on page 19, lines 299-305. |
| 30. | what's functional performance? | Dear editor, thank you for your clarification point, we revised the full sentence to render full meaning, please see the clean manuscript on page 19, lines 310-311. |
| 31. | This should be dug enough | Dear editor, thank you for your critical insight, we revised it in detail, please see the clean manuscript on page 20, lines 319-330. |
|  | Reviewer 1: comments and suggestions |  |
| 1. | The manuscript is good in terms of objective but not an easy article to comprehend. The writing should be improved. For example in the abstract the authors written "Women aged 25–34 years and 35-49 years, women who had primary education, who had secondary education and above, not-working women, married, widowed, and divorced women, poorer, middle, richer, and richest household, female household head, having one child, 2-3 children, 4 and above children, watching television less than once a week and at least once a week, using internet less than once a week and at least once a week, improved source of drinking water, urban residents, low community poverty, and high community level media usage." -- it is not a complete sentence. | Dear reviewer, thank you very much for your valuable comment, we modified this sentence, please see the abstract on page 3, lines 65-70. |
| 2. | I attempted to find the country-specific prevalence of obesity but was unsuccessful in the manuscript. It will be helpful to know the country-specific prevalence before pooling the results. | Dear reviewer, thank you for your comment, the country specific prevalence was done and stated on figure 2 which was uploaded as TIF file separately, please see the uploaded figure. |
| 3. | I recommend that the authors incorporate a discussion of their results in the context of previous studies. | Dear reviewer, thank you in advance for your recommendation, we have discussed the magnitude from our result and the associated factors from previous studies. We have discussed the magnitude from our result purposely because our study is about pooled prevalence and we didn’t found pooled prevalence of overweight/obesity form previous studies. In this case if we compare our pooled prevalence from previous single prevalence studies, this will lead us to compare incomparable magnitudes/findings. This is the reason why we had discussed the magnitude by using our result only. However, we have discussed the associated factors from previous studies in detail which is inline with your valuable recommendation. |
|  | Reviewer 2: comments and suggestions |  |
| 1. 1 | If the initially considered countries were low- and middle-income countries, modify the title to include "high maternal mortality in low and middle-income countries." | Dear reviewer, thank you very much for your insight, we revised the title according to your constructive comment. Besides, the rest of the manuscript is also modified according to the slight modification in the title. |
| 2. 2 | A flowchart illustrating the number of countries initially considered, and how 21 countries with a sample size of 64,076 were reached, will make the sampling process easier to understand. | Dear reviewer, thank you very much for your important suggestion, we prepared the flowchart according to your valuable suggestion which was uploaded as TIF file separately, please see the uploaded figure 1. |
| 3. | I also suggest that the outcome variable be overweight/obesity and dichotomized as yes/no. | Dear reviewer, thank you in advance again for your valuable suggestion. We revised the manuscript according to your valuable suggestion, please see on page 9, lines 179-181. |

**References for this authors’ response**

1.	Getnet M, Sisay WS, Alem AZ: **Spatial distribution and associated factors of co-occurrence of overweight/obesity and Anemia among women in the reproductive age in sub-Saharan Africa: A multilevel analysis, DHS 2016–2021**. *PloS one* 2024, **19**(4):e0299519.

2.	Yeshaw Y, Kebede SA, Liyew AM, Tesema GA, Agegnehu CD, Teshale AB, Alem AZ: **Determinants of overweight/obesity among reproductive age group women in Ethiopia: multilevel analysis of Ethiopian demographic and health survey**. *BMJ open* 2020, **10**(3):e034963.

3.	Organization WH: **World Health Organization Obesity and Overweight**. In*.*
