# Supplementary material for: Pooled prevalence and factors of overweight/obesity among women of reproductive age in low and middle-income countries with high maternal mortality: A multi-level analysis of recent demographic and health surveys
Source: PLoS One. 2025 Oct 8;20(10):e0316962. doi: 10.1371/journal.pone.0316962 (PMC12507220; doi:10.1371/journal.pone.0316962)
Supplement: Attachment — Submitted filename: Response_to_reviewers_auresp_2.doc [file pone.0316962.s008.doc]

**Point-by-point response for editor’s and reviewers' comments**

|  | Editor’s and Reviewers’ comments and suggestions | **Authors' responses** |
| --- | --- | --- |
|  | Editor’s comments and suggestions |  |
| 1. | Thank you for submitting your manuscript to PLOS ONE. After careful consideration, we feel that it has merit but does not fully meet PLOS ONE’s publication criteria as it currently stands. Therefore, we invite you to submit a revised version of the manuscript that addresses the points raised during the review process. | Dear Editor, We would like to extend our sincere thanks to you and the reviewers for your invaluable feedback and critical insights during the two rounds of revisions. Your thoughtful comments have greatly contributed to the improvement of our manuscript, helping to enhance its quality and clarity. We deeply appreciate the time and effort you and the reviewers invested in providing detailed suggestions, which have led to significant improvements in the content and overall structure of the paper. We have carefully addressed all the feedback and revised the manuscript accordingly. Thank you once again for your support and guidance. |
| 2. | This means what? Should you really expect same number of children for all women? | Dear Editor, thank you for your clarification request. We did not intend to suggest that all women would have the same number of children. The use of the word "different" was meant to highlight the variation across categories. However, to avoid any confusion for readers, we have revised the sentence accordingly. Please refer to the revised section of the abstract in the clean version of the manuscript, located on page 3, line 69. |
| 3. | This is not TRUE since the study is done, injustifiably, on a sub-sample of women (normal, overweight/obese) | Dear editor, thank you for your concerns, in the current study, we recalculated the sampling weights specifically for the sub-sample of women with valid Body Mass Index (BMI) data, excluding those categorized as underweight. The original DHS weights were designed to apply to the full survey population, but because not all women had BMI data, and our focus was on women with normal, overweight, or obese BMI levels, recalculating the weights ensured that our sub-sample remained representative of the population of interest. This approach preserves the integrity of the DHS complex survey design by maintaining accurate weighting adjustments for the primary sampling units (PSUs) and strata. Please kindly see the updated version of the manuscript on pages 7-8, lines 162-166. |
| 4. | 1) The study excluded, unjustifiably, from the study underweighted women. Similar studies (see reference provided in annotated manuscript, did not excluded underweigthed women. Authors based this irrationale decision on studies conducted in Ethiopia. This needs to be addressed. | Dear Editor, thank you in advance for your thoughtful comments and feedback. Genuinely, all of your comments and suggestions are really invaluable for our manuscript significant improvement and we are lucky to get such vibrant research scientist.  We appreciate your concern regarding the exclusion of underweight women from the study. This decision was made with the aim of focusing specifically on the distinct public health challenges associated with overweight and normal-weight women. We acknowledge that underweight women face their own unique health risks, but including them in our analysis could have diluted our findings and shifted the focus away from the primary aim of addressing the rising burden of **overweight and obesity** (which is one of the current neglected public health problem specially in developing countries) in the population under study.  Although some studies have included underweight women, several authors have similarly chosen to exclude this group when concentrating on overweight and obesity to allow for a clearer understanding of the risk factors and health outcomes associated with these conditions. Our decision is in line with this practice, as we wanted to provide targeted insights on these critical public health concerns without compromising the specificity of the results.  Nevertheless, we recognize this exclusion as a limitation of our study and have clearly acknowledged this in the manuscript, please kindly see on page 27, lines 420-421. We understand that future research should explore the specific challenges of underweight women, but our current focus was on the health risks of overweight and obesity. |
| 5. | 2) In either case, analyses are based on sub-samples in each country because not all women have information on BMI. Therefore, using svyset, and svy are not enough to account for complex sampling design. This is also need to addressed correctly. | Dear editor, thank you for your concerns, we would like to confirm that the issue of sub sampling and complex sampling design has been already addressed.  The DHS weights are originally designed for the full sample. However, since not all women had BMI data, we recalculated the sampling weights specifically for the sub-sample of women with valid BMI data. This adjustment ensures that the sub-sample remains representative of the population in each country while maintaining the integrity of the survey’s complex design.  We understand your concern that simply applying the **svyset** and **svy** commands may not fully account for complex sampling. To address this, we updated the **svyset** and **svy** commands with the recalculated weights to correctly reflect the primary sampling units (PSUs) and strata for the sub-sample. This process ensures that the complex sampling design is properly accounted for, even when working with a sub-sample.  We revised the manuscript according to the above explanation, please kindly see the updated version of the manuscript on pages 7-8, lines 162-166. |
| 6. | 3) Many variables at community-level were collected at woman-level. Authors need to clarify how these variables were aggregated at community-level.  All these variables were collected at woman-level. Clearly explicit how aggregation was done. | Dear editor, thank you very much for your valuable suggestions. We have addressed your concerns in the updated version of the manuscript. Please kindly see the updated manuscript on pages 10-11, lines 197-211. |
| 7. | 4) The paper lacks bivariate analyses. From Table 2 (descriptives of the sample), the paper jumps to multi-level analyses.  This Table is not well designed. Use categories in same column with variables  Additionally, this Table should include the dependent variable ?  Indeed, Table 3 jumps into modeling without any indication of bivariate analyses ???? | Dear editor, thank you in advance for your invaluable suggestions and comments. We have addressed all of your concerns in the updated version of the manuscript. Please kindly see the updated manuscript on pages 15-18, lines 265-270. |
| 8. | Why so many variables on medias? You should have created an index of media exposure as most studies do? | Dear Editor, thank you very much for your suggestion and guidance. However, we put media variables in separate manner purposely unlike previous studies. The reason of putting them in separate format is to clearly delineate their specific individual effect on the outcome of interest. We believe analyzing such specific association might have an advantage of rendering specific recommendation for each media component. |
| 9. | Why do you need this elliptic format? | Dear editor, thank you for your question, we used this elliptic format to visibly differentiate form the rest of the boxes. However, if it has an effect of confusing readers, we adjusted it in to rectangle, please kindly see the revised flowchart. |
| 10. | The direction of the arrow does not make sense  Exclude should go out not IN | Dear editor, thank you for your valuable suggestion, we adjusted it according to your suggestion, please kindly see the revised flowchart. |
| 11. | This last number is unnecessary and misleading | Dear editor, thank you for your comment, however, since this last number is the weighted sample which was used for each of our analysis, omitting this value might confuse the readers because the readers might use the unweighted frequency incorrectly. As a result, as to our understanding, mentioning in the flowchart might be advantageous. |
