# Supplementary material for: Pooled prevalence and factors of overweight/obesity among women of reproductive age in low and middle-income countries with high maternal mortality: A multi-level analysis of recent demographic and health surveys
Source: PLoS One. 2025 Oct 8;20(10):e0316962. doi: 10.1371/journal.pone.0316962 (PMC12507220; doi:10.1371/journal.pone.0316962)
Supplement: Attachment — Submitted filename: Response_to_reviewers_auresp_3.doc [file pone.0316962.s010.doc]

**Point-by-point response for editor’s and reviewers' comments**

| Sr.No | Editor’s and Reviewers’ comments and suggestions | **Authors' responses** |
| --- | --- | --- |
|  | Editor’s comments and suggestions | |
| 1. | **The reviewer has raised a number of major concerns, particularly around the methods section and statistical approach, as well as the presentation of the results. Could you please carefully revise the manuscript to address all comments raised?** | Dear Editor, We would like to extend our sincere thanks to you and the reviewers for your invaluable feedback and critical insights during this the third rounds of revisions. Since we got comments only from reviewer 4 (***the other reviewers didn’t have comments at this stage of revision process***), we have carefully addressed the feedback and revised the manuscript accordingly. |
|  | Reviewer four’s comments and suggestions | |
| 1 | I come to this paper as a new reviewer on this revision and struggle with the way the findings are worded here. The associations are between variables and not between given categories (abstract); the issue here of the risk factors being potentially associated- use of internet may imply a higher socioeconomic grouping - needs to be dealt with in the summary of the results. | Dear reviewer, thank you in advance for your valuable suggestions. Yes, you are write, the association is between the dependent and independent variables and we have adjusted the confusing sentence in order not to confuse other further readers. Please kindly see the updated manuscript at the abstract section on page 3, line 67.  Regarding the relationship between use of internet and higher socioeconomic grouping, thank you again for your insight and we have addressed your comment at the discussion section (in order not to congest the summary of results, we prefer to incorporate at the discussion part). Please kindly see the updated manuscript at the discussion section on page 22, lines 373-376. |
| 2 | The idea that the highest socioeconomic group needs more targeting is against the evidence showing an increasing issue in the lower groups - as this may the group where the targeting may have best effect. | Dear reviewer, thank you for your comment. If we get your idea, we have said that socio-economic status and weight gain has direct relationship i.e the richest are developing more weight gain than the poorest in developing countries. Therefore, we kindly request you to understand our description as we didn’t say the lower socio-economic group are related with an increasing issue. Probably, if we made confusion by the phrase “low community poverty”, having low community poverty indicates higher economic status than high community poverty. For further clarification, please kindly see the revised “variables and measurement” section on page 10, lines 216-218.  Moreover, the previous articles suggested that socio-economic status has different effect on weight gain of developing and developed countries and for further clarification, we have modified the discussion section please kindly see on page 23, lines 386-390..  However, since our study is focusing on developing countries, our major analysis and interpretation is mainly targeting to these developing countries. |
| 3 | Please do not use "includes" for countries but list all countries in the study. | Dear reviewer, thank you very much for your suggestion. We modified the sentence according to your suggestion. Please kindly see the updated manuscript at the method section on page 7, lines 145-146. |
| 4 | There is a preponderance of African countries here - please explain the cut-offs used that cause a concentration on these countries and why other countries do not qualify. | Dear reviewer, thank you for your clarification question. Our study is conducted by focusing only on countries with high maternal mortality. We used WHO cut-off value to classify maternal mortality as follows: ***The maternal mortality ratio (MMR) is categorized as high if it falls within the range of 300-499, very high if it is between 500-999, and extremely high if it equals or exceeds 1000 maternal deaths per 100,000 live births.*** This classification sentence was stated at the “variables and measurement” section, please kindly see it on page 10, lines 223-225. Besides, this cut-off value was mentioned under table 1, please kindly see on page 8, line169.  The reason of focusing on those selected countries was due to the women in such countries seeks an urgent attention to avert causes of morbidity and mortality. Therefore, other countries that have maternal mortalities other than high, very high, and extremely high did not qualify for this study and were excluded. That is why our studied countries were concentrated majorly on Africa. |
| 5 | Above community is country and issues such as conflict, natural disaster etc which are known to impact maternal morbidity and mortality - were these taken into account? | Dear reviewer, thank you for your critical insight. We were planned to conduct sub-group analysis by region in order to address your specific comments, however, by default majority of the countries fall under a single sub-group (sub-Saharan African countries), which was difficult to undertake sub-group analysis since the category didn’t fulfil chi-square assumption. Therefore, we can’t consider country specific variables like conflict and natural disaster. |
| 6 | One may argue there is reverse causation in things like marital status - how was this dealt with here? | Dear reviewer, thank you for your comment. Your critical comment improved our manuscript in advance. Yes! You are write, even if majority of the findings support our research result, there could be a reverse causation and we modified our discussion section according to your valuable comment, please kindly see on page 23, lines 406-408. |
| 7 | Age and number of living children is clearly correlated - how do you unpick this? | Dear reviewer, thank you for your comment and we want to share your idea by considering ourselves as a reviewer. However, as a researcher, we were expected to conduct multicollinearity assessment to identify the relationship between independent variables. Accordingly, the result depicted that there were no issues found as all variables had VIF <5, with the best fitted model (model III) VIF at 1.74, please kindly see on page 11, lines 240-241. Therefore, based on the finding of the above statistical parameter, we analyzed age and number of living children as an independent factors in our final output table. |
| 8 | In what sense can things be explained by older age and lack of poverty - both of which are known to be associated with higher BMI? | Dear reviewer, thank you for your clarification question. Our study includes only the reproductive age group women (15-49 years). The positive association between older age and weight gain might be attributed to physiological changes that occur with age, such as hormonal shifts, increased body fat, and changes in body composition. Additionally, reduced participation in physical activities due to age-related physiological changes among women may contribute to the increased weight gain. Moreover, it might be related with our basal metabolic rate, which is the rate at which our body utilizes energy during at rest to keep vital body functions going, that slow down year to year which resulted in high caloric density and weight gain later on. Furthermore, the shifts in behavior, lifestyle and even emotional health might be related with weight gain in older age. For further clarification, we added more additional possible justifications, please kindly see at the discussion section on page 21, lines 331-335.  When we come to the relationship between lack of poverty and weight gain, you raised an important point and we addressed your concern as follows:  Lack of poverty (high socio-economic status) has different association with weight gain in developing and developed countries. In developing countries, it has direct relationship with weight gain i.e; individuals with higher wealth are more inclined to consume energy-dense foods and lead sedentary lifestyles. Additionally, women in higher wealth quantiles might have a higher caloric intake compared to those in lower quantiles, which could be a primary factor contributing to weight gain.  However, paradoxically in developed countries, there was an inverse association between economic status and weight gain which depicted that lower economic status was related with higher weight gain. The suggested possible justification could be that poor socio-economic groups in developed countries are more likely to be unemployed, lower educated and might have irregular meals. Therefore, according to your constructive suggestion, we revised the discussion section please take a look on page 23, lines 386-390. |
