# Supplementary material for: Pooled prevalence and factors of overweight/obesity among women of reproductive age in low and middle-income countries with high maternal mortality: A multi-level analysis of recent demographic and health surveys
Source: PLoS One. 2025 Oct 8;20(10):e0316962. doi: 10.1371/journal.pone.0316962 (PMC12507220; doi:10.1371/journal.pone.0316962)
Supplement: Attachment — Submitted filename: Response_to_reviewers_auresp_4.doc [file pone.0316962.s011.doc]

**Point-by-point response for editor’s and reviewers' comments**

| Sr.No | Editor’s and Reviewers’ comments and suggestions | **Authors' responses** |
| --- | --- | --- |
|  | Editor’s comments and suggestions | |
| 1. | Thank you for submitting your manuscript to PLOS ONE. After careful consideration, we feel that it has merit but does not fully meet PLOS ONE’s publication criteria as it currently stands. Therefore, we invite you to submit a revised version of the manuscript that addresses the points raised during the review process.  Three reviewers have assessed your revised submission with two requesting additional revisions before your manuscript can be considered for publication. Please carefully respond to all comments, including the nuance and challenges with BMI as an indicator of weight and health. | Dear Editor, thank you for your valuable support in helping to improve the quality of our manuscript. We have carefully considered the reviewers’ comments and invested significant time and research effort to address each point thoroughly. The manuscript has been revised accordingly.  Regarding the nuances and challenges:  Although BMI has certain nuances and limitations as an indicator of weight and health, we used it as a measurement tool for the following reasons:   1. Body Mass Index (BMI) is a widely accepted metric for assessing body weight in relation to height and is commonly used as a standard indicator of overall health; 2. We followed the standard guidelines provided in the DHS (Demographic and Health Surveys) to statistics, where BMI is used as a key measure of the nutritional status of both women and men; 3. Although waist circumference, waist-to-hip-ratio, and body composition analysis are considered as more accurate measure, they are not available in the DHS dataset. Therefore, BMI remains the most appropriate and accessible measure within the DHS data; 4. BMI is widely relied upon in public health and clinical guidelines as a primary indicator for assessing weight-related health risks.   Therefore, the above are sound reasons to use BMI as a measurement in the DHS data set.  However, to notify the more appropriate measurement for future researchers, we included the nuances and challenges as a limitation, please kindly see the limitation section on pages 27-28, lines 459-465. |
|  | Reviewer four’s comments and suggestions | |
| 1. | I'm afraid the issue of categories versus variables remains. The offending line in the abstract is not just line 67 and it is not the issue of the word assocaited. The factors associated are age,marital status, household income, household size etc. Please talk about the variables throughout the paper e.g. al;so the paragraph starting line 293. Connected with this Table 4 should not bold and use stars for significant categories but do an overall test for association at a variable level (see for example the seminal work of Tukey on this for an explanation of why this is not statistically valid). | Dear reviewer, thank you very much for clarifying your previous comment and for giving us this additional opportunity. We initially mentioned each category of variables showing statistically significant associations to provide clearer specification and better understanding for readers. However, in response to your valid and constructive feedback, we have revised the entire document accordingly, please kindly see on the revised manuscript on page 3, lines 67-71 and pages 17-18, lines 303-309.  Regarding the bolding and stars in the categories of Table 4, we sincerely appreciate your insightful comments. We have revised the entire table based on your valuable suggestions and have applied John Tukey’s method accordingly, please kindly see table 4 on page 19-22. |
| 2. | The issue of potentially associated variables needs to be part of the analyses - we know the issue with amyl nitrite and AIDS, and we need to understand whether variable selection plays a part - the issue of what is potentially a confounding factor is a result not a discussion point. | Dear reviewer, thank you for your constructive comment. As our study relied on a secondary data source, the specific variables you mentioned were not available in the dataset. This limitation is inherent to the use of secondary data, and as a result, we were unable to include these important variables in our analysis. |
| 3. | The issue in developed countries and developing countries is the different types of diet that are associated with poverty - this is well known.  While here the higher socioeconomic groups have higer rates of obesity; it would help to understand if there are trends of greater increases over time in lower sciopcioeconomic groups which might indicate that as these are developing bad habits but might be persuaded to change, this is an important group to target. | Dear reviewer, thank you for your kind understanding.  Yes, the difference between developed and developing countries lies in the types of diets commonly consumed by these populations.  The odds ratios are a clear indicative of the increment of obesity i.e; 1.3 (poorer), 1.75 (middle), 1.98 (richer), and 2.38 (richest). That is why we recommend special attention for higher socio-economic group. Regarding these, we have done a slight modification in the discussion, please kindly see on page 25, lines 392-395. |
| 4. | It is known that BMI tends to increase with age even in premenopausal women - this needs to be unpicked as above. | Dear reviewer, thank you for your valuable and critical insight. We have revised the discussion section in accordance with your constructive comment, please kindly see on pages 23-24, lines 336-344. |
|  | Reviewer five’s comments and suggestions |  |
| 1. | The manuscript, titled "Pooled prevalence and factors of overweight/obesity among women of reproductive age in low and middle-income countries with high maternal mortality: A multi-level analysis of recent Demographic and Health Surveys," presents an important topic. The study effectively highlights the prevalence and determinants of overweight/obesity among women in LMICs with high maternal mortality. The statistical approach is well-defined, and the multilevel analysis is appropriate. However, a few concerns remain, particularly regarding clarity in result presentation and language consistency. As I am a new reviewer to this manuscript, in addition to reviewing the manuscript, I have also examined whether the issues raised by the previous reviewer have been addressed or not. I appreciate that the authors have taken the comments of previous reviewer into consideration and made changes in the manuscript. Here is the detailed review of the manuscript: | Dear reviewer, thank you very much for your constructive comments and valuable suggestions. Your critical insights have significantly contributed to improving the quality of our manuscript. We have revised the manuscript in accordance with your comments as well as those from Reviewer 4. Please kindly refer to the revised sections corresponding to each comment. |
| 2. | Line 67: The authors have revised the phrasing of associations to indicate relationships between variables instead of categories, addressing the previous reviewer’s concern. However, minor rewording for clarity would enhance readability. | Dear reviewer, than you in advance for your valuable suggestion. We initially mentioned each category of variables showing statistically significant associations to provide clearer specification and better understanding for readers. However, in response to your constructive feedback and that of Reviewer 4, we have revised the manuscript by replacing the specific categories with the corresponding variables throughout the document, please kindly see on the revised manuscript on page 3, lines 67-71 and pages 17-18, lines 303-309. |
| 3. | Line 73-76: The recommendation section mentions targeting "older women, those with formal education, non-working women, individuals who spend time watching television and using the internet, urban residents, female household heads, and women in the wealthiest households." o This contradicts the previous reviewer's concern that the highest socioeconomic group may not be the most urgent target (previous reviewer's comment). The phrasing should be revised to reflect a more balanced intervention approach. | Dear reviewer, thank you for your constructive suggestion. We revised the recommendation as per your valuable guidance, please kindly see on page 4, lines 77-78. |
| 4. | Line 92-93: The statement about overweight/obesity increasing among lower economic groups over time should be further clarified. Does this apply to all LMICs or only a subset? | Dear reviewer, thank you for your valuable suggestion. This finding is applicable to all 39 low- and middle-income countries included in the study. We have revised the section in accordance with your recommendation, please see on page 5, lines 93-95. |
| 5. | Line 131-137: The justification for the study’s objective is clear, but it would help to specify how this study improves on existing literature in terms of methodology or scope. | Dear reviewer, thank you in advance for your insightful feedback. We have revised the section in line with your valuable suggestion, please kindly see on page 6, lines 133-138. |
| 6. | Line 145-146: The sentence listing countries has been revised, addressing the previous reviewer’s concern about the use of “includes.” | Dear reviewer, thank you very much for your confirmation. |
| 7. | Line 223-225: The classification of maternal mortality cut-offs using WHO guidelines has been included. This addresses the previous reviewer’s request to explain why African countries dominate the dataset. However, a brief mention of why high maternal mortality is particularly relevant to obesity research would strengthen the rationale. | Dear reviewer, thank you in advance for your constructive suggestion, really your valuable comments are significantly improving the quality of our manuscript.  The reason of focusing on those selected countries having high, very high or extremely high maternal mortality was due to the women in such countries seeks an urgent attention to avert causes of morbidity and mortality. We revised the manuscript as per your insightful comment, please kindly see on page 10, lines 232-234. |
| 8. | Line 240-241: The authors checked multicollinearity (VIF < 5) to ensure that age and number of living children could be analyzed independently. This adequately addresses the previous reviewer’s concern. However, it may be beneficial to explain how interaction effects were considered, if applicable. | Dear reviewer, thank you for your question. We believe that testing the assumption of multicollinearity is sufficient for our analysis, as we did not hypothesize that the combined effect of the independent variables would differ from the sum of their individual effects |
| 9. | Line 272-275: The pooled prevalence of overweight/obesity is well reported. However, it might be useful to include a brief mention of which factors contribute to variations between countries to strengthen the analysis. | Dear reviewer, thank you for your comment. However, our objective was to determine the magnitude and associated factors of the dependent variable, rather than to explore the reasons behind the variations in prevalence across the studied countries. Additionally, investigating the causes of such variations would require a separate study with a distinct methodological approach. |
| 10. | Line 279-282: The discussion of ICC and MOR in the null model is useful. However, the interpretation of ICC (19%) could be expanded to explain whether this level suggests a strong or moderate clustering effect. | Dear reviewer, thank you very much for your insightful comment. We revised the manuscript according to your valuable comment, please kindly see on page 17, lines 290-291. |
| 11. | Line 296-300: The separation of individual and community-level factors is well done. However, the inclusion of community poverty as a determinant still does not fully address the previous reviewer’s concern about targeting higher-income groups. The discussion should acknowledge this limitation. | Dear reviewer, thank you for your concern, however, what we are understanding here that the term “lower community poverty” is a confusing phrase. Due to this reason and as per your final recommendation, we modified this phrase as follows:   1. Low community poverty= high community economic status 2. High community poverty=low community economic status   Therefore, we hope this phrase replacement will overcome the confusion and we revised the entire document with this new adjustment, please kindly see the revised manuscript. |
| 12. | Line 373-376: The authors have moved the discussion of internet use as a proxy for higher socioeconomic status to the discussion section instead of the summary of results. This revision is appropriate. The authors could further acknowledge that internet use itself is not a direct cause of obesity, but rather a proxy for wealth and lifestyle behaviours. | Dear reviewer, thank you for your critical insight. We have revised the manuscript as per your valuable recommendation, please kindly see on page 25, lines 388-389. |
| 13. | Line 386-390: The revision addressing the difference between obesity patterns in developing vs. developed countries is well-written. However, the phrase "low community poverty" is still potentially misleading. Consider replacing it with "higher economic status at the community level." | Dear reviewer, thank you very much for your insightful guidance. We truly appreciate your valuable comments, which have significantly improved the quality of our manuscript. We have made the revisions as per your recommendations; please kindly refer to the updated version. |
| 14. | Line 406-408: The authors acknowledge the possibility of reverse causation regarding marital status and obesity and provide a brief discussion on this. This improves the credibility of their analysis. | Dear reviewer, thank you for your understanding. |
| 15. | Statistical Models and Interpretation (Table 4, Multilevel Logistic Regression Analysis, Page 30-31): The authors provided a detailed breakdown of their models, but further discussion on effect sizes and practical implications of findings could enhance clarity. | Dear reviewer, thank you very much for your insightful comment. We have discussed the effect sizes and practical implications of the selected variables at the discussion section, please kindly see on pages 23, lines 344-347; page 25, lines 389-391; pages 25-26, lines 400-403; page 26, lines 417-420; and page 27, lines 436-438. |
| 16. | Recommendations: |  |
| 17. | • The discussion still suggests that the wealthiest groups should be the focus of intervention, contradicting trends in lower-income groups. Further justification with stronger evidence would help. | Dear reviewer, thank you for your recommendation. Since we modified the confusing phrase “low community poverty”, we hope the idea that seems contradiction was solved. |
| 18. | The term "low community poverty" is misleading and should be replaced. | Dear reviewer, thank you very much for your insightful recommendation. We have replaced it with other appropriate and clear phrase, please kindly see the entire document. |
| 19. | ICC interpretation should be more explicit about its strength. | Dear reviewer, thank you in advance for your insightful view. We have done it as per your valuable recommendation, please kindly see on page 17, lines 290-291. |
| 20. | With these minor refinements, the manuscript would be significantly improved. The methodological rigor and data analysis remain sound, making the study a valuable contribution to public health research in LMICs. Overall, the authors have made most of the necessary changes, but a final round of refinements would improve readability and interpretation. | Dear reviewer, thank you sincerely for your thoughtful comments, suggestions, and critical insights. We truly appreciate your contribution to enhancing the quality of our manuscript. We have made every effort to address your valuable feedback and have worked diligently to improve the manuscript accordingly. |
