# Supplementary material for: Pooled prevalence and factors of overweight/obesity among women of reproductive age in low and middle-income countries with high maternal mortality: A multi-level analysis of recent demographic and health surveys
Source: PLoS One. 2025 Oct 8;20(10):e0316962. doi: 10.1371/journal.pone.0316962 (PMC12507220; doi:10.1371/journal.pone.0316962)
Supplement: Attachment — Submitted filename: Response_to_reviewers_auresp_5.doc [file pone.0316962.s012.doc]

**Point-by-point response for editor’s and reviewers' comments**

| Sr.No | Editor’s and Reviewers’ comments and suggestions | **Authors' responses** |
| --- | --- | --- |
|  | Editor’s comments and suggestions | |
| 1. | Thank you for submitting your manuscript to PLOS ONE. After careful consideration, we feel that it has merit but does not fully meet PLOS ONE’s publication criteria as it currently stands. Therefore, we invite you to submit a revised version of the manuscript that addresses the points raised during the review process. | Dear Editor, Thank you for your valuable support in enhancing the quality of our manuscript. We have carefully reviewed the reviewers’ comments and have devoted substantial time and research effort to address each point comprehensively. The entire manuscript has been revised accordingly. |
| 2. | Journal Requirements:  1. If the reviewer comments include a recommendation to cite specific previously published works, please review and evaluate these publications to determine whether they are relevant and should be cited. There is no requirement to cite these works unless the editor has indicated otherwise.   2. Please review your reference list to ensure that it is complete and correct. If you have cited papers that have been retracted, please include the rationale for doing so in the manuscript text, or remove these references and replace them with relevant current references. Any changes to the reference list should be mentioned in the rebuttal letter that accompanies your revised manuscript. If you need to cite a retracted article, indicate the article’s retracted status in the References list and also include a citation and full reference for the retraction notice. | Dear Editor, Thank you in advance for your guidance. We have carefully reviewed all the journal requirements and thoroughly addressed your suggestions. Regarding the citation of retracted articles, we have verified each cited article using the Retraction Watch database (<https://retractiondatabase.org/RetractionSearch.aspx>?) to ensure that no retracted journals have been cited, therefore, we didn’t cite retracted papers. During the review process, we got some miner editorial problem on the reference section and we revised it accordingly. Thank you in advance for your insightful feedback! |
|  | Reviewer four’s comments and suggestions | |
| 1. | 5. Is the manuscript presented in an intelligible fashion and written in standard English?  PLOS ONE does not copyedit accepted manuscripts, so the language in submitted articles must be clear, correct, and unambiguous. Any typographical or grammatical errors should be corrected at revision, so please note any specific errors here.  Reviewer #4: No | Dear reviewer, Thank you for your comments regarding the language of our manuscript. We have invited a language expert and thoroughly revised the entire manuscript to improve its clarity and readability. Kindly refer to the updated version for your review. |
| 2. | Thank you for repsonding to my previous comments. However, in doing so, the English is now in may places unreadable (eg. lines 67-68; and lines 223-5 make no sense at all). It would help if the English were checked for clarity and grammatical correctness. | Dear reviewer, Thank you again for your comments regarding the language of our manuscript. We have invited a language expert and thoroughly revised your specific area comments and entire manuscript to improve its clarity and readability. Please kindly see on page 3, lines 67-68, on page 11 lines 229-231 and refer to the entire updated version. |
|  | Reviewer five’s comments and suggestions |  |
| 1. | After a detailed review of the revised manuscript, it is evident that the authors have thoroughly addressed the comments and recommendations raised by the previous reviewers and the editorial team. Key improvements and responses are summarized below: 1. The authors appropriately justified the use of BMI given the constraints of the DHS dataset, while acknowledging its limitations as a proxy for adiposity. This has been clearly articulated in the limitation section. 2. The manuscript now consistently discusses factors at the variable level, rather than focusing on specific categories (e.g., age, marital status, wealth index), addressing a critical concern of Reviewer 4. Revisions are evident in the abstract, results, and discussion 3. Table 4 has been reformatted to reflect valid statistical comparisons at the variable level using a method inspired by Tukey’s approach. Bold formatting and significance stars have been removed in accordance with statistical best practices. 4. The authors revised the interpretation and recommendations to better balance the focus between higher and lower socioeconomic groups, with added clarity on odds ratios and potential intervention targets. 5. The term “low community poverty” has been corrected throughout, and definitions of community-level factors have been clarified. Multi-collinearity checks were conducted; while interaction effects were not modeled, justification was provided based on study scope. 6. Improvements have been made in terminology, clarity, and presentation of results, including a more balanced discussion of causality (e.g., marital status) and the age-obesity relationship.  All major and minor points raised in previous rounds of peer review appear to have been addressed by the authors. The revised manuscript demonstrates significant improvement and is fit for publication. However, a final proofing pass is advisable before publication to correct minor issues that do not affect scientific integrity. | Dear reviewer, Thank you in advance for confirming that we have addressed your previous comments and concerns. Your insightful feedback has greatly contributed to improving the quality of our manuscript. We have also conducted a final language revision with the assistance of a language expert and completed a thorough proofreading. Please kindly refer to the updated manuscript. |
| 2. | Here is a line-by-line review highlighting minor textual or formatting issues that can be improved before publication. These are not critical but will enhance clarity, consistency, and professionalism of the manuscript.  • Line 75, the phrase “...spend time to watch television and using the internet...” should be rephrased for grammatical consistency. A more appropriate version would be: “...spend time watching television and using the internet...”. | Dear reviewer, Thank you very much for your valuable suggestion. We have revised the manuscript accordingly, please kindly see on page 4, line 76. |
| 3. | Line 78, the expression “optimum care for women from the wealthiest households” is vague. It would be clearer to say: “appropriate health interventions for women from the wealthiest households.” | Dear reviewer, Thank you very much for your valuable suggestion. We have revised the manuscript accordingly, please kindly see on page 4, lines 78-79. |
| 4. | Line 137, consider refining the phrase “a more nuanced understanding” to “an enhanced understanding” for a slightly more formal tone. | Dear reviewer, Thank you for your important suggestion. We have revised the manuscript accordingly, please kindly see on page 6, line 138. |
| 5. | Line 168, the text mentions “...had given birth in the last five years...” but does not clarify why postpartum women were excluded. Providing this rationale would improve the reader’s comprehension of the study’s scope. | Dear reviewer, thank you in advance for your insightful feedback. We have clearly addressed and revised the section in line with your valuable suggestion, please kindly see on page 8, lines 170-174. |
| 6. | Lines 223–224, the phrase “Community poverty was categorized as high community economic status...” is confusing. A clearer version would be: “Communities were categorized as having high or low economic status based on the proportion of women in the poorest wealth quintiles.” | Dear reviewer, thank you very much for your suggestion. We considered your invaluable comment to render clear meaning and to avoid confusion, please kindly see on page 11, lines 229-230. |
| 7. | Throughout the manuscript, phrases like “women age”, “women occupation” “women marital status”, and “women educational status” are grammatically incorrect. These should be changed to “women’s age”, “women’s occupation”, “women’s marital status” and “women’s educational status”, respectively. | Dear reviewer, thank you in advance for your constructive suggestion, really your valuable comments are significantly improving the quality of our manuscript. We revised the entire section according to your valuable suggestion, please kindly see the updated manuscript. |
